# Data on eleven sesquiterpenoids from the cultured mycelia of *Ganoderma capense*

**DOI:** 10.1016/j.dib.2017.04.012

**Published:** 2017-04-19

**Authors:** Zhen Tan, Jinlian Zhao, Jimei Liu, Min Zhang, Ridao Chen, Kebo Xie, Jungui Dai

**Affiliations:** State Key Laboratory of Bioactive Substance and Function of Natural Medicines, Institute of Materia Medica, Chinese Academy of Medical Sciences and Peking Union Medical College, 1 Xian Nong Tan Street, Beijing 100050, People׳s Republic of China

## Abstract

The data included in this paper are associated with the research article entitled “Sesquiterpenoids from the cultured mycelia of *Ganoderma capense*” [1]. ^1^H NMR, ^13^C NMR, DEPT, HSQC, ^1^H–^1^H COSY, HMBC, NOESY, HRESIMS, and IR spectra of Ganodermanol A–H (**1**–**11**), together with Mo_2_(AcO)_4_-induced CD spectrum of Ganodermanol A, CD spectra of Ganodermanol D–E were included in the Data in Brief article. In addition, the cytotoxicities and anti-HIV-1 activity of isolated compounds were also included in the Data in Brief article.

**Specifications Table**

**Table:** 

Subject area	Chemistry
More specific subject area	Natural product
Type of data	Table, figure
How data was acquired	1D and 2D NMR spectra were obtained on a Bruker AVIIID 400/500/600 spectrometers, HRESIMS data were measured using an ESI-FTICR-MS (LTQ-FT Ultra, ThermoFisher Scientific), IR spectra were acquired on a Nicolet 5700 FT-IR microscope spectrometer (FTIR Microscope Transmission), The CD spectra were recorded on a JASCO J-815 spectropolarimeter, the bioassay data were acquired according to the method described in reference 2–3
Data format	analyzed
Experimental factors	Compounds were purified by HPLC.
Experimental features	1D and 2D NMR spectra were obtained on a Bruker AVIIID 400/500/600 spectrometers at 25 °C.
Data source location	Beijing, People׳s Republic of China
Data accessibility	*Ganoderma capense* CGMCC 5.71 was purchased from the China General Microbiological Culture Collection Center (CGMCC, Beijing, China), whose website is http://www.cgmcc.net/.

**Value of the data**

1. These data were very valuable to identify the structure of these compounds.
2. These data can be helpful to research the metabolites of *Ganoderma capense*.
3. Data show the cytotoxicities and anti-HIV-1 activity of isolated compounds.

## 1 Data

^1^H NMR, ^13^C NMR, DEPT, HSQC, ^1^H-^1^H COSY, HMBC, NOESY, HRESIMS, and IR spectra of Ganodermanol A–H (**1**–**11**)[1], together with Mo_2_(AcO)_4_-induced CD spectrum of Ganodermanol A, CD spectra of Ganodermanol D–E were presented in Figs. S1–109, the cytotoxicities and anti-HIV-1 activity of isolated compounds were presented in Tables S1 and 2.

## 2 Experimental design, materials and methods

### 2.1 General experimental procedures

Optical rotations were measured on a Perkin-Elmer Model-343 digital polarimeter. The CD spectra and ORD spectra were recorded on a JASCO J-815 spectropolarimeter. IR spectra were acquired on a Nicolet 5700 FT-IR microscope spectrometer (FTIR Microscope Transmission). 1D and 2D NMR spectra were obtained on a Bruker AVIIID 400/500/600 spectrometers. Chemical shifts (*δ*) are given in ppm, and coupling constants (*J*) are given in hertz (Hz). HRESIMS data were measured using an ESI-FTICR-MS (LTQ-FT Ultra, ThermoFisher Scientific). Silica gel (200–300 mesh, Qingdao Haiyang Chemical Co. Ltd., Qingdao, China) and Sephadex LH-20 gel (Amersham Biosciences, Sweden) and MCI gel (Mitsubishi Chemical Corporation, Japan) were used for column chromatography (CC). Semi-preparative reversed phase and normal phase HPLC were performed on a Shimadzu HPLC instrument equipped with a Shimadzu RID-10A detector and a Shiseido Capcell Pak C_18_ column (250 mm×10 mm, i.d., 5 μm) by eluting with mixtures of methanol and H_2_O at 4.0 mL/min, or a YMC silica column (250 mm×10 mm, i.d., 5 μm) by eluting with mixtures of *n*-hexane and EtOAc or *n*-hexane and isopropyl alcohol at 4.0 mL/min, respectively. Analytical TLC was carried out on pre-coated silica gel GF_254_ plates (Qingdao Marine Chemical Industry, Qingdao, China), and spots were visualized under UV light or by spraying with 5% H_2_SO_4_ in EtOH followed by heating at 120 °C.

### 2.2 Cytotoxicity bioassay

The cytotoxicity of the compounds against the human cancer cell lines (HepG2, Daoy, NCI-H1650, HCT116, and BGC823) was measured using the MTT assay [2]. Briefly, the cells were maintained in an RRMI S7 1640 medium supplemented with 10% (v/v) fetal bovine serum (FBS), 100 units/mL penicillin, and 100 μg/mL streptomycin. Cultures were incubated at 37 °C in a humidified atmosphere of 5% CO_2_. Tumor cells were seeded in 96-well microtiter plates at 1200 cells/well. After 24 h, compounds were added to the wells. After incubation for 96 h, cell viability was determined by measuring the metabolic conversion of 3-(4,5-dimethylthiazol-2-yl)-2,5-diphenyltetrazolium bromide (MTT) into purple formazan crystals by viable cells. The MTT assay results were read using an ELISA reader (Bio-Rad, USA) at 570 nm. All compounds were tested at five concentrations (10^−5^, 10^−6^, 10^−7^, 10^−8^, and 10^−9^ M) in 100% DMSO with a final concentration of DMSO of 0.1% (v/v) in each well. Paclitaxel was used as a positive control. Each concentration of the compounds was tested in three parallel experiments. IC_50_ values were calculated using Microsoft Excel software.

### 2.3 HIV-inhibitory bioassay

293 T cells (2×10^5^) were co-transfected with 0.6 μg of pNL–Luv-E^−^–Vpu^−^ and 0.4 μg of pHIT/G. After 48 h, the VSV-G pseudotyped viral supernatant (HIV-1) was harvested by filtration through a 0.45 μm filter and the concentration of viral capsid protein was determined by p24 antigen capture ELISA (Biomerieux). SupT1 cells were exposed to VSV-G pseudotyped HIV-1 (MOI=1) at 37 °C for 48 h in the absence or presence of test compounds (efavirenz was used as positive control). The inhibition rate was determined by using a firefly luciferase assay system (Promega) [3].
