# Supplementary material for: Data on eleven sesquiterpenoids from the cultured mycelia of Ganoderma capense
Source: Data Brief. 2017 Apr 19;12:361–3. doi: 10.1016/j.dib.2017.04.012 (PMC5413196; doi:10.1016/j.dib.2017.04.012)
Supplement: Supplementary file 2 — Supplementary material [file mmc2.pdf]

Figure S1. ^{1}H NMR spectrum of compound **1** in $CDCl_3$

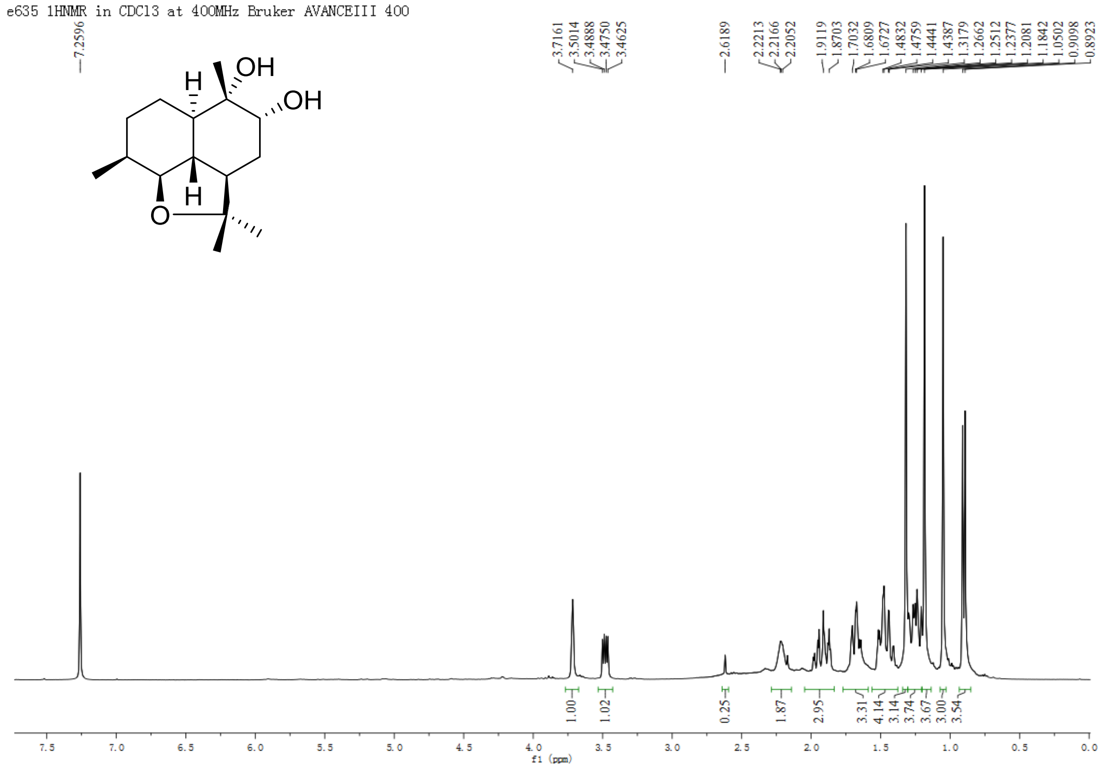

Figure S2. ^{13}C NMR spectrum of compound **1** in $CDCl_3$

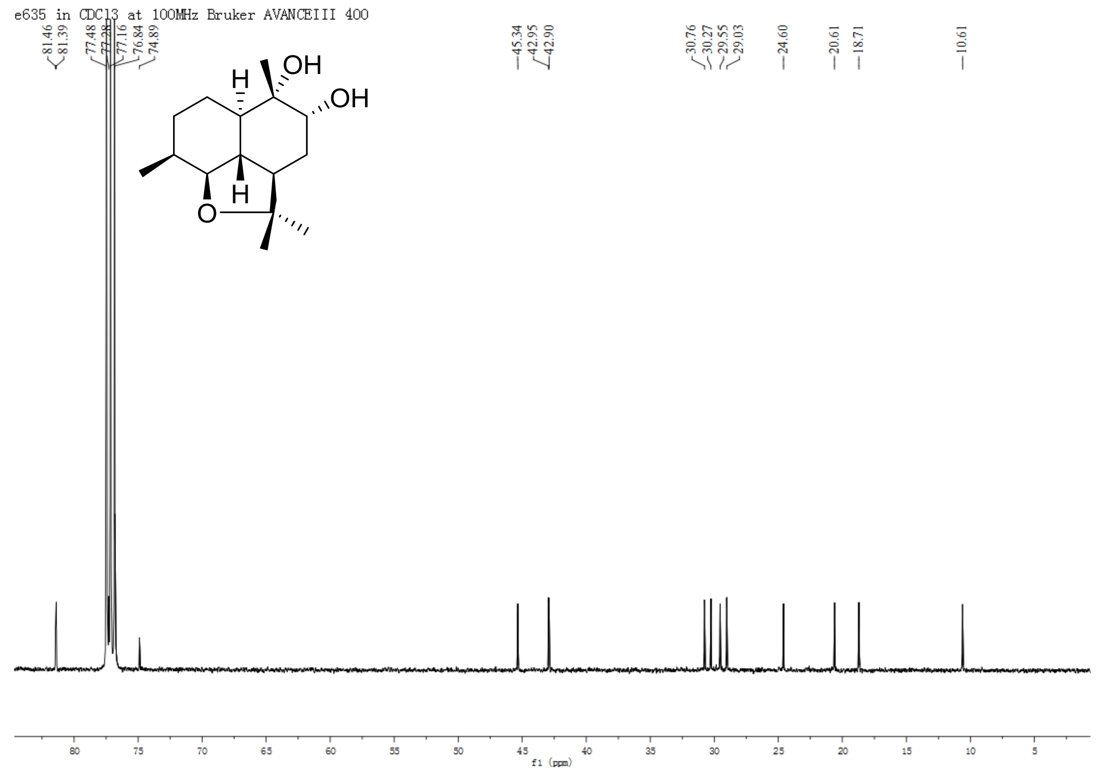

Figure S2-2. Enlarge ^{13}C NMR spectrum of compound **1** in $CDCl_3$

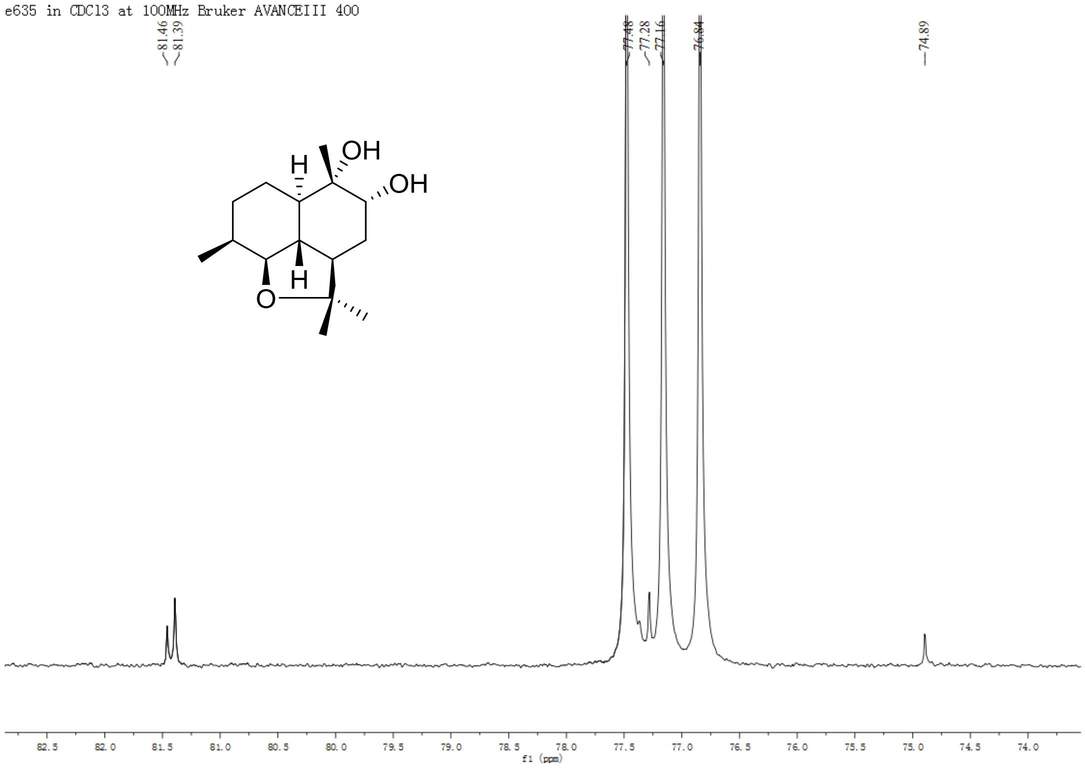

Figure S3. DEPT spectrum of compound **1** in $CDCl_3$

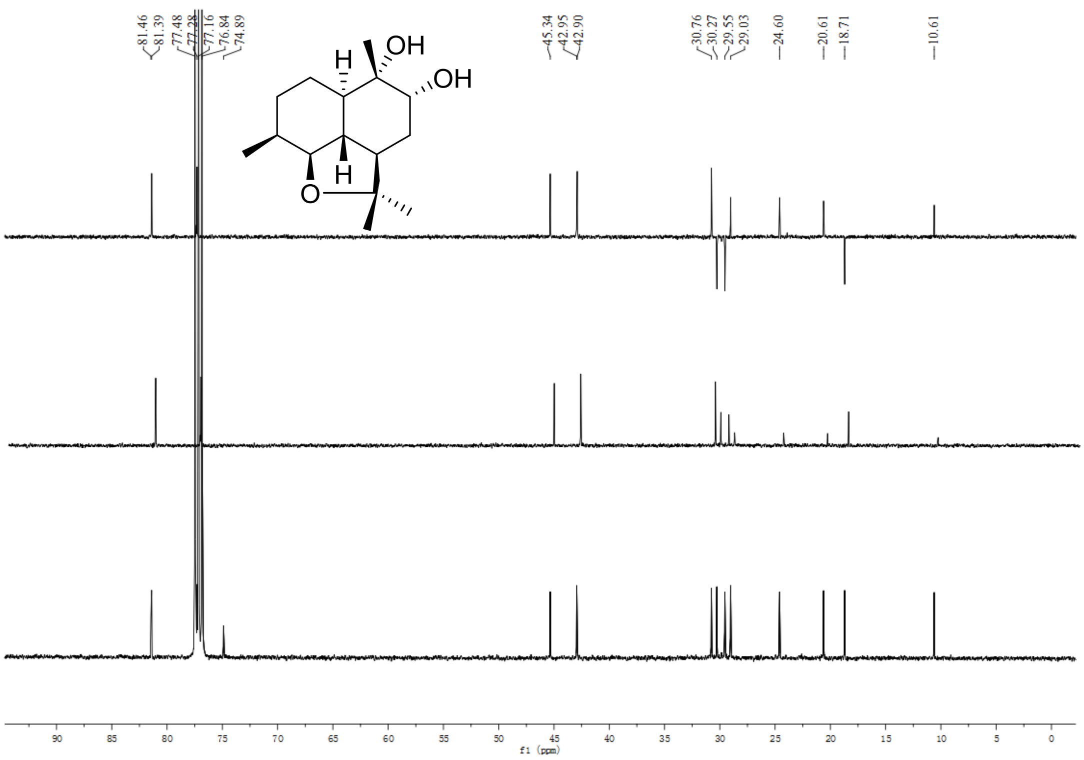

Figure S4. HSQC spectrum of compound **1** in $CDCl_3$

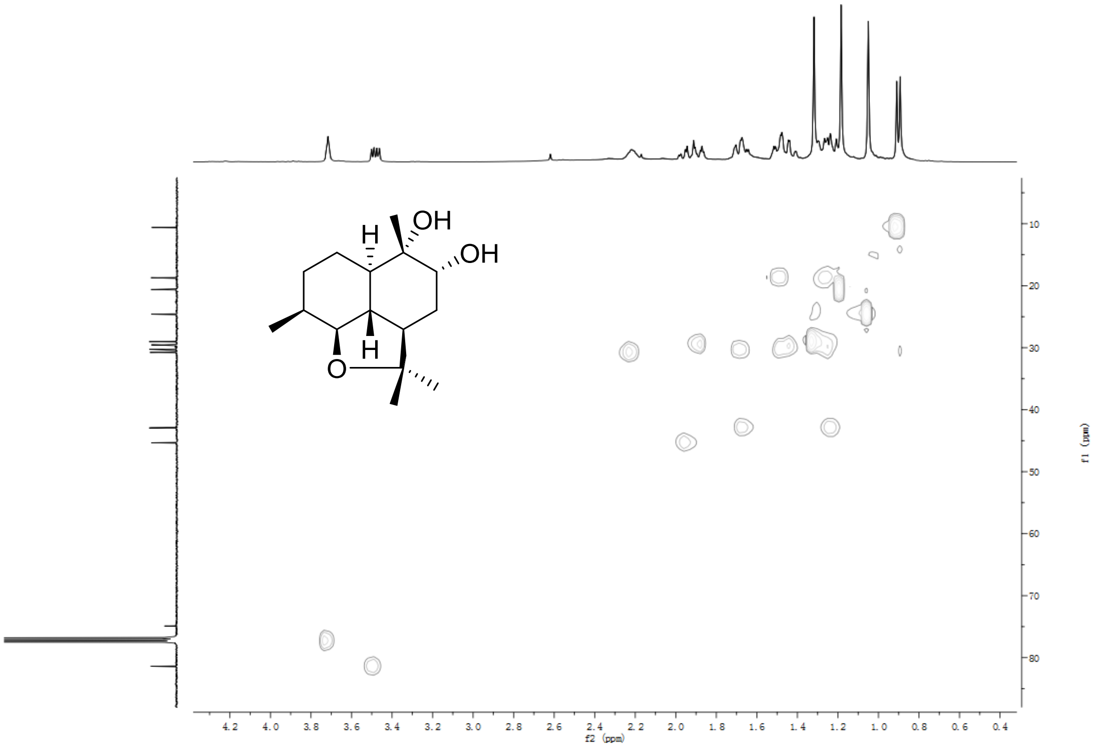

Figure S5. ^{1}H-^{1}H COSY spectrum of compound **1** in $CDCl_3$

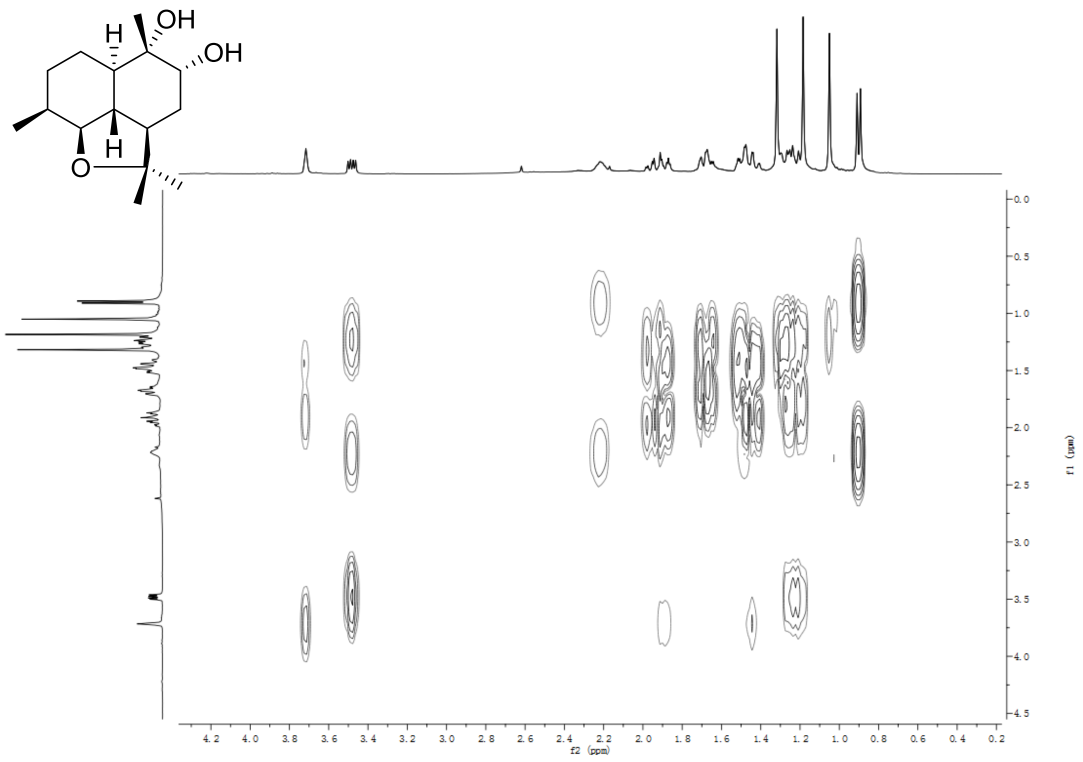

Figure S6. HMBC spectrum of compound **1** in $CDCl_3$

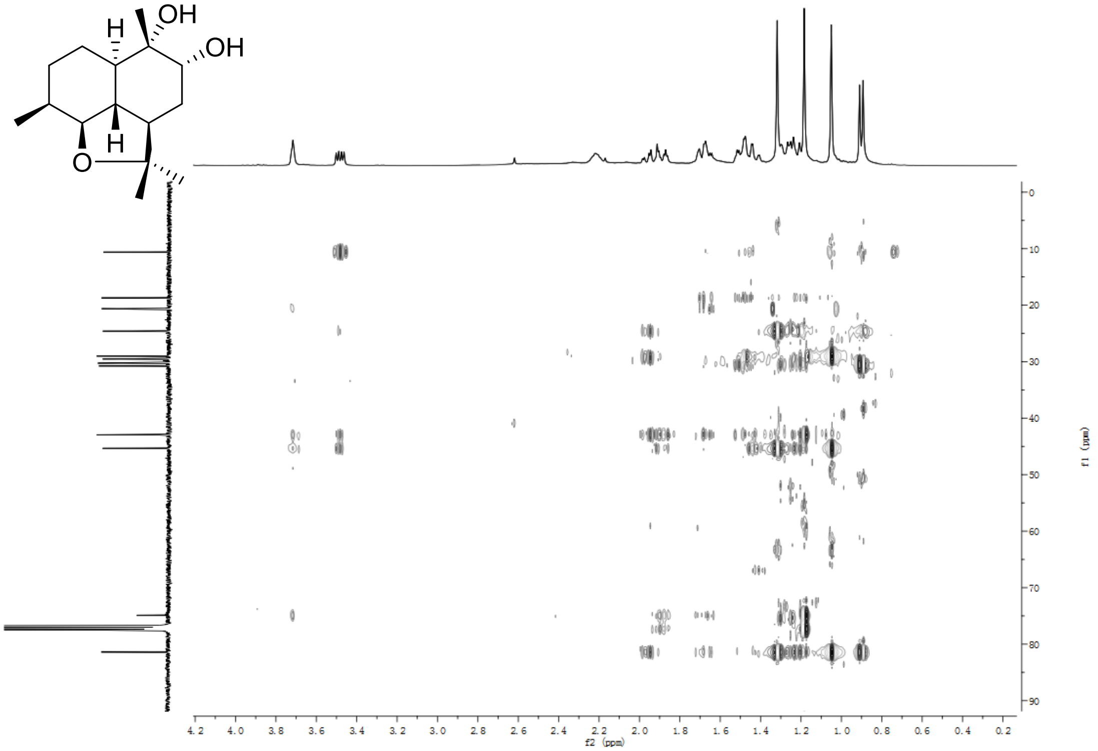

Figure S7. NOESY spectrum of compound **1** in $CDCl_3$

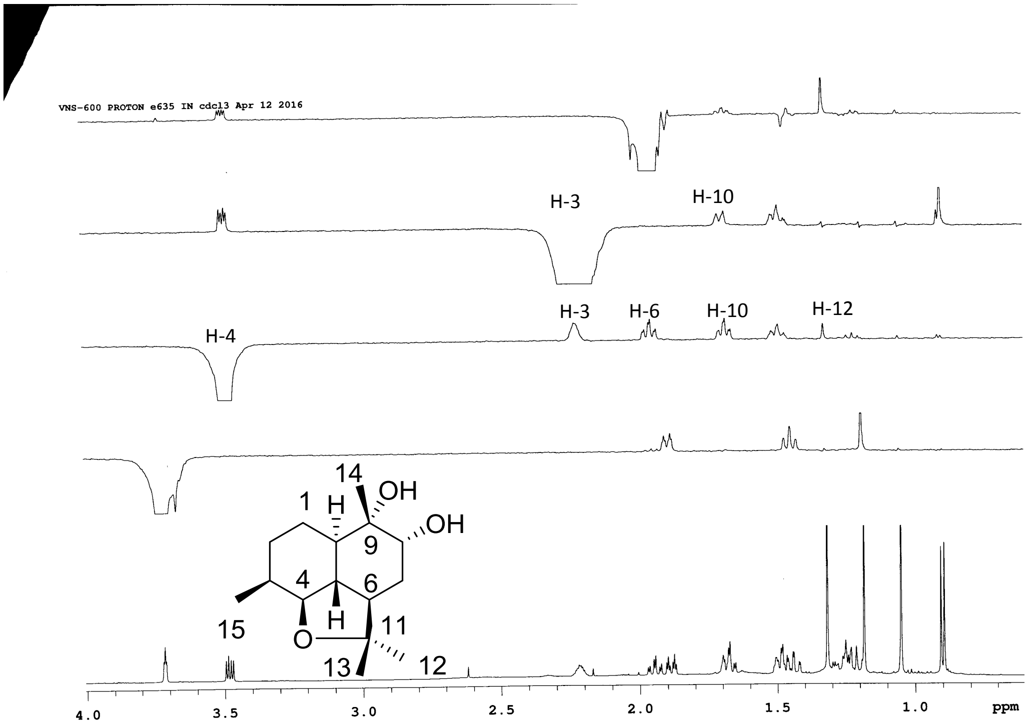

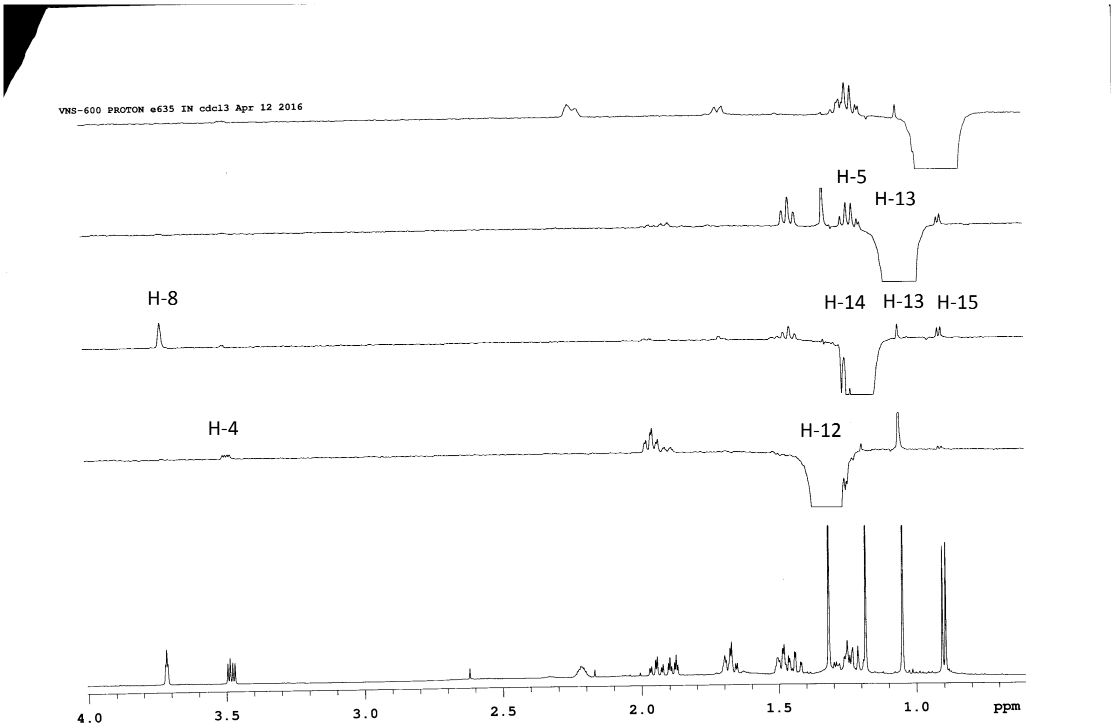

Figure S8. HRESIMS spectrum of compound **1**

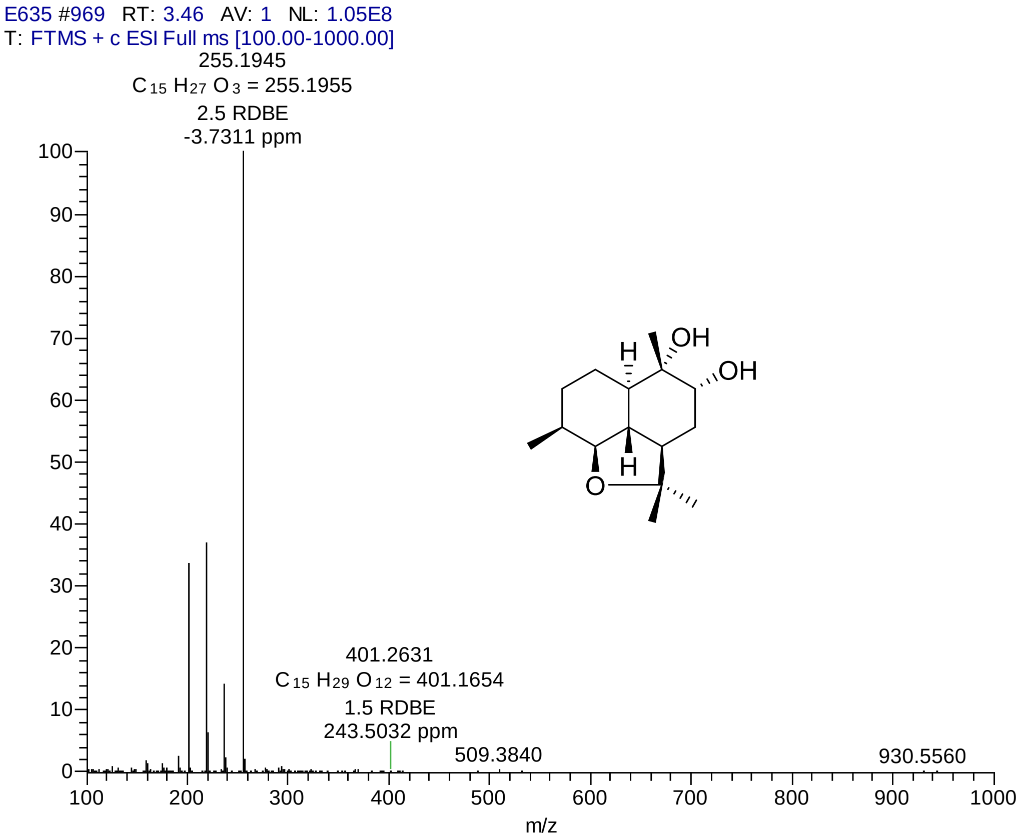

Figure S9. IR spectrum of compound **1**

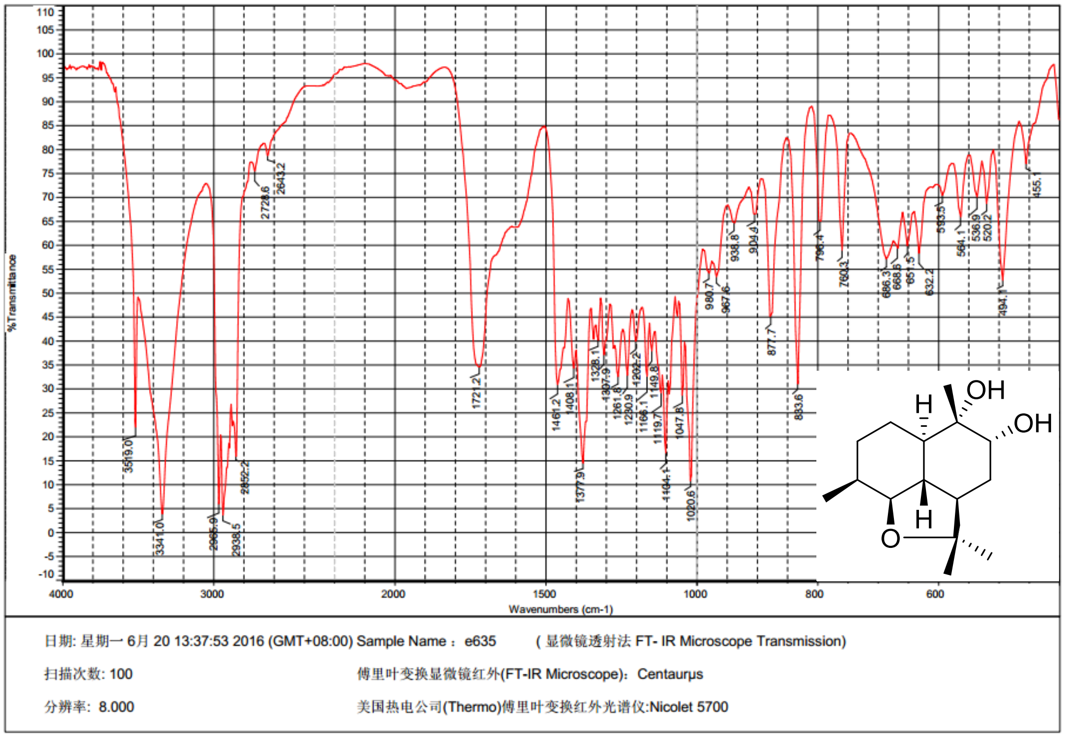

Figure S10. $Mo_2(AcO)_4$-induced CD spectrum of compound **1**

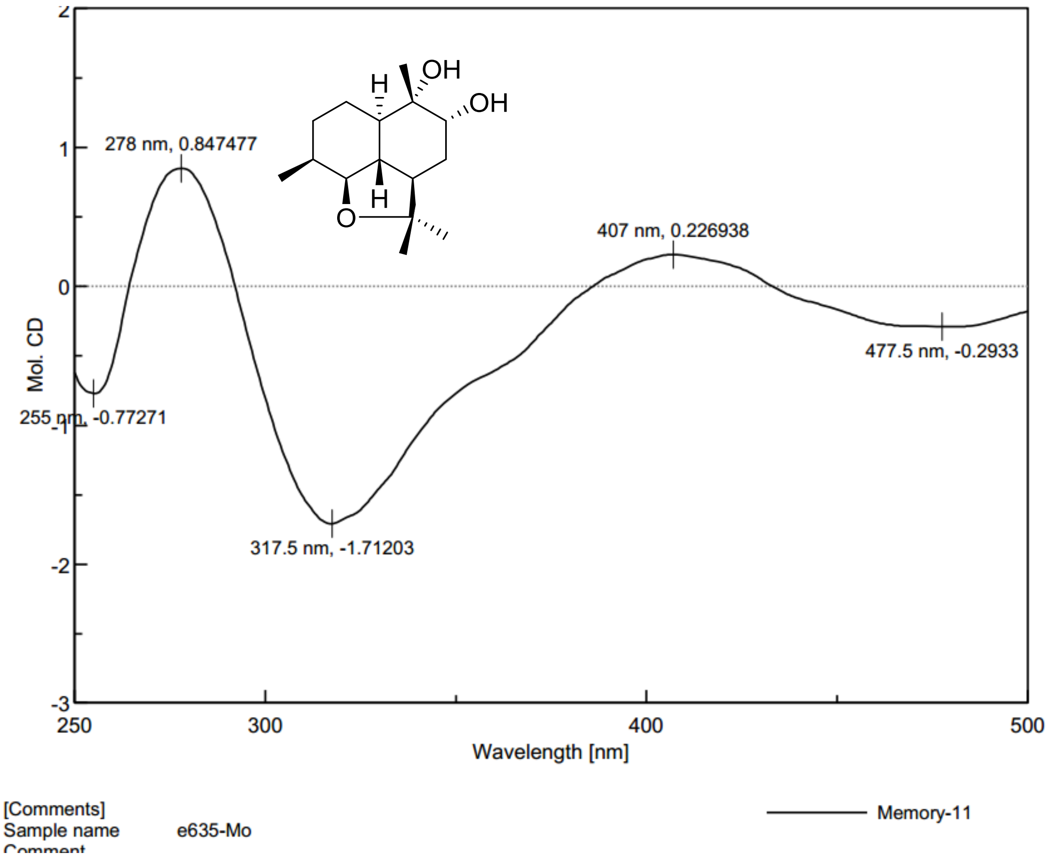

Figure S11. ^{1}H NMR spectrum of compound **2** in $CDCl_3$

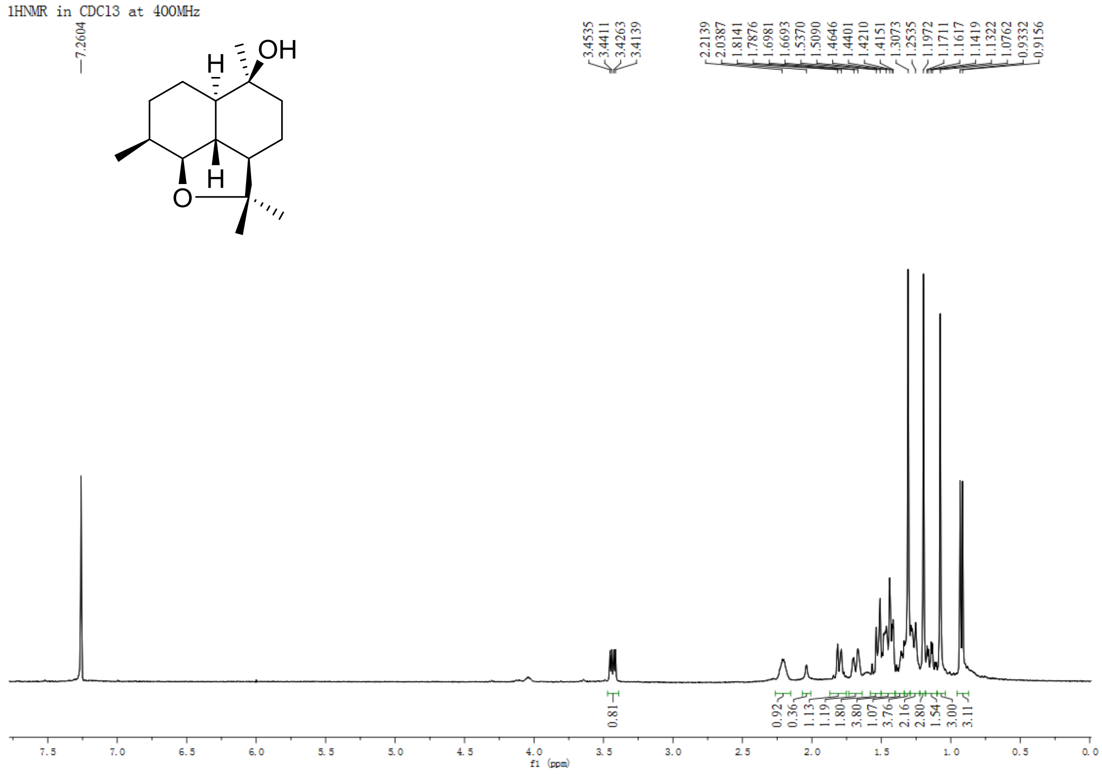

Figure S12. ^{13}C NMR spectrum of compound **2** in $CDCl_3$

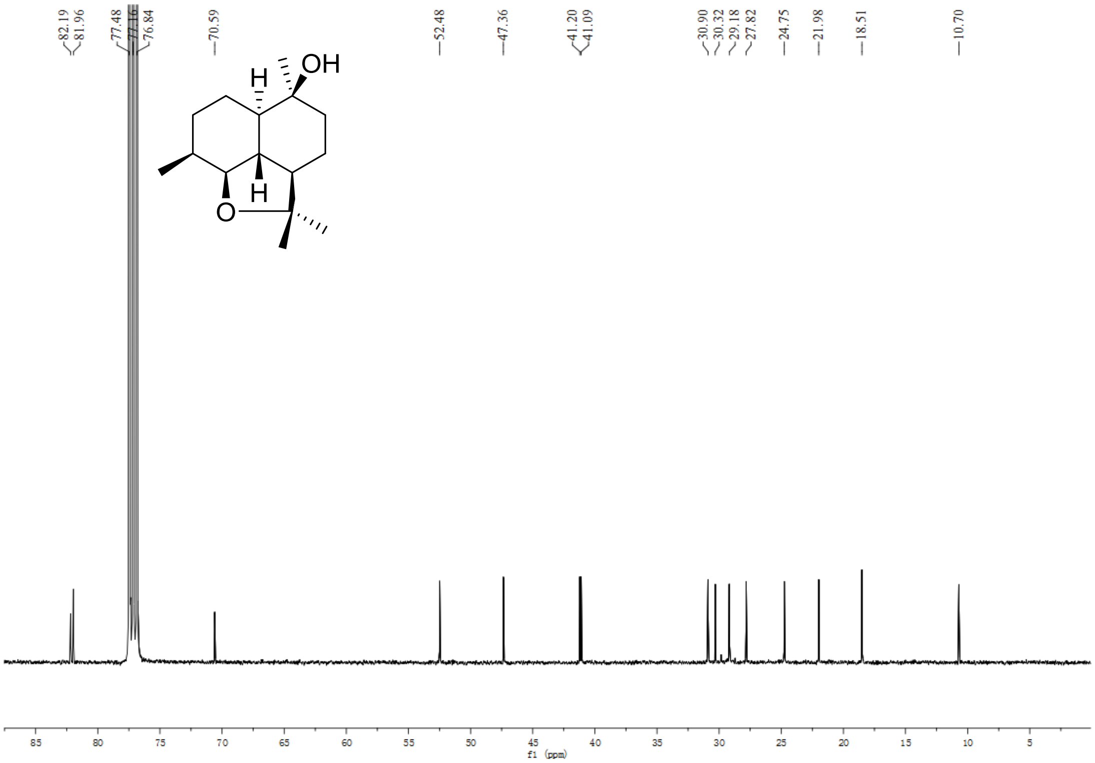

Figure S13. DEPT spectrum of compound **2** in $CDCl_3$

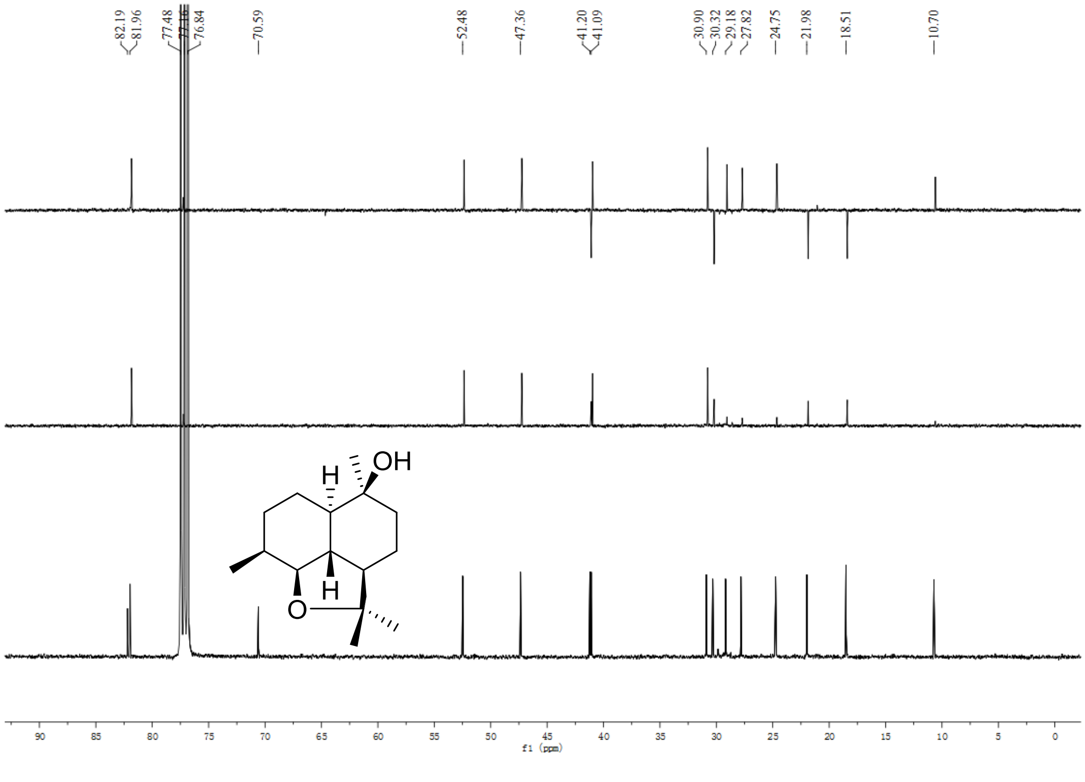

Figure S14. HSQC spectrum of compound **2** in $CDCl_3$

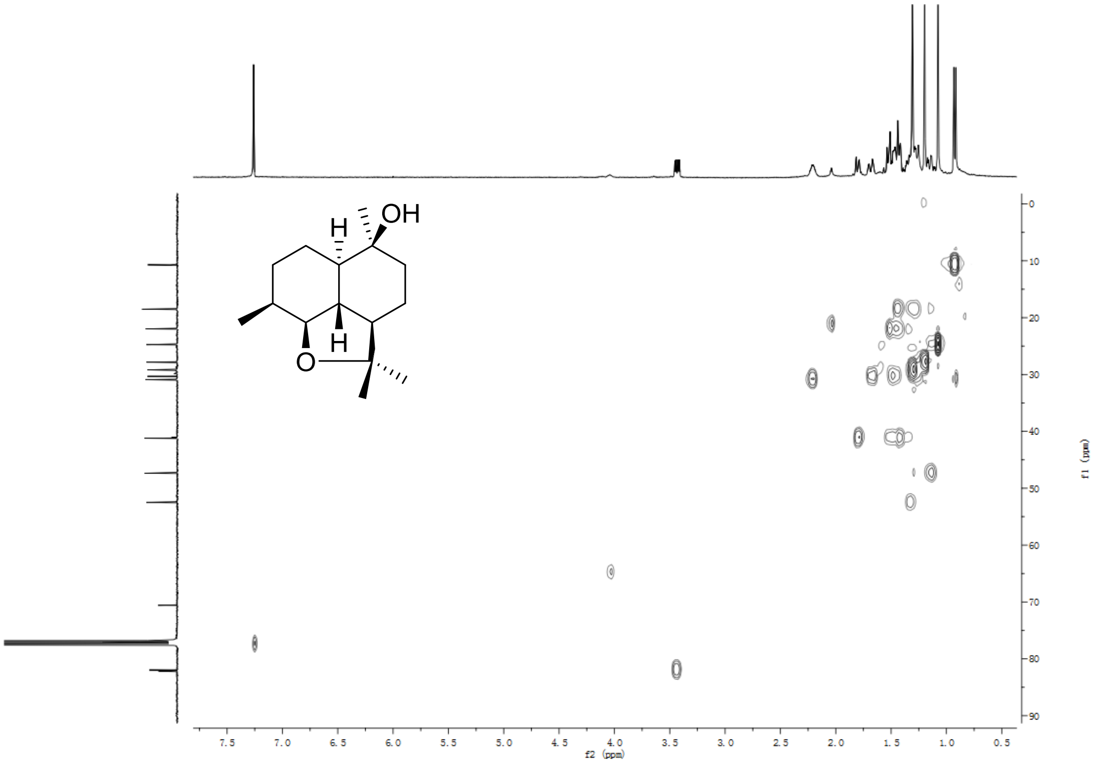

Figure S15. ^{1}H-^{1}H COSY spectrum of compound **2** in $CDCl_3$

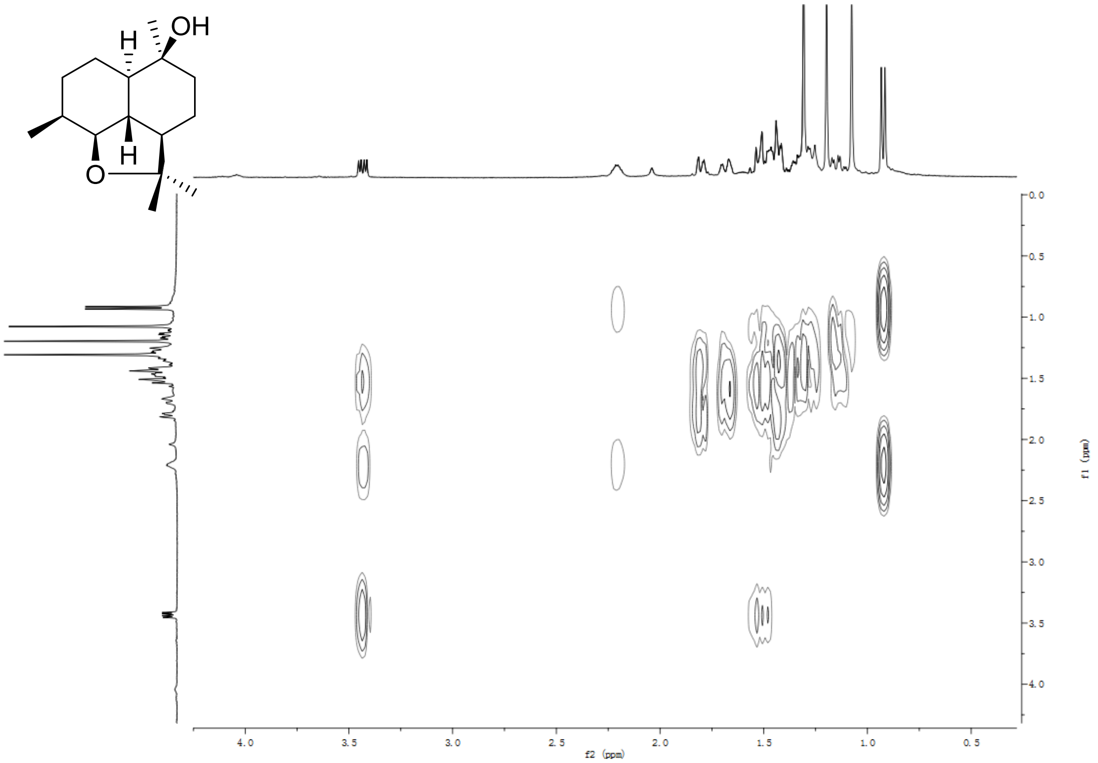

Figure S16. HMBC spectrum of compound **2** in $CDCl_3$

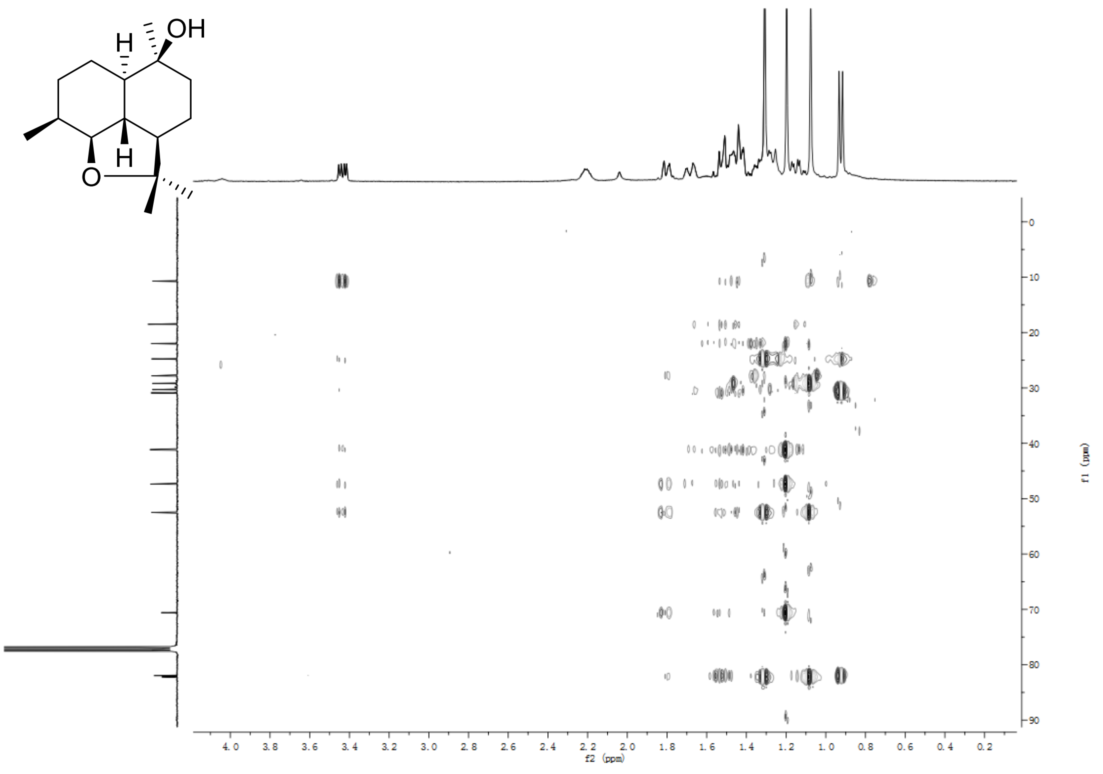

Figure S17. NOESY spectrum of compound **2** in $CDCl_3$

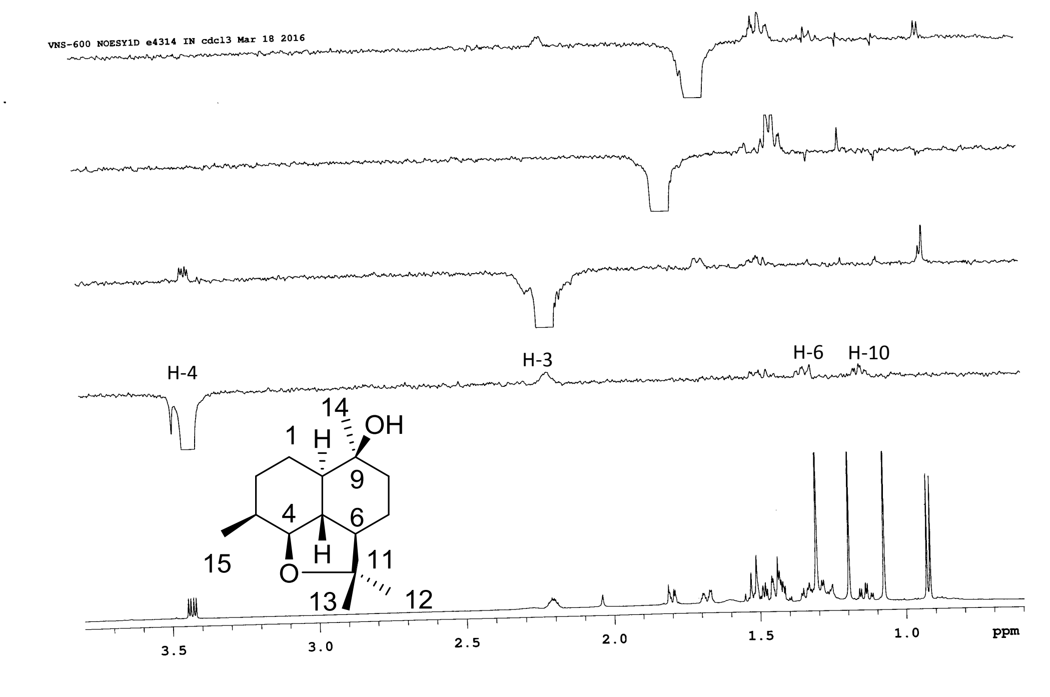

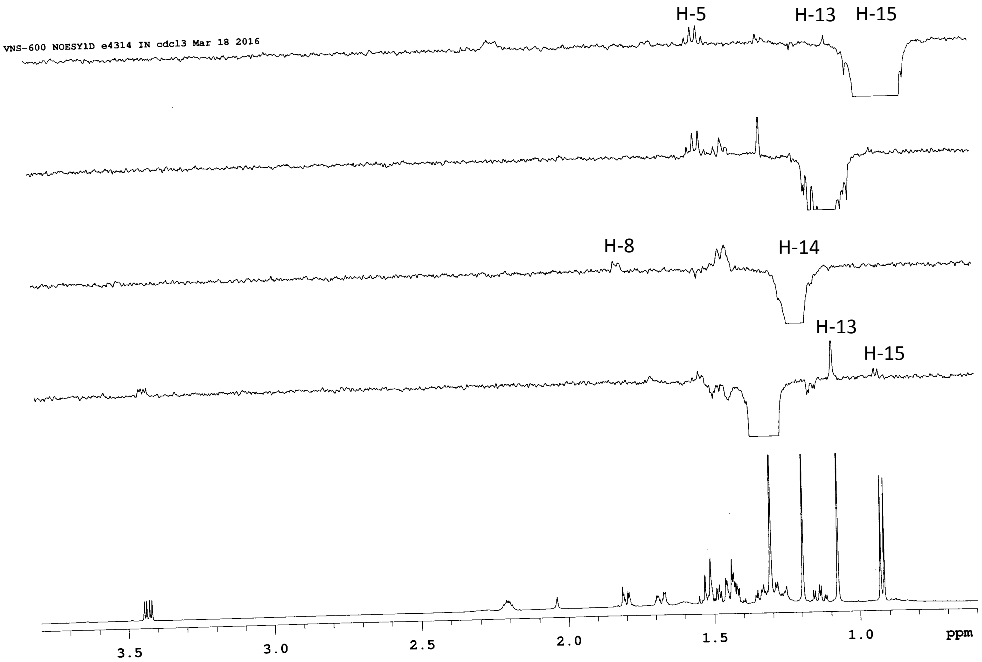

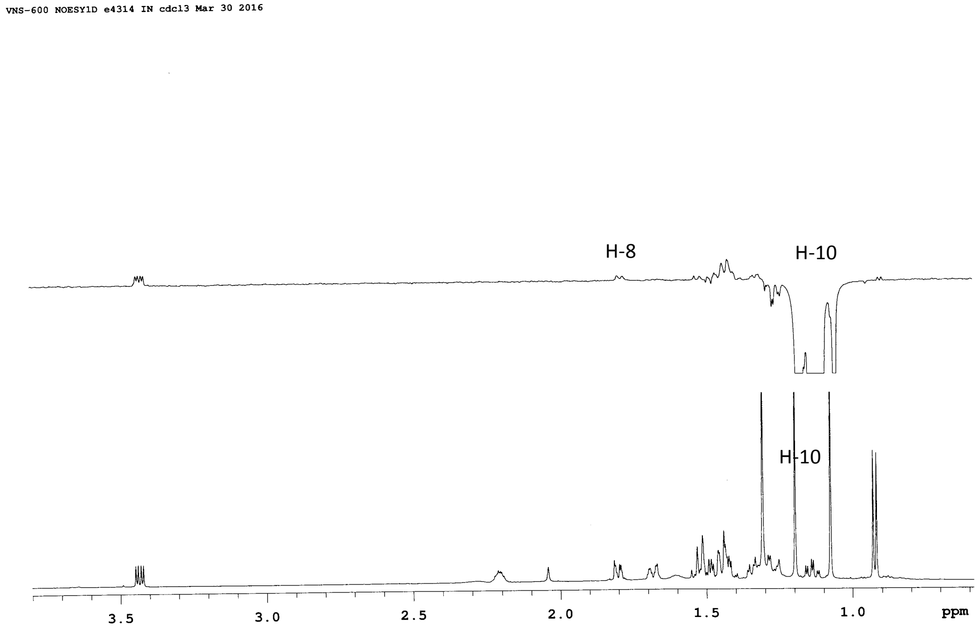

Figure S18. HRESIMS spectrum of compound **2**

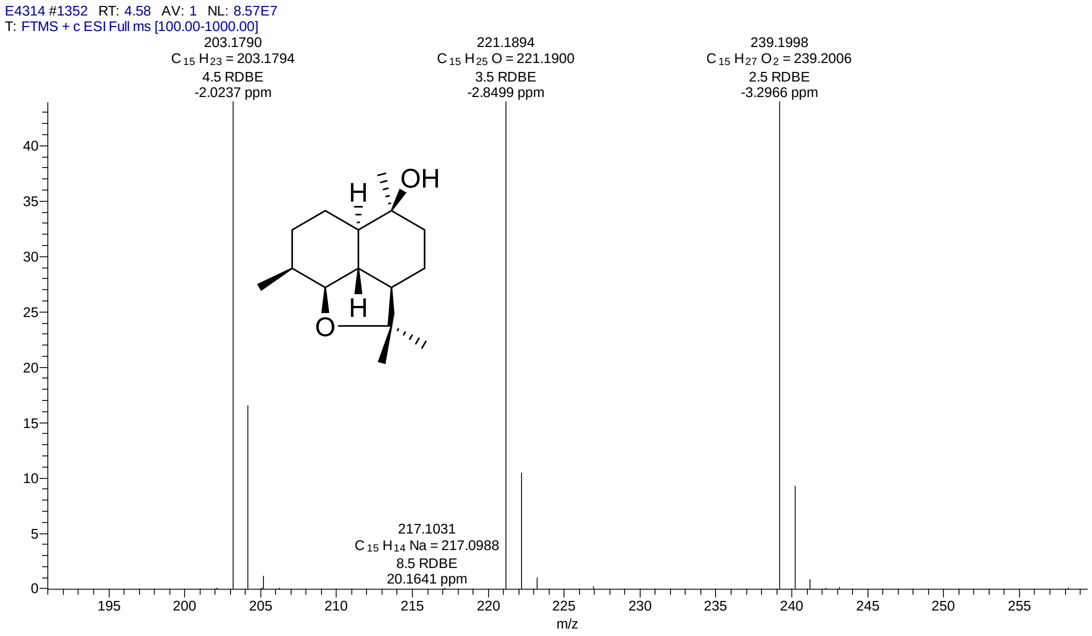

Figure S19. IR spectrum of compound **2**

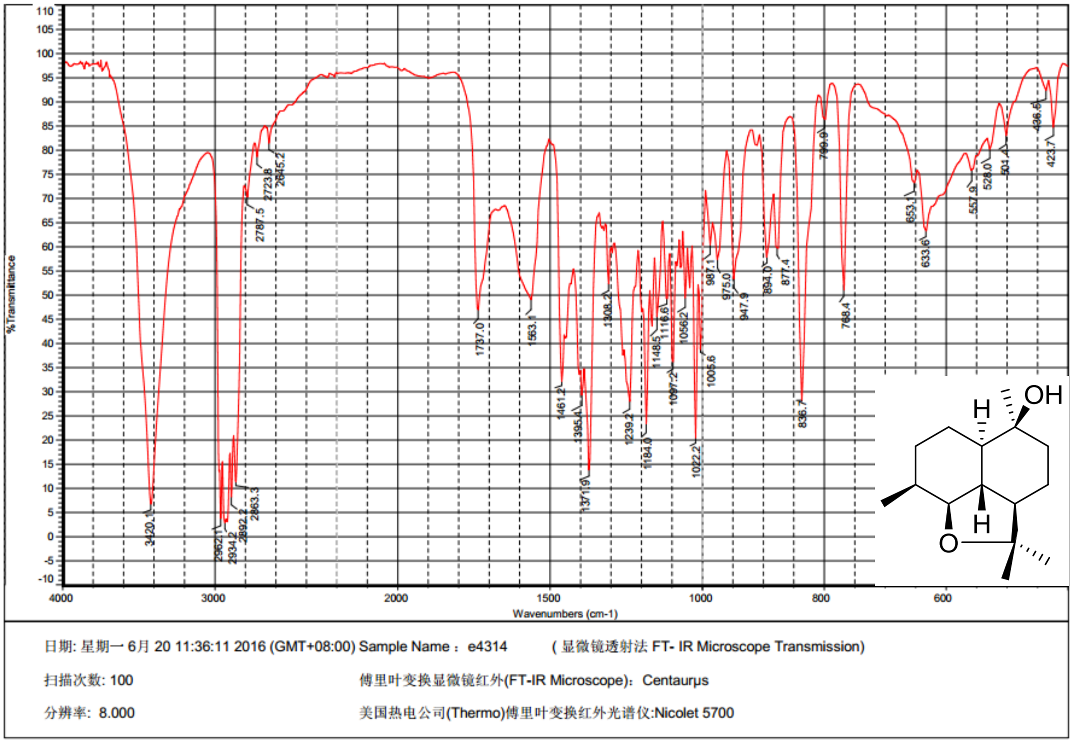

Figure S20. ^{1}H NMR spectrum of compound **3** in $CDCl_3$

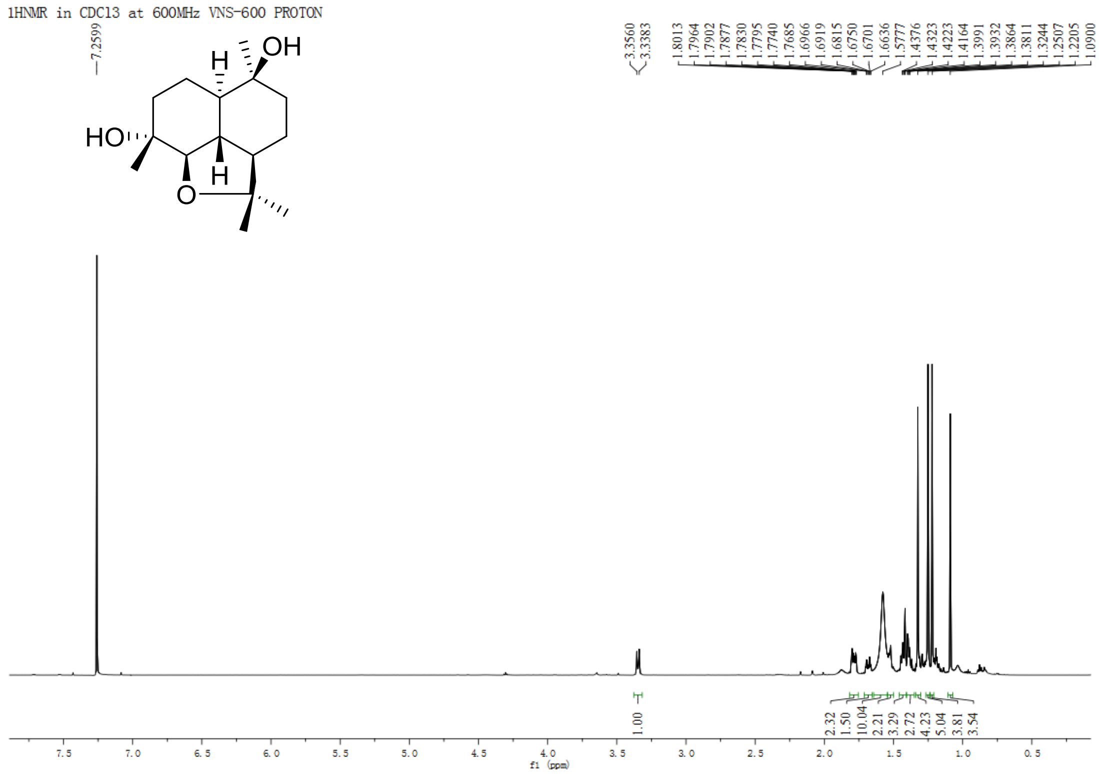

Figure S21. ^{13}C NMR spectrum of compound **3** in $CDCl_3$

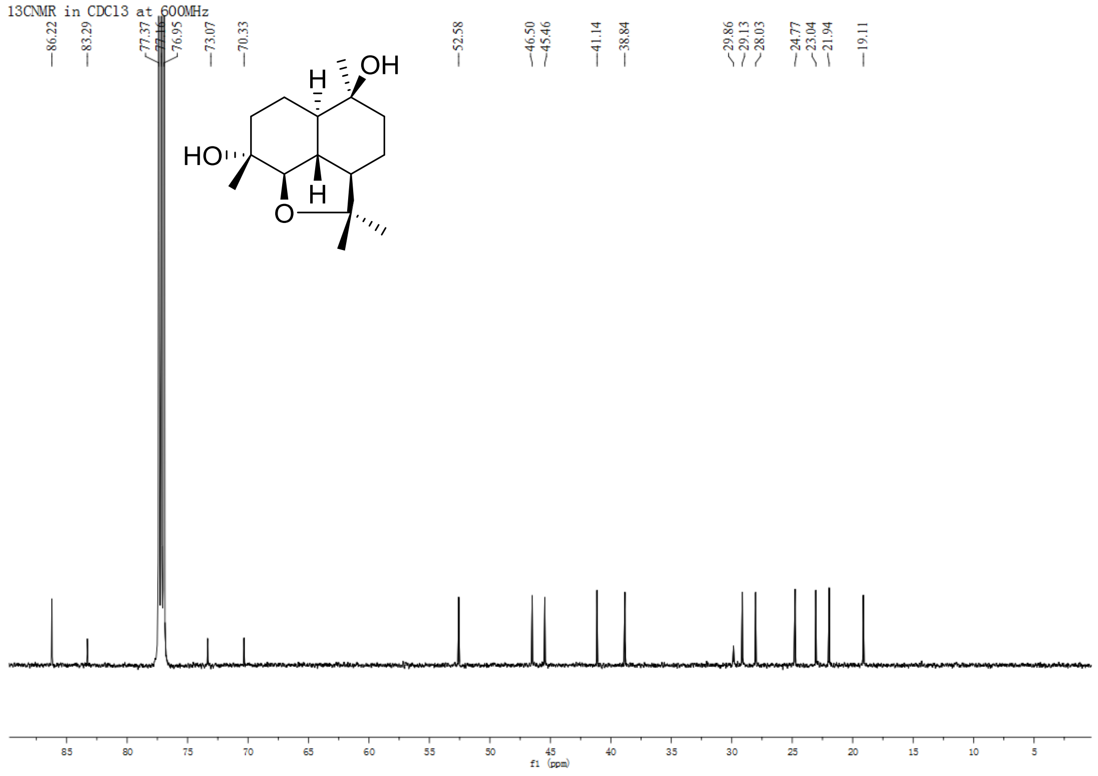

Figure S22. DEPT spectrum of compound **3** in $CDCl_3$

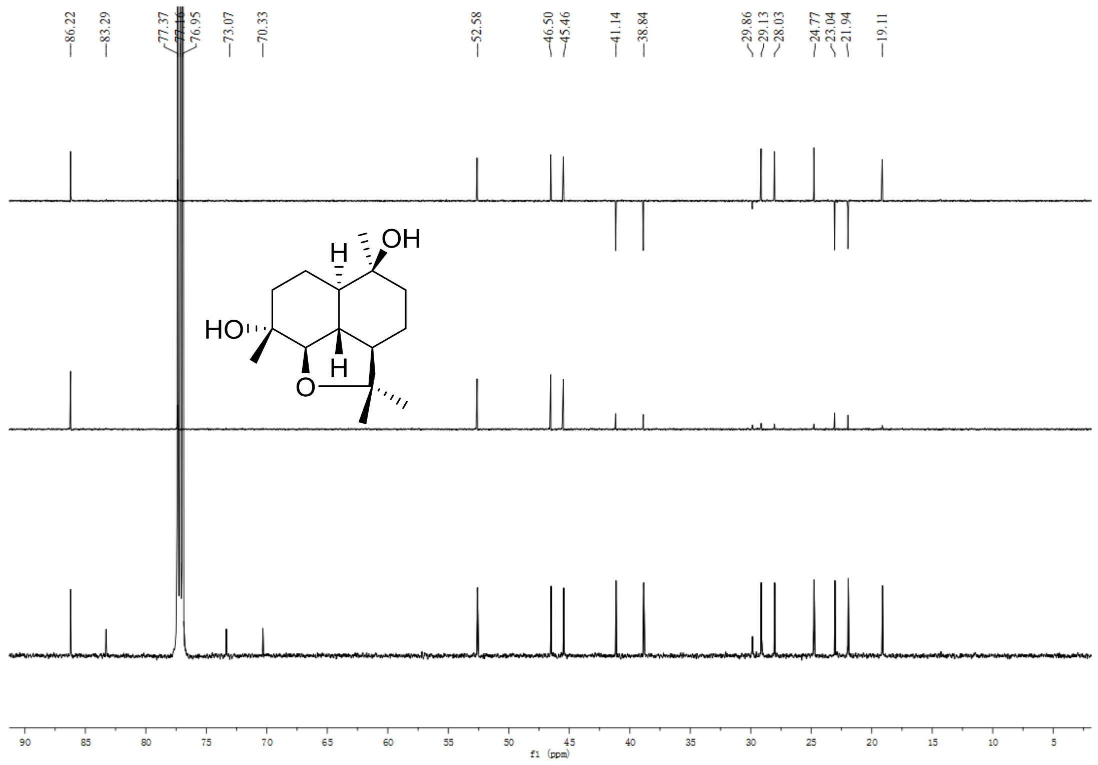

Figure S23. HSQC spectrum of compound **3** in $CDCl_3$

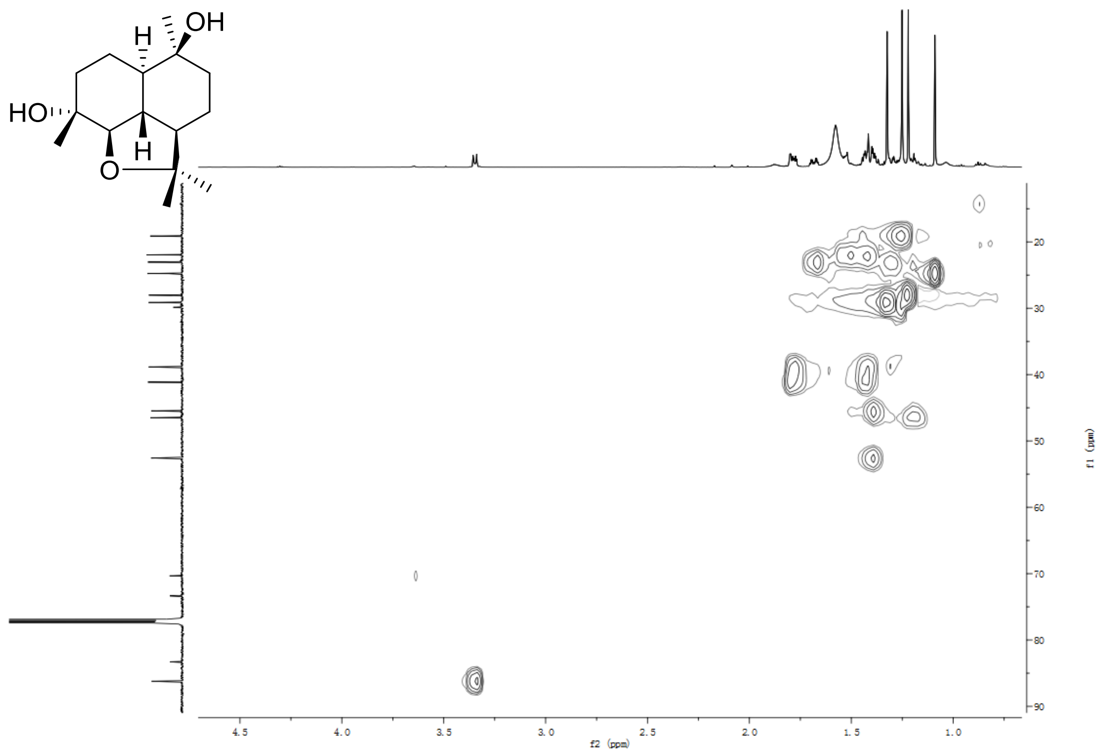

Figure S24. ^1H-^1H COSY spectrum of compound **3** in $CDCl_3$

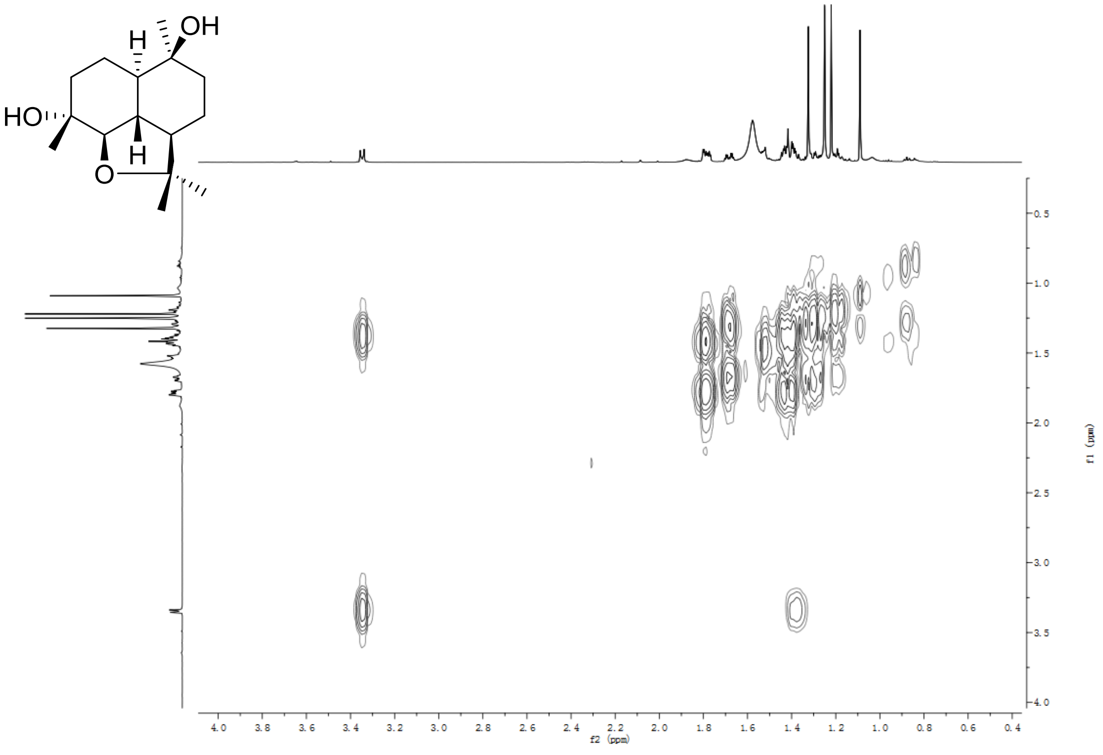

Figure S25. HMBC spectrum of compound **3** in $CDCl_3$

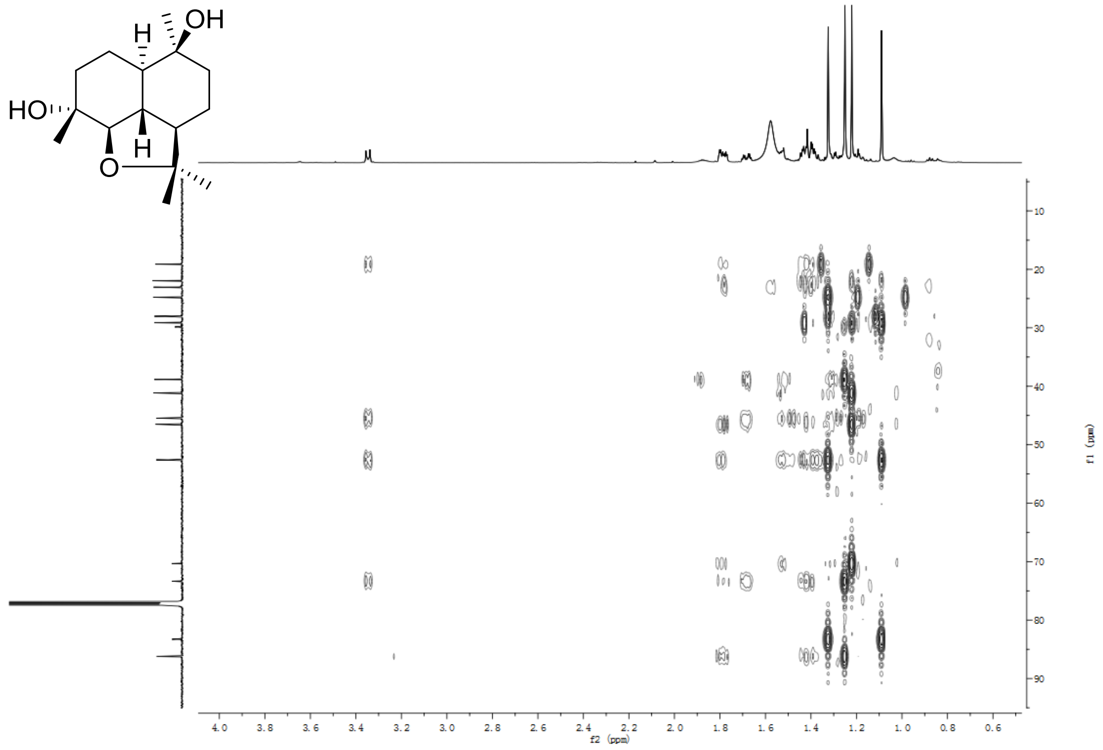

Figure S26. NOESY spectrum of compound **3** in $CDCl_3$

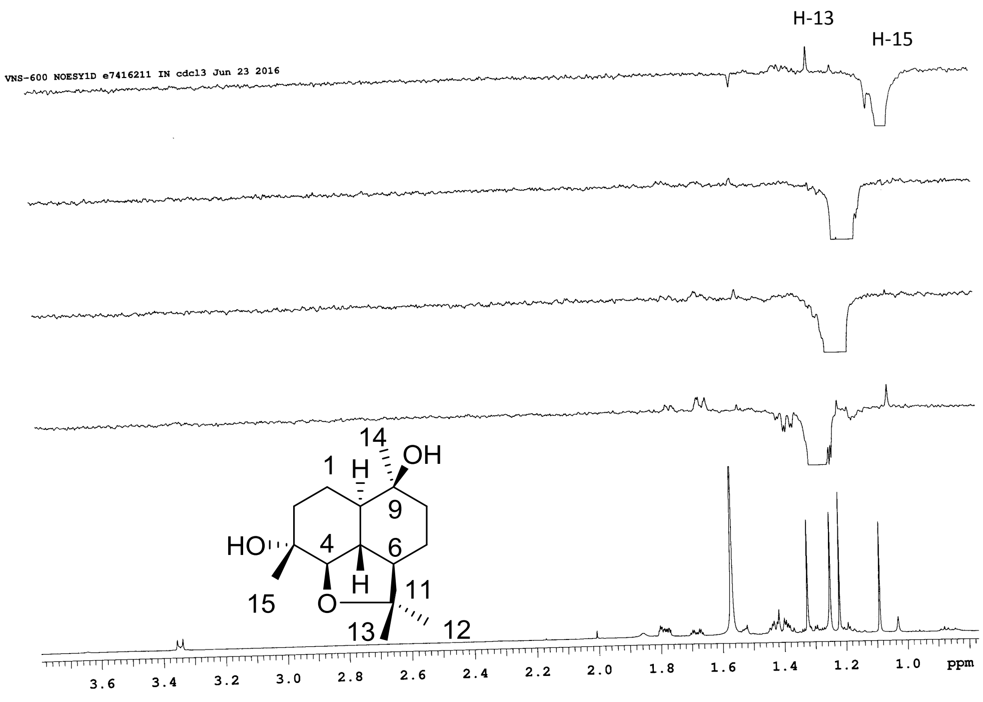

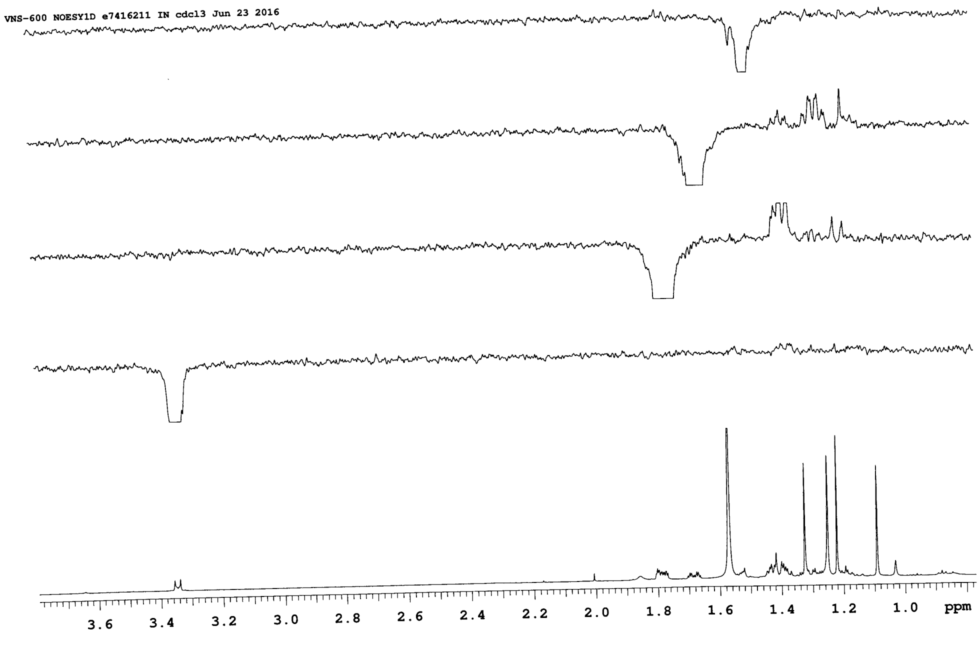

Figure S27. HRESIMS spectrum of compound **3**

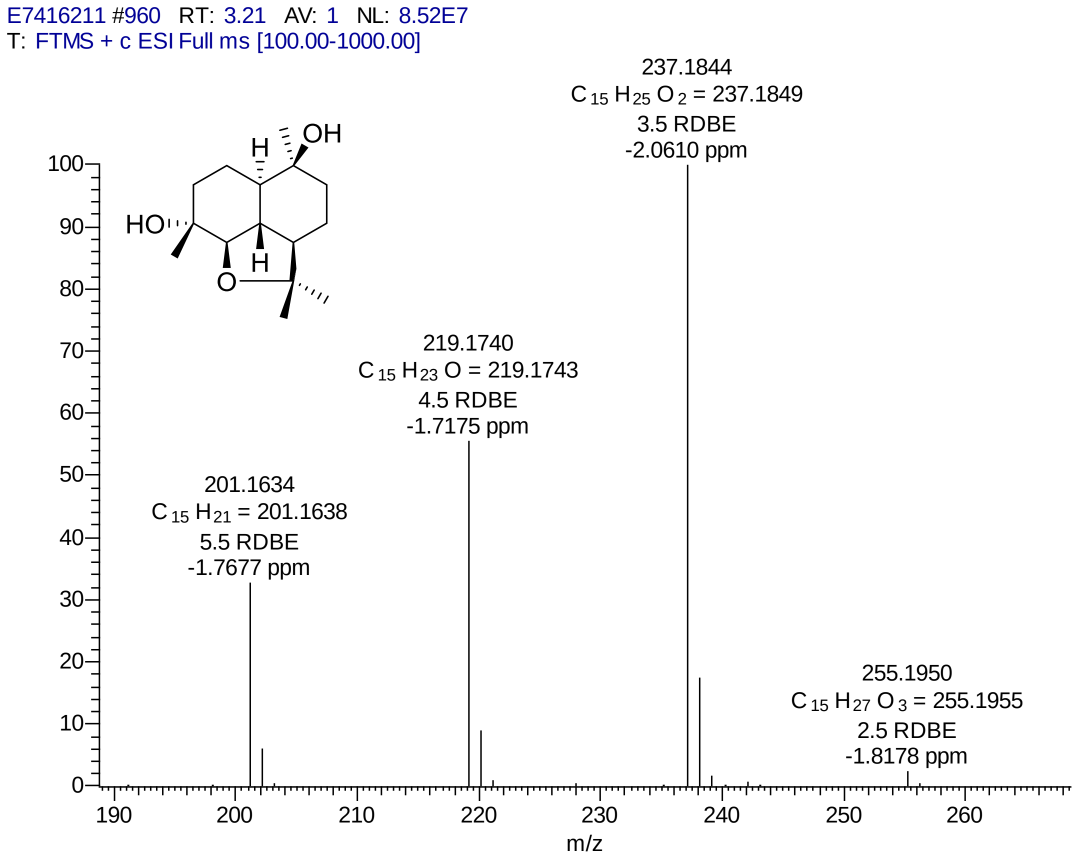

Figure S28. IR spectrum of compound **3**

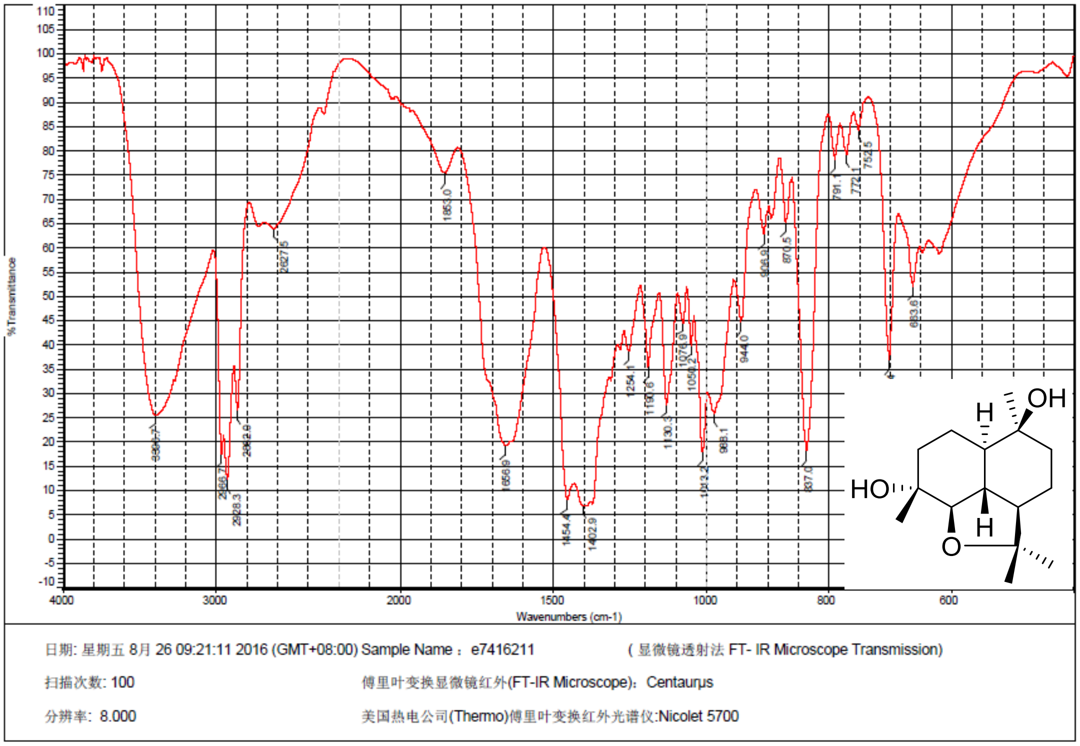

Figure S29. ^{1}H NMR spectrum of compound **4** in $CDCl_3$

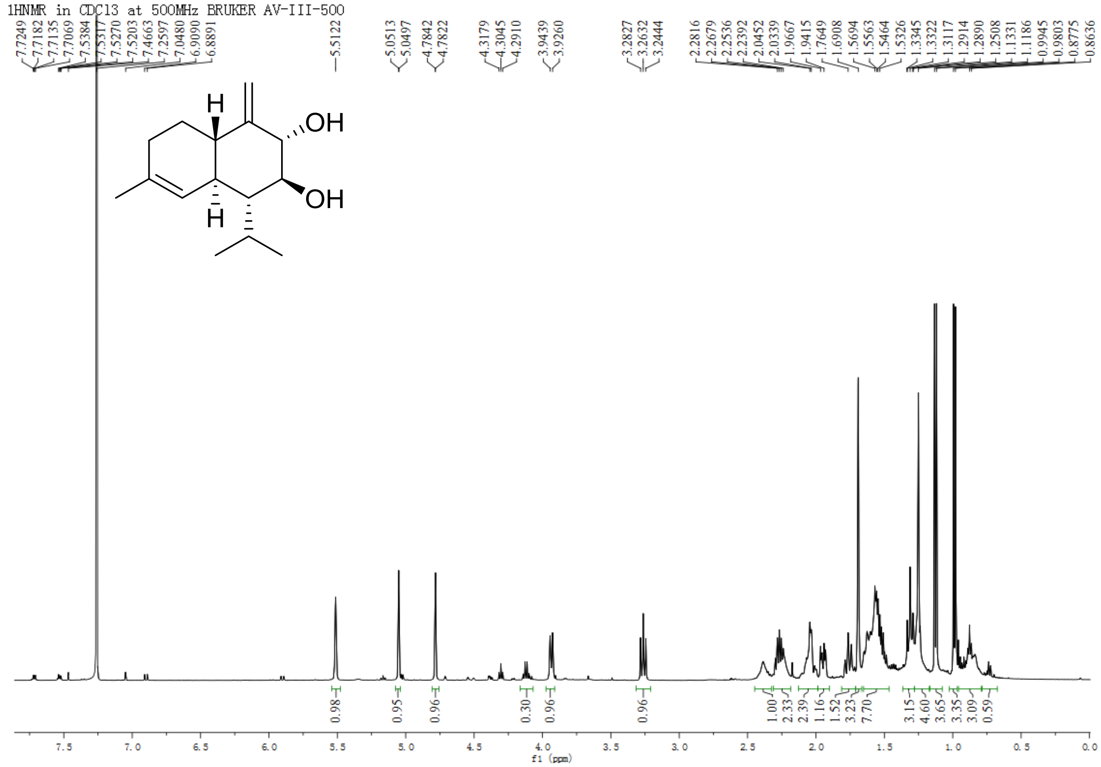

Figure S30. ^{13}C NMR spectrum of compound **4** in $CDCl_3$

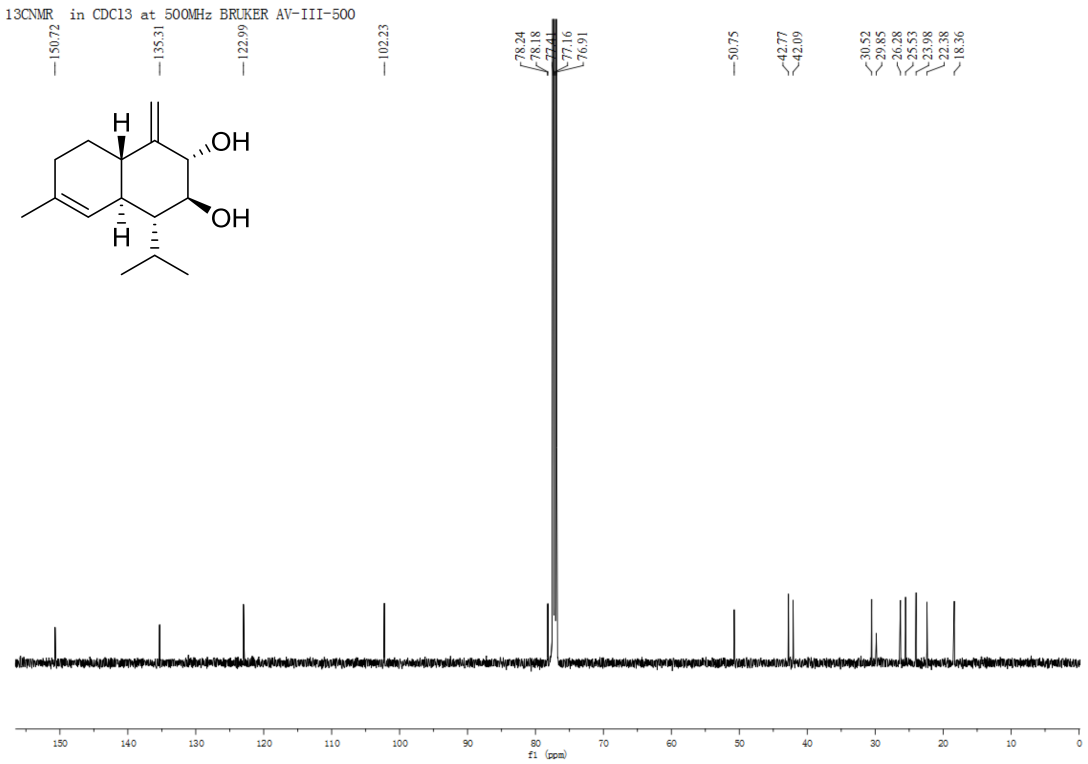

Figure S31. DEPT spectrum of compound **4** in $CDCl_3$

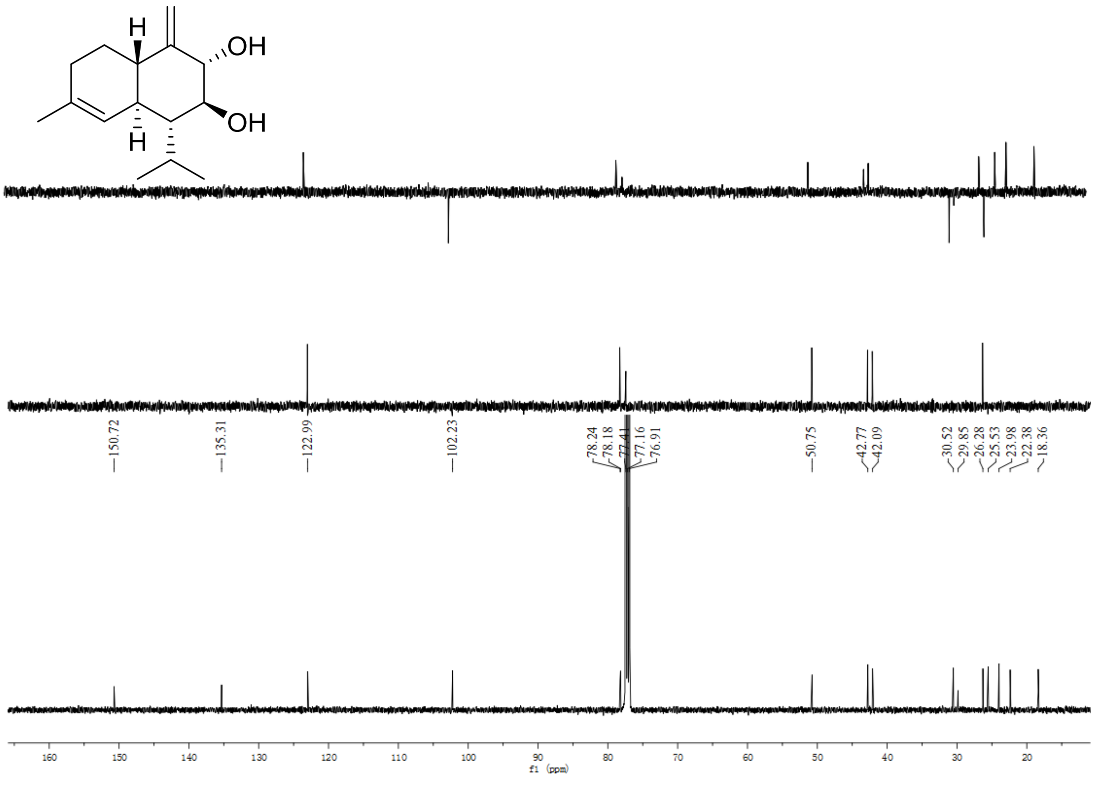

Figure S32. HSQC spectrum of compound **4** in $CDCl_3$

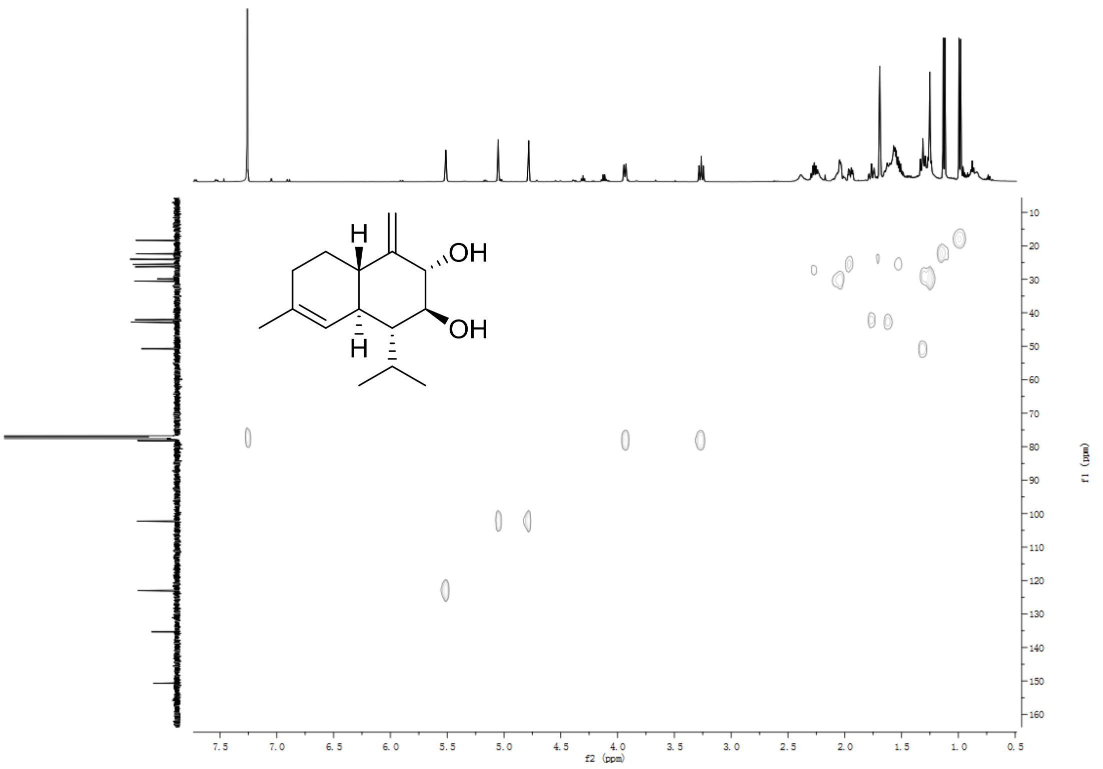

Figure S33. ^{1}H-^{1}H COSY spectrum of compound **4** in $CDCl_3$

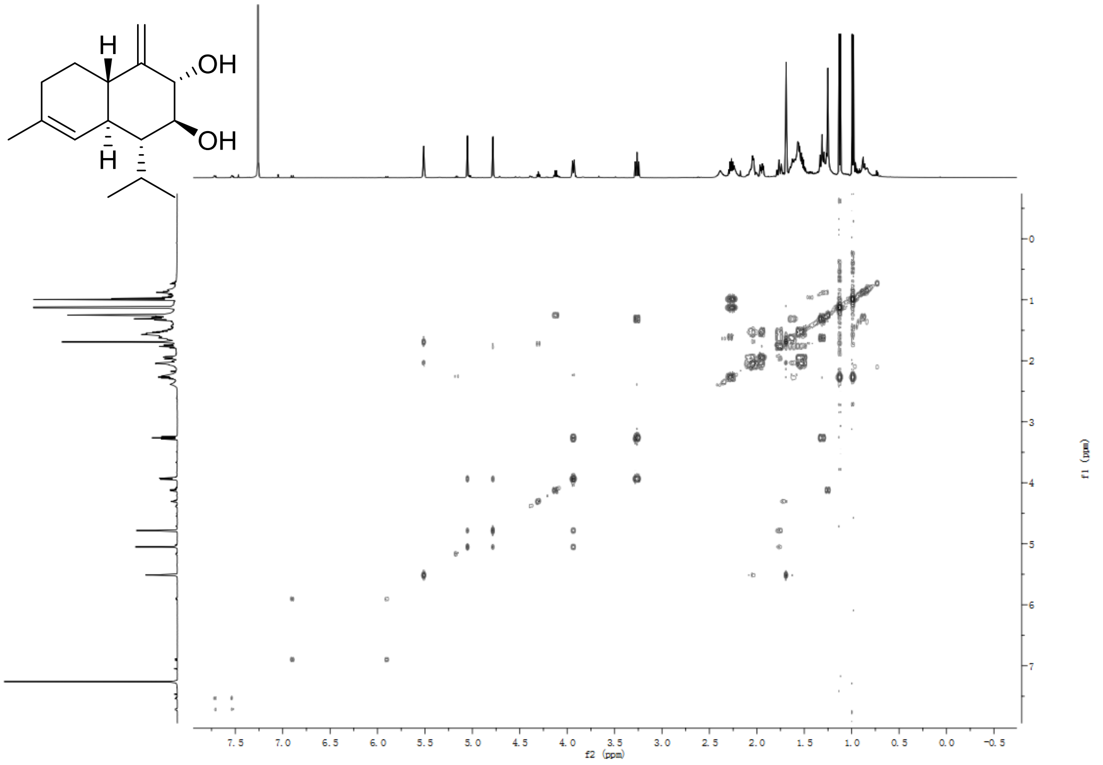

Figure S34. HMBC spectrum of compound **4** in $CDCl_3$

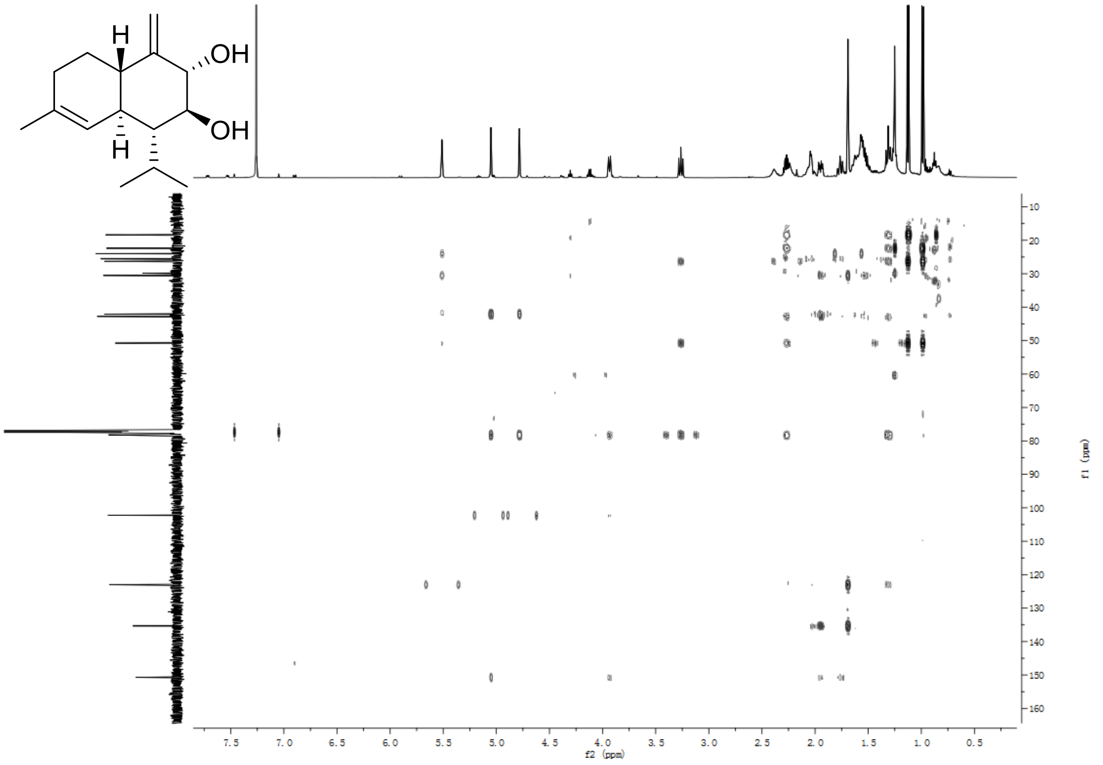

Figure S35. NOESY spectrum of compound **4** in $CDCl_3$

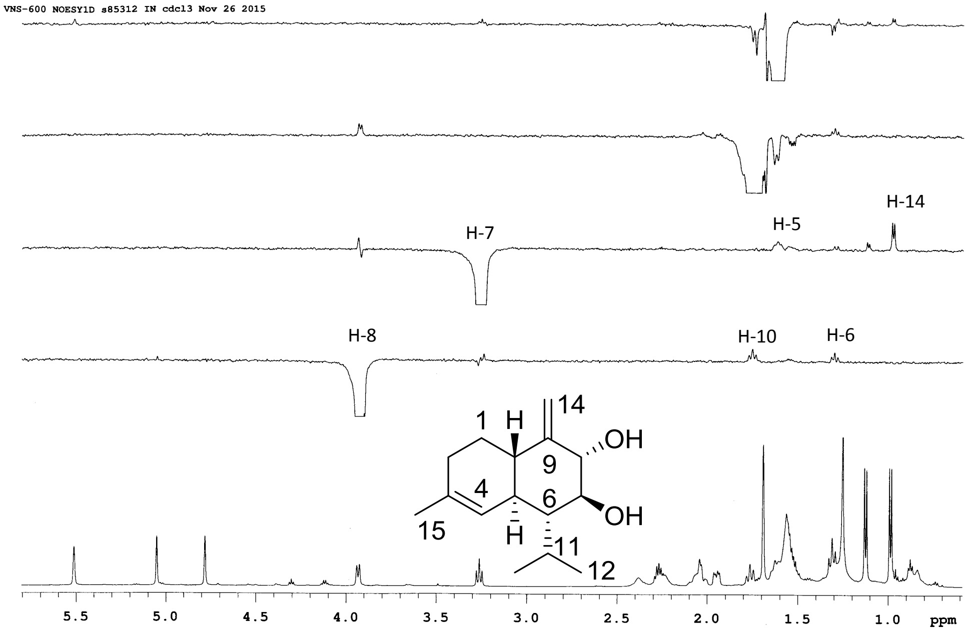

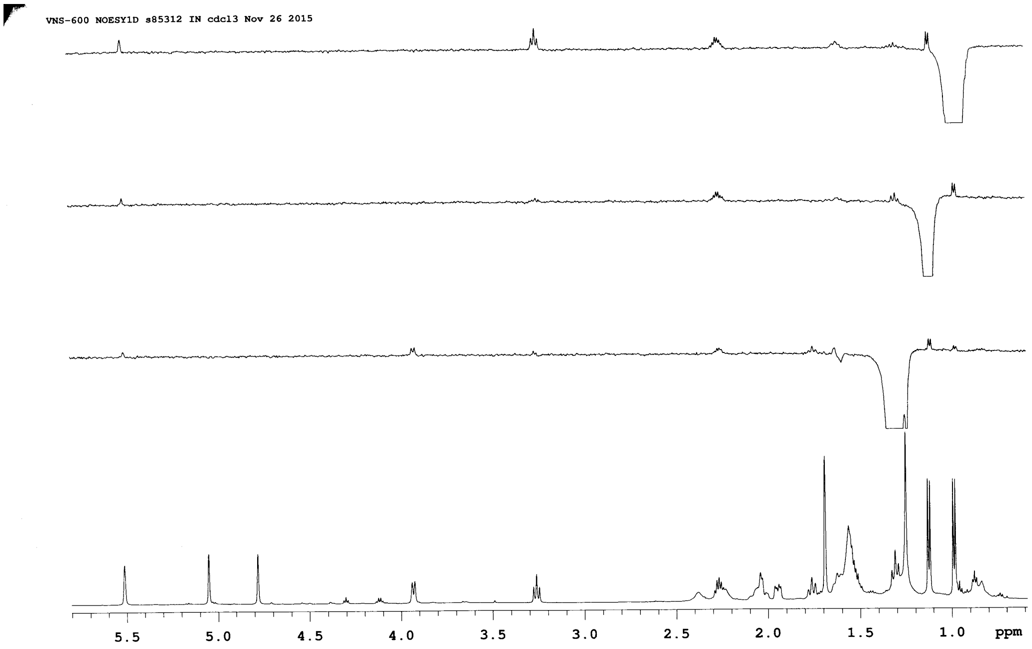

Figure S36. HRESIMS spectrum of compound **4**

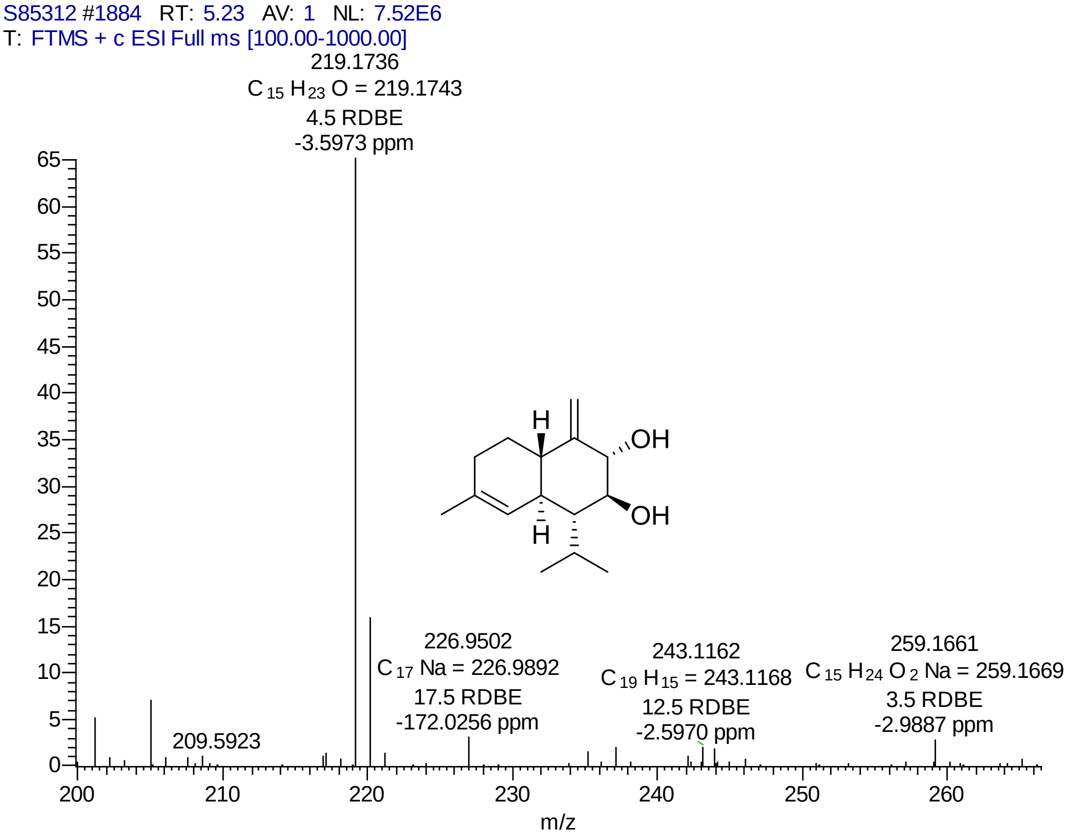

Figure S37. IR spectrum of compound **4**

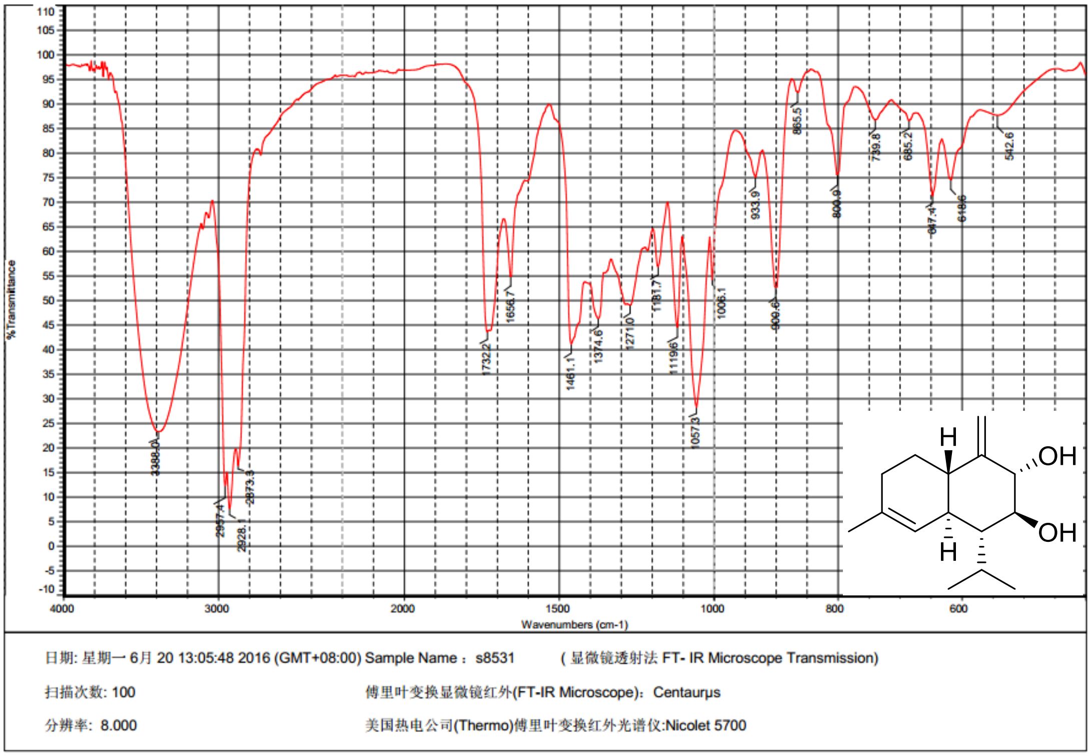

Figure S38. CD spectrum of compound **4**

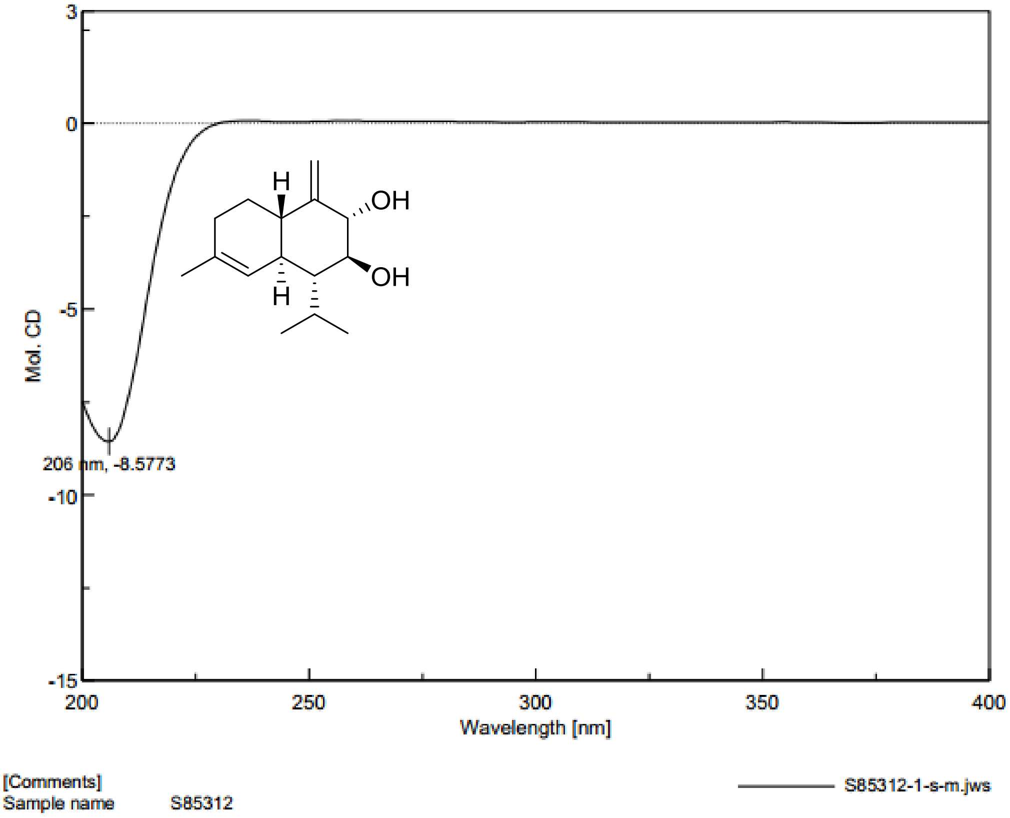

Figure S39. ^{1}H NMR spectrum of compound **5** in $CDCl_3$

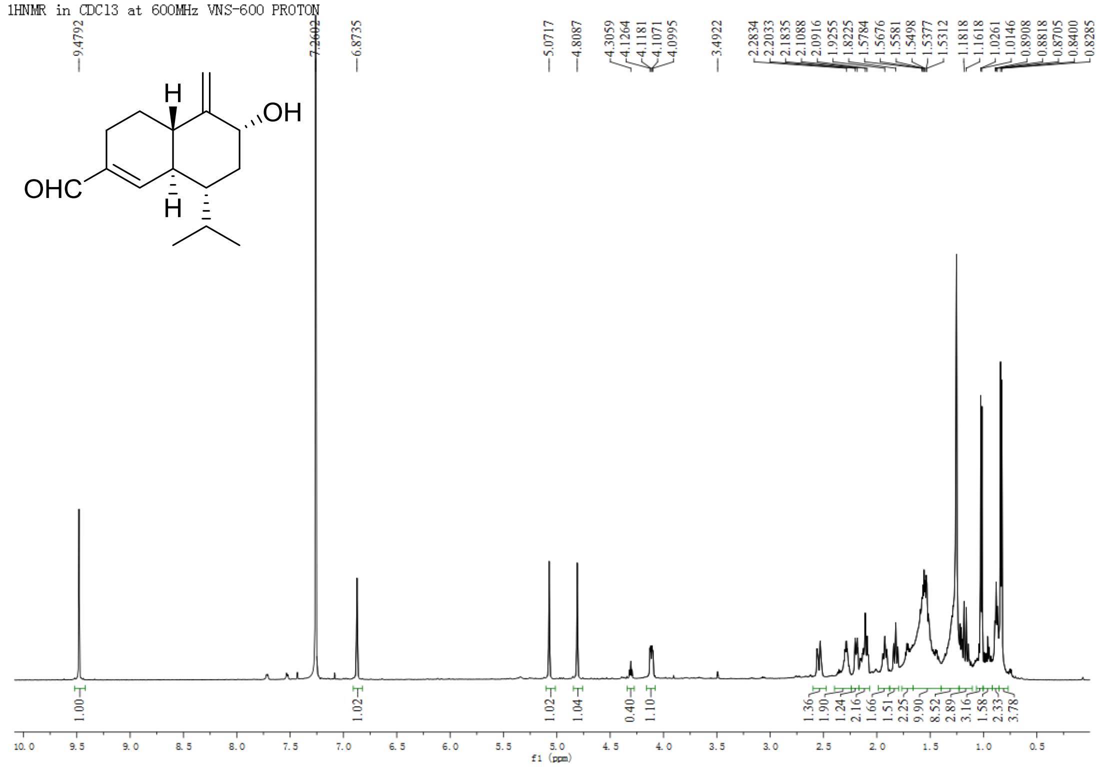

Figure S40. ^{13}C NMR spectrum of compound **5** in $CDCl_3$

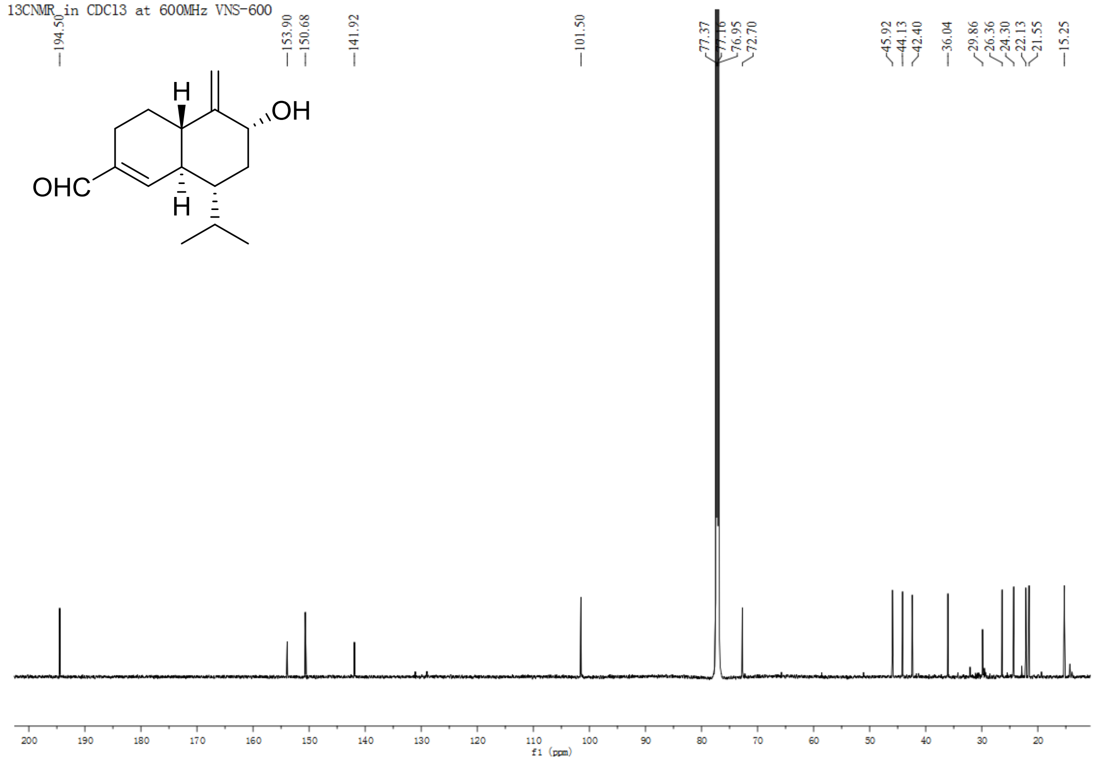

Figure S41. DEPT spectrum of compound **5** in $CDCl_3$

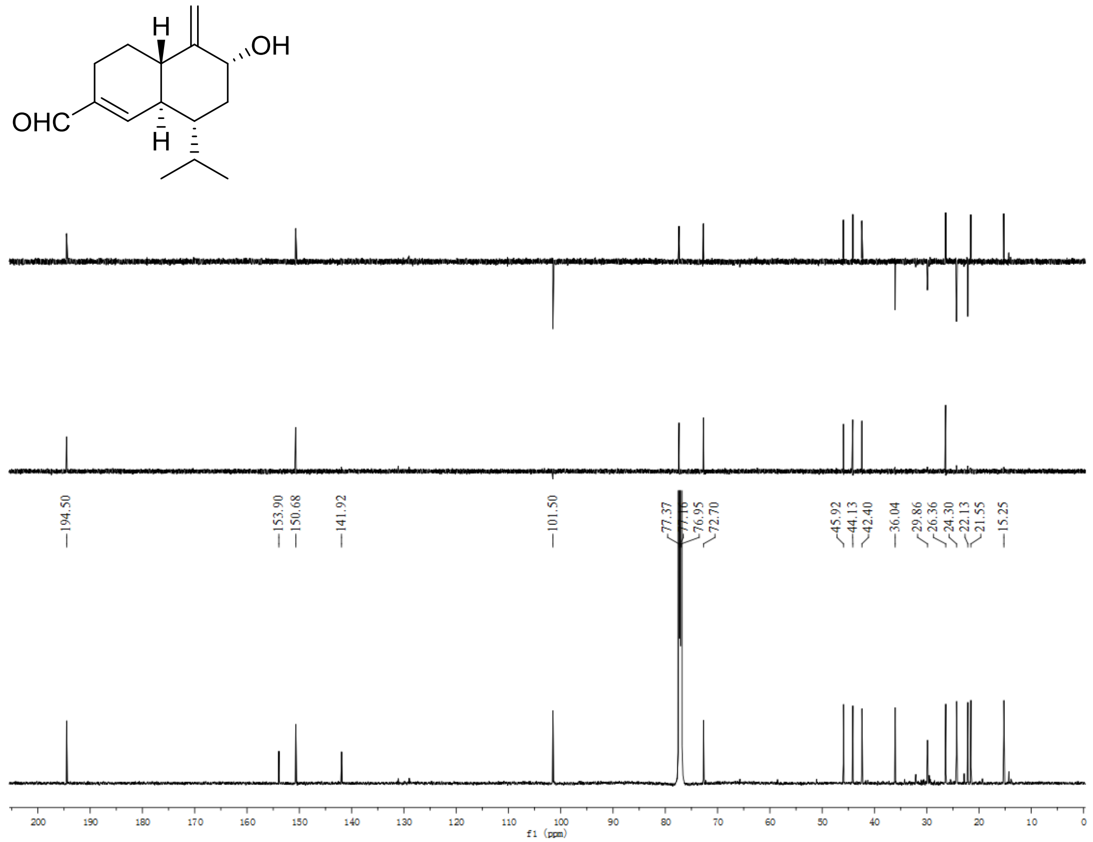

Figure S42. HSQC spectrum of compound **5** in $CDCl_3$

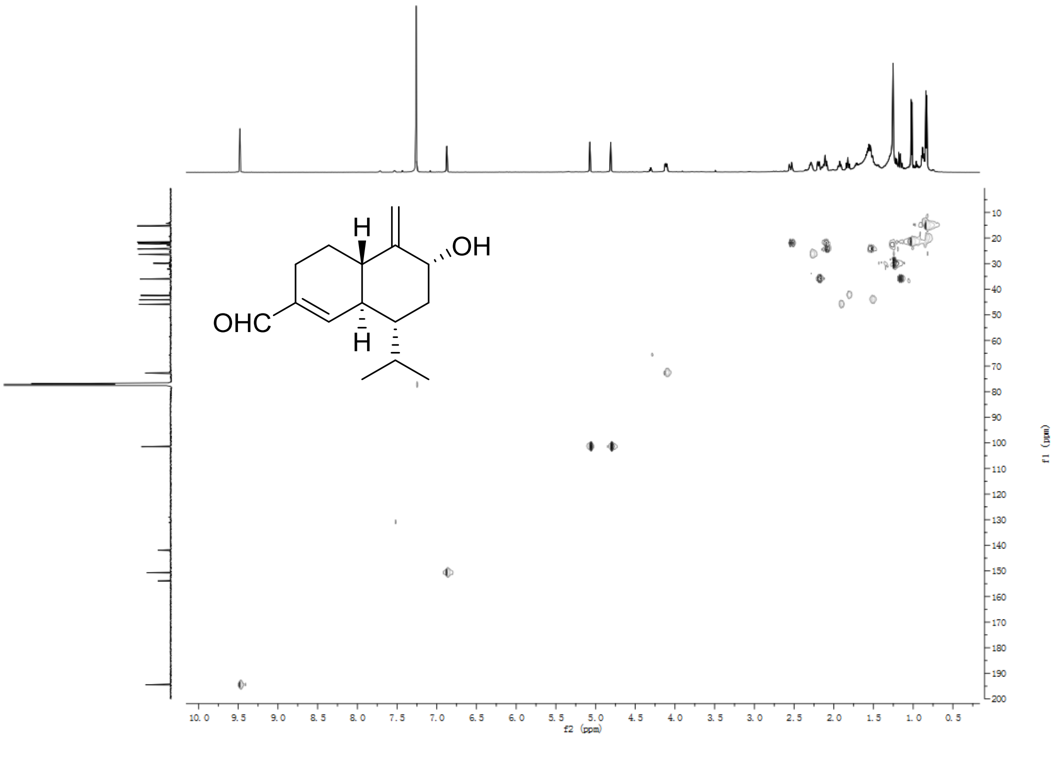

Figure S43. ^{1}H-^{1}H COSY spectrum of compound **5** in $CDCl_3$

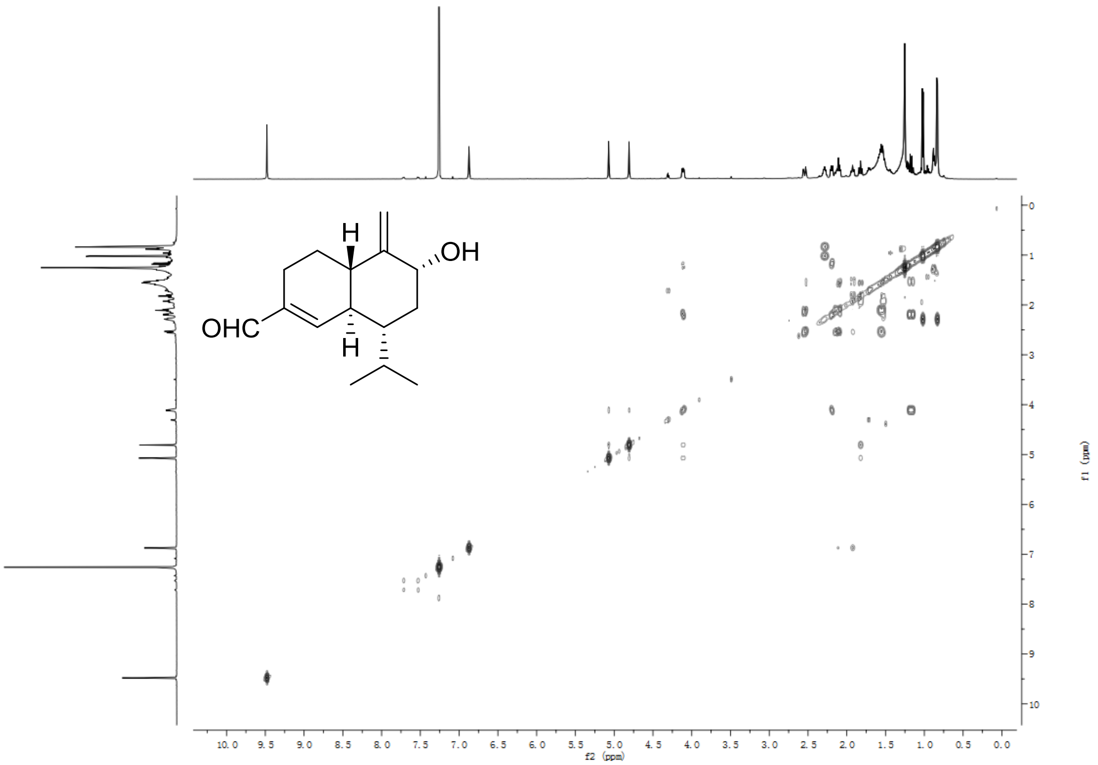

Figure S44. HMBC spectrum of compound **5** in $CDCl_3$

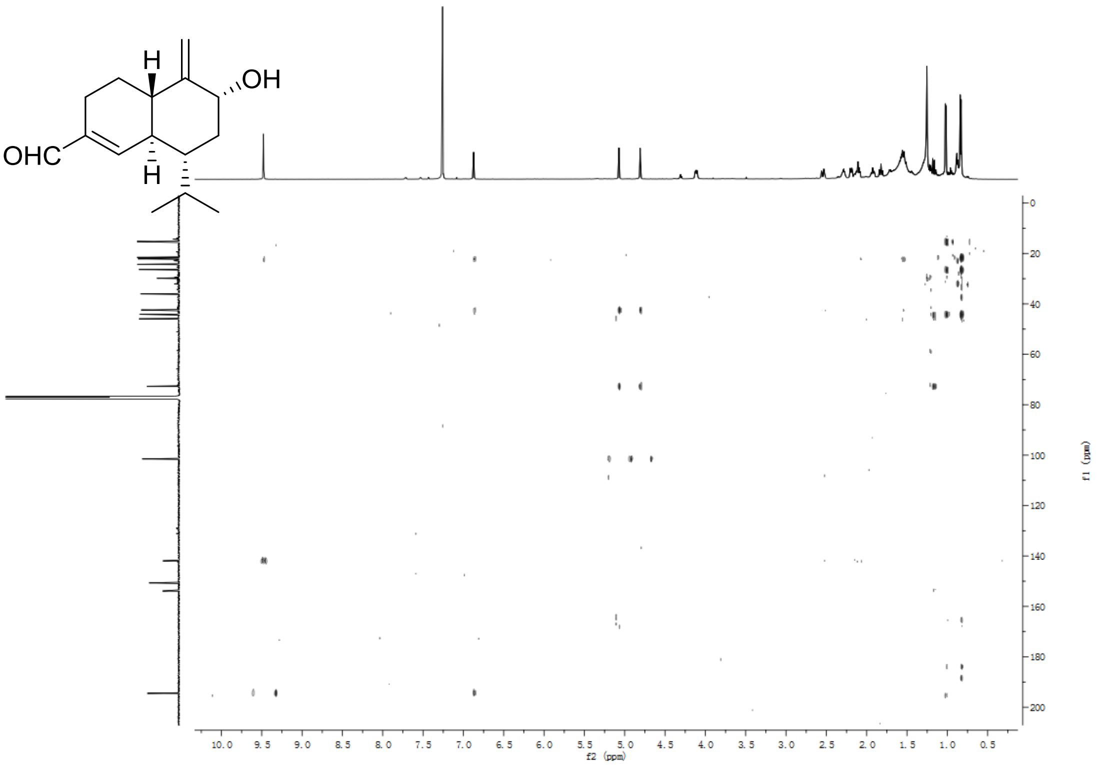

Figure S45. NOESY spectrum of compound **5** in $CDCl_3$

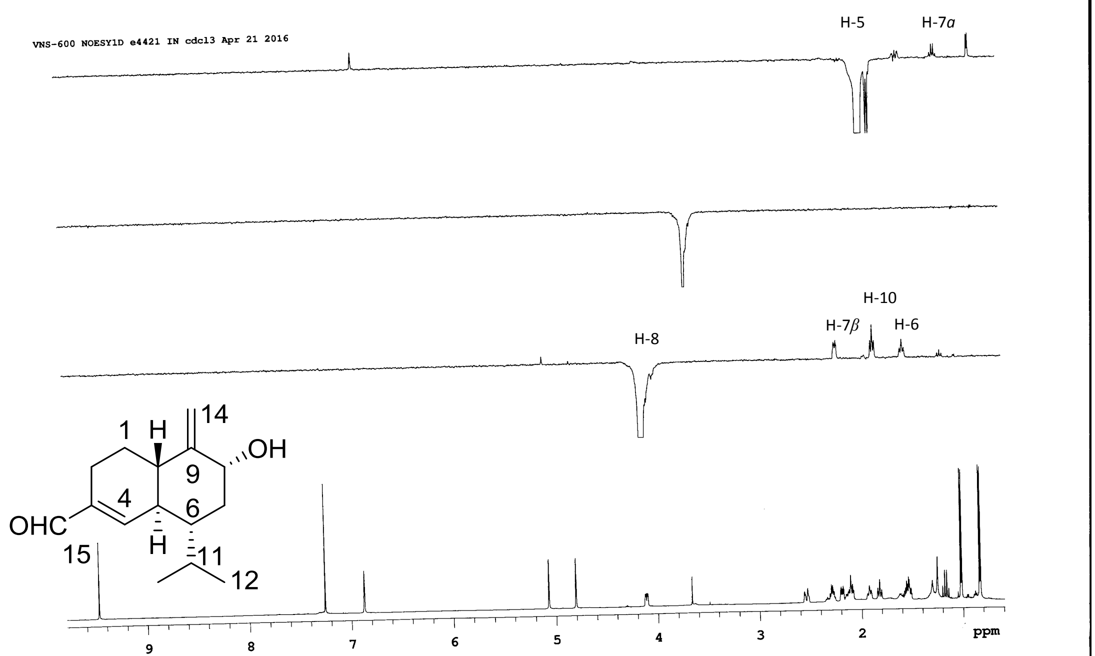

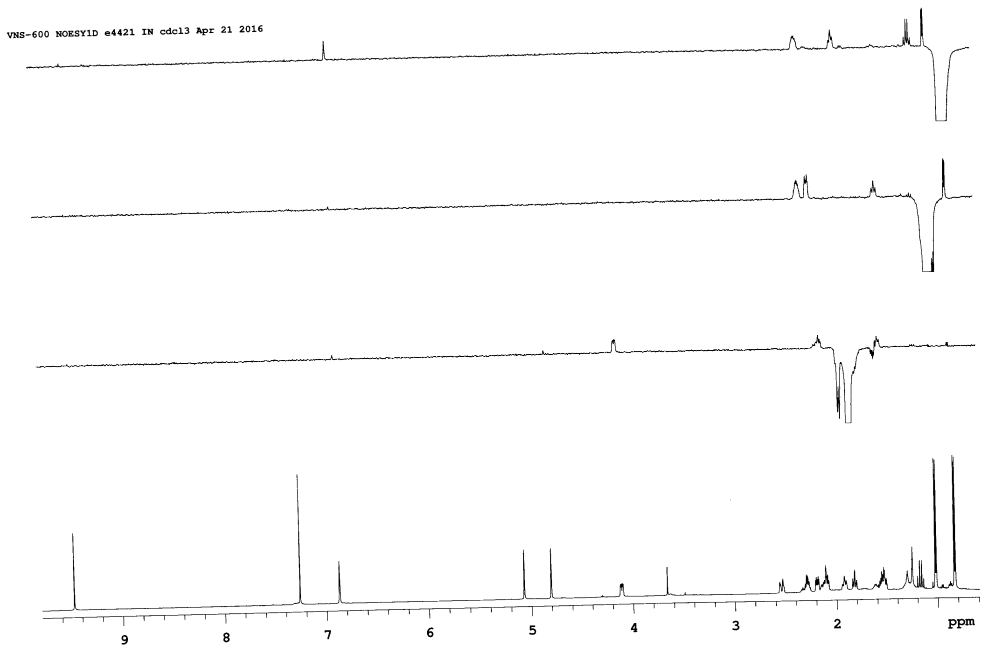

Figure S46. HRESIMS spectrum of compound **5**

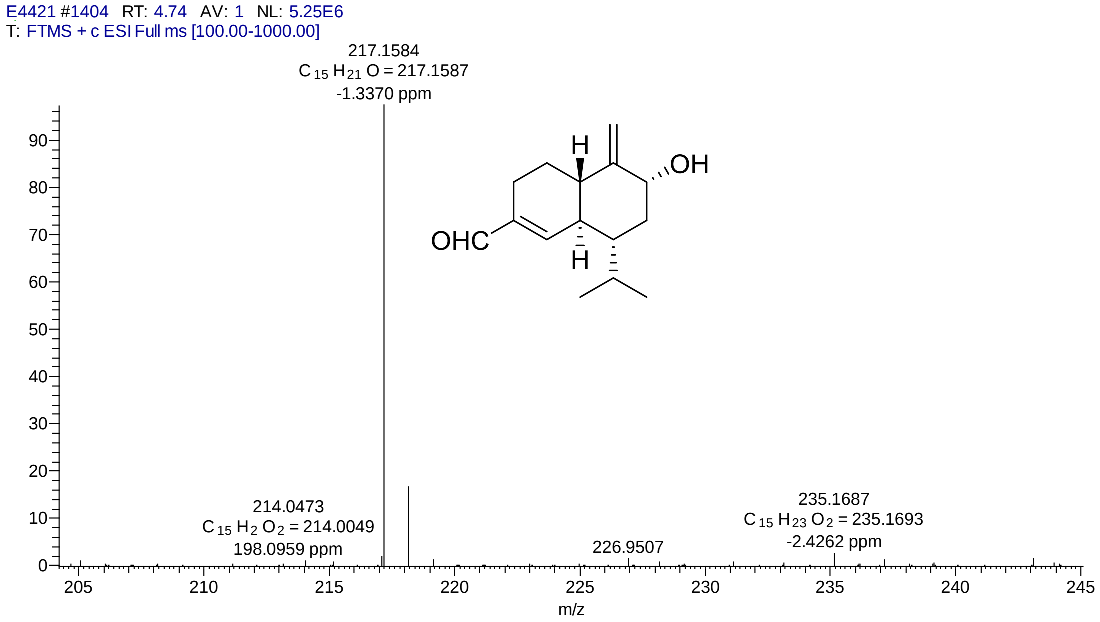

Figure S47. IR spectrum of compound **5**

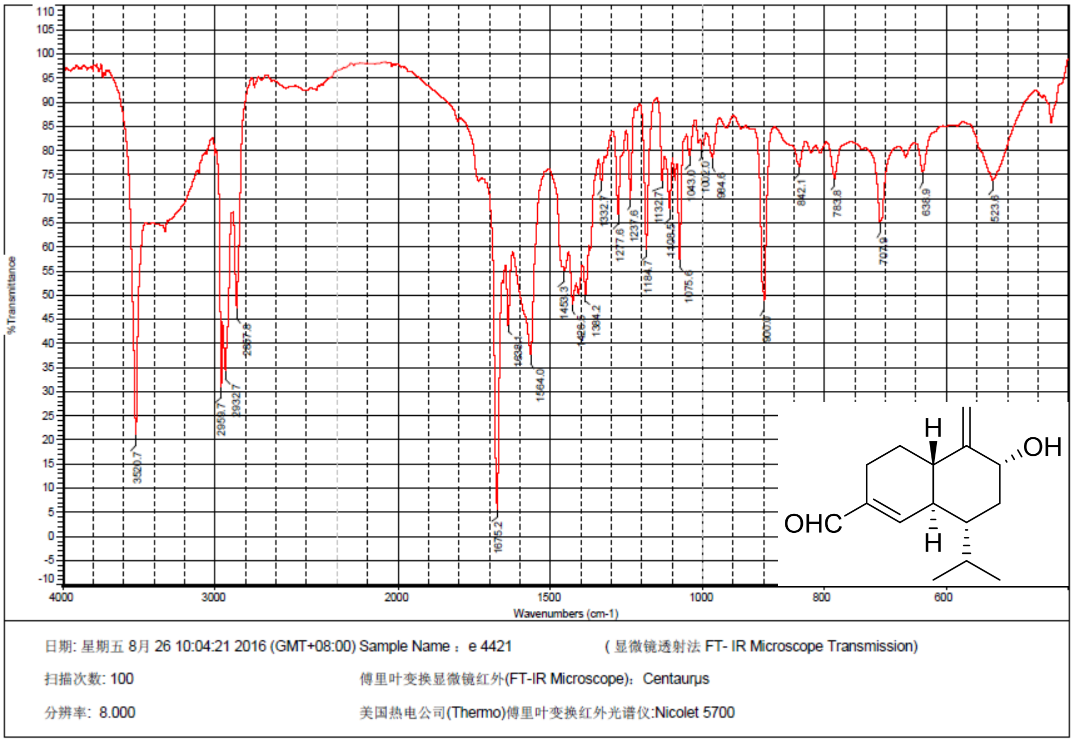

Figure S48. CD spectrum of compound **5**

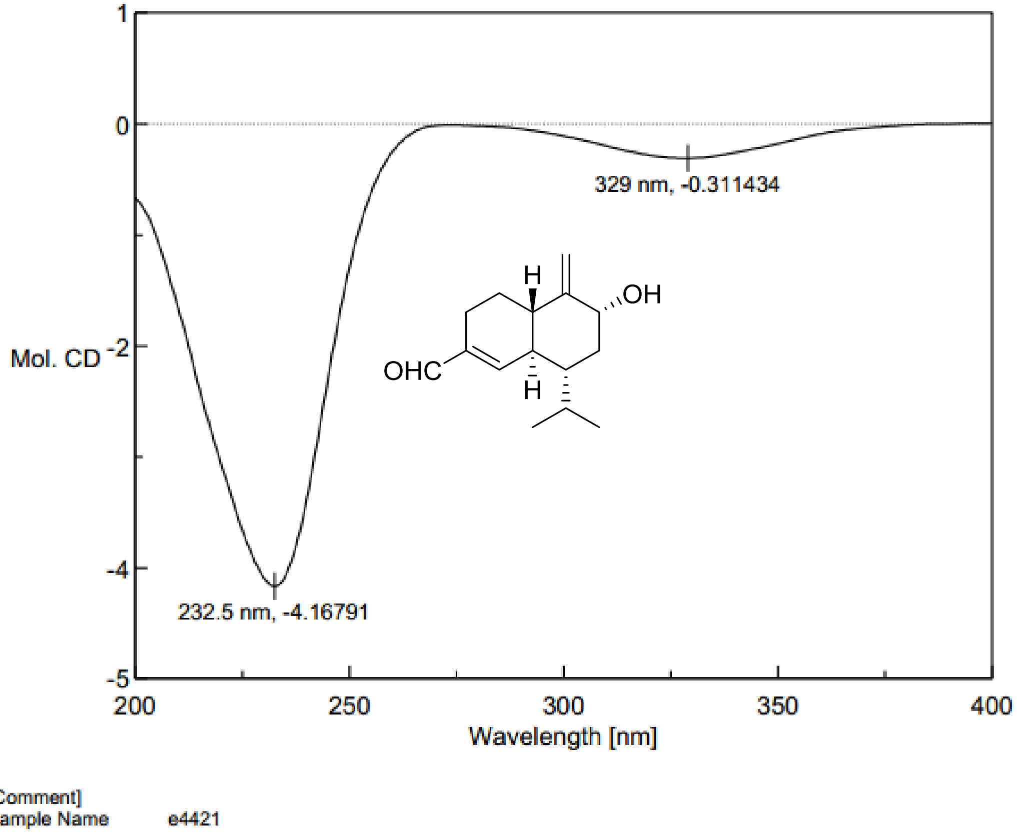

Figure S49. ^{1}H NMR spectrum of compound **6** in $CDCl_3$

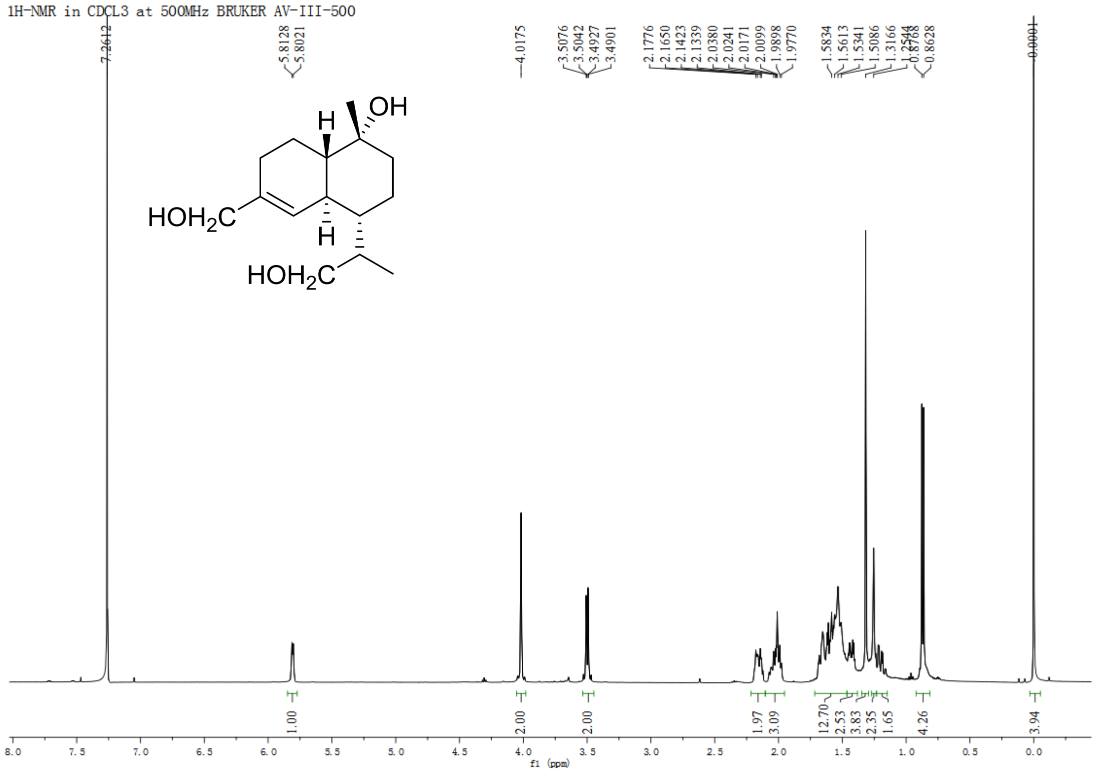

Figure S50. ^{13}C NMR spectrum of compound **6** in $CDCl_3$

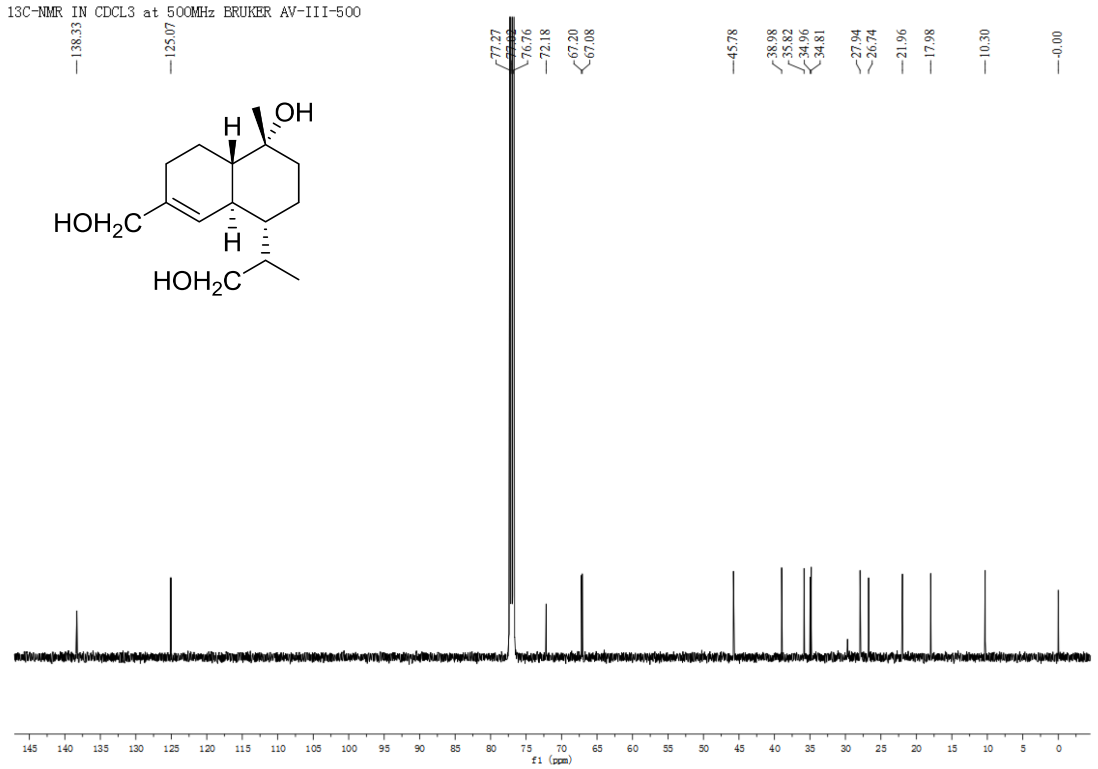

Figure S51. DEPT spectrum of compound **6** in $CDCl_3$

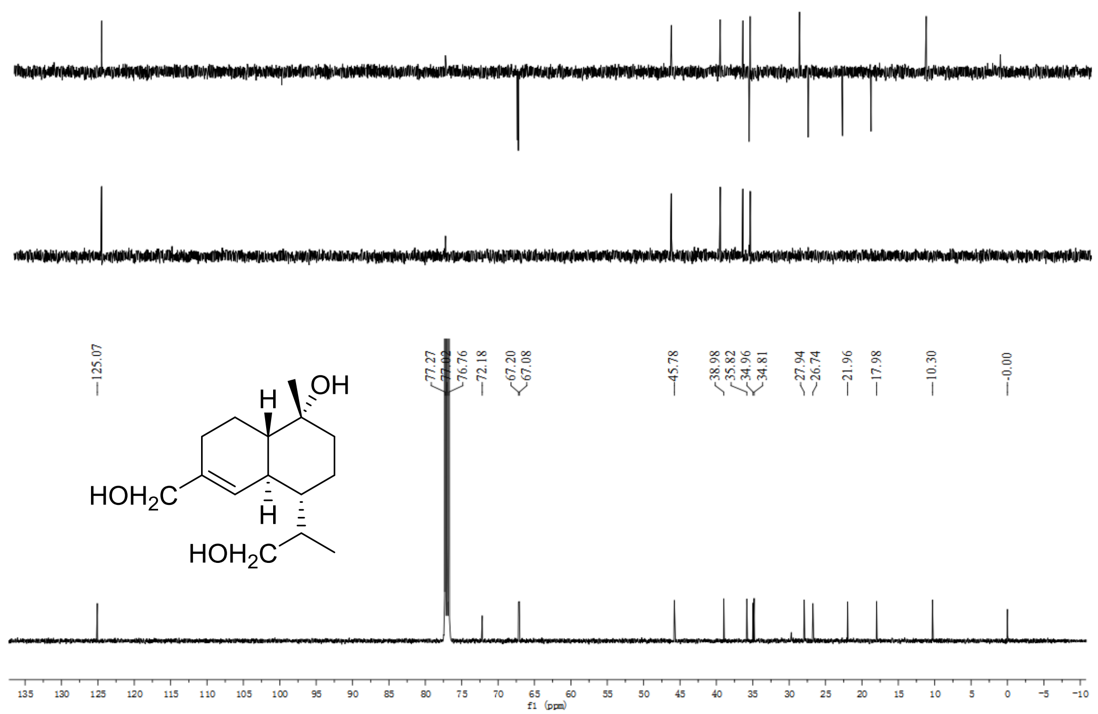

Figure S52. HSQC spectrum of compound **6** in $CDCl_3$

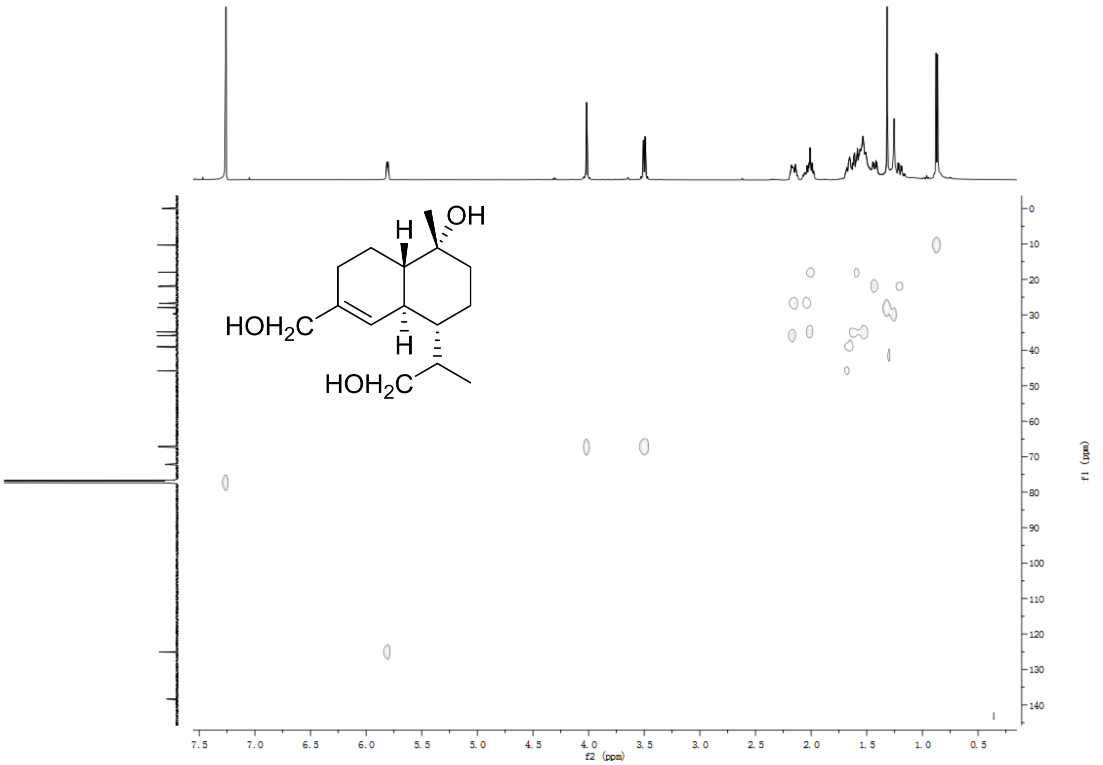

Figure S53. ^1H-^1H COSY spectrum of compound **6** in $CDCl_3$

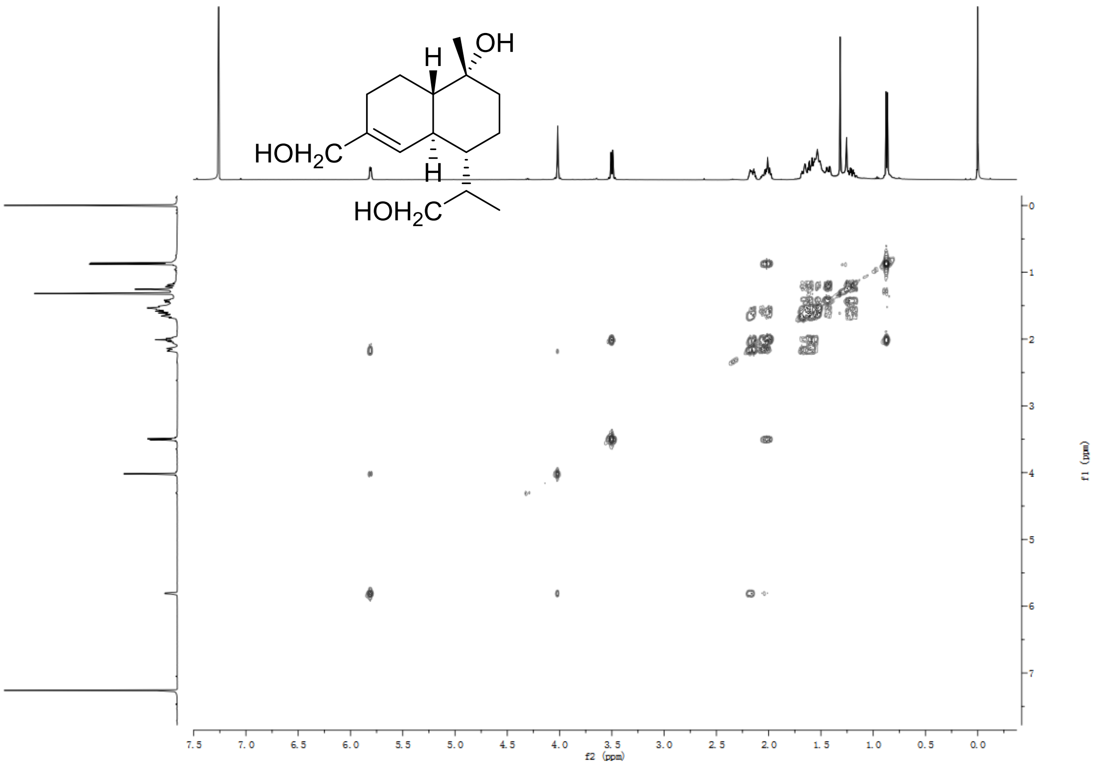

Figure S54. HMBC spectrum of compound **6** in $CDCl_3$

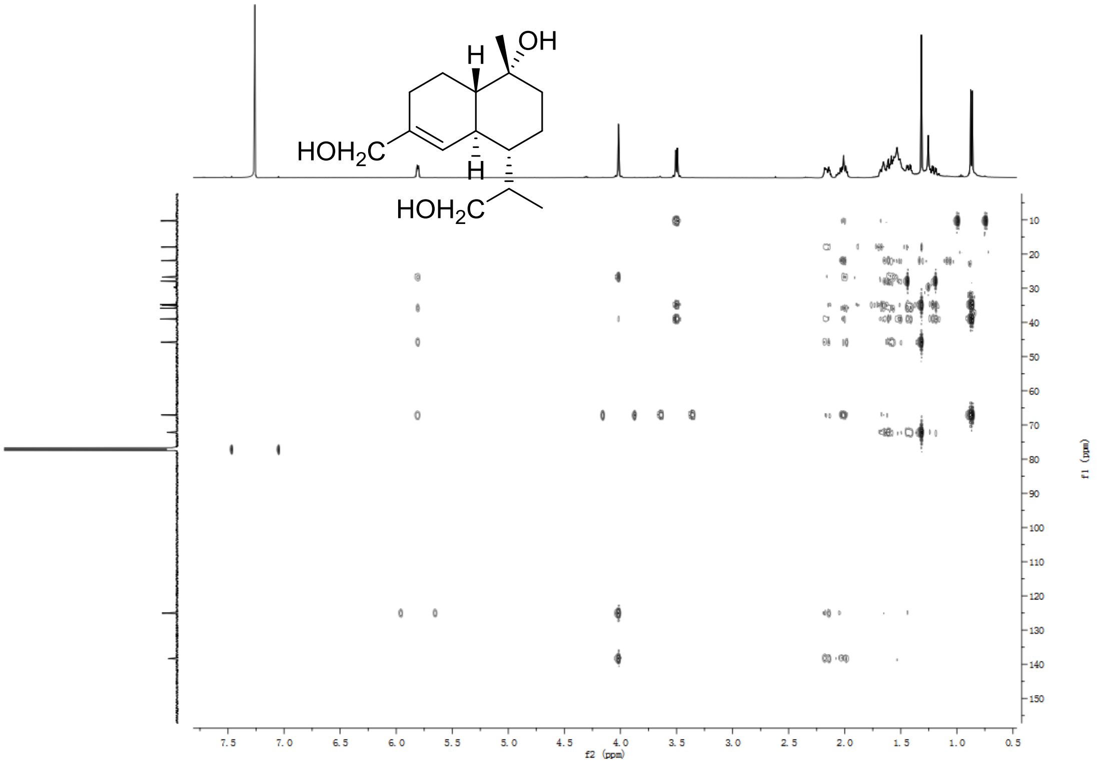

Figure S55. NOESY spectrum of compound **6** in $CDCl_3$

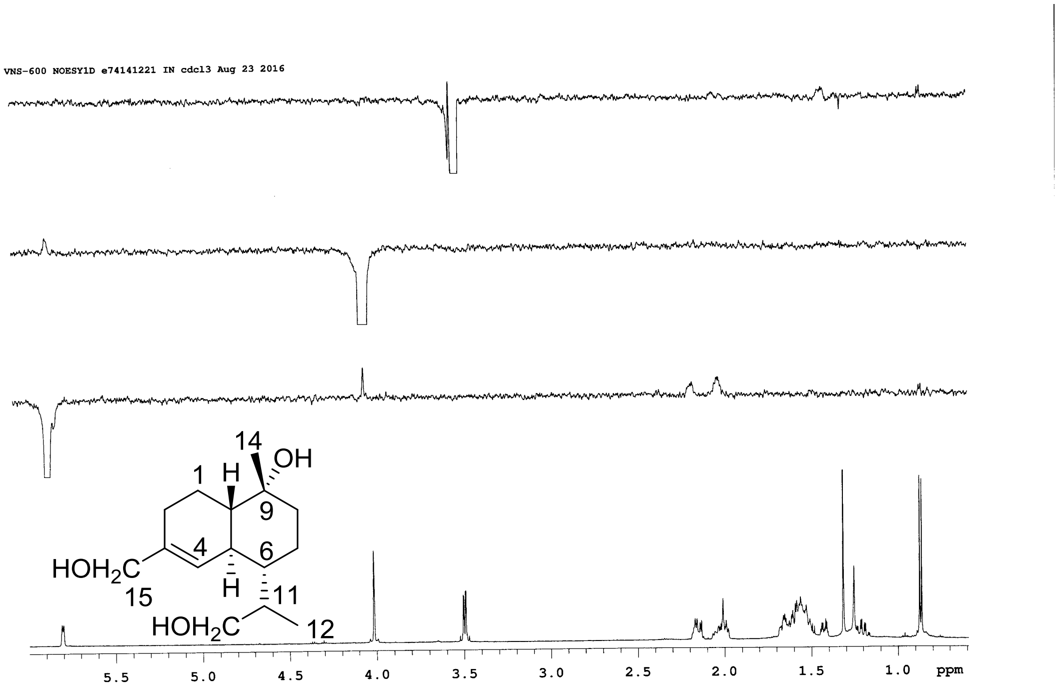

VNS-600 NOESY1D e74141221 IN cdcl3 Aug 23 2016

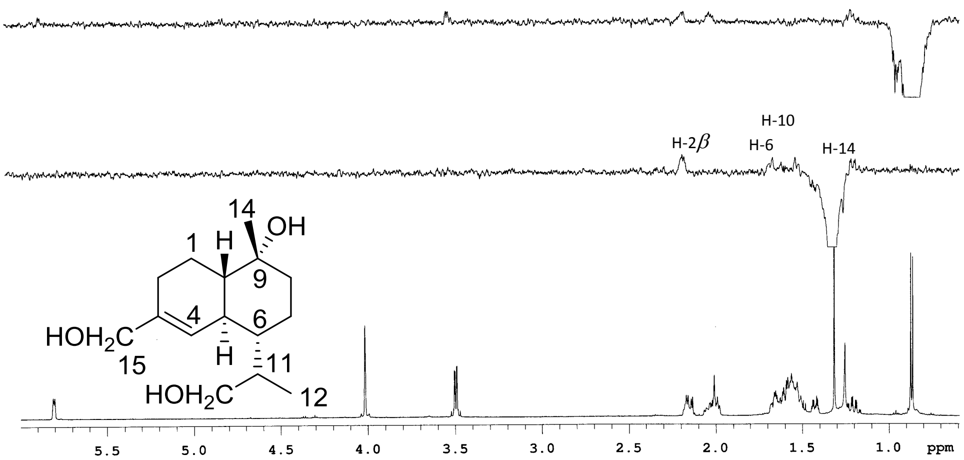

VNS-600 NOESY1D e74141221 IN cdcl3 Aug 25 2016

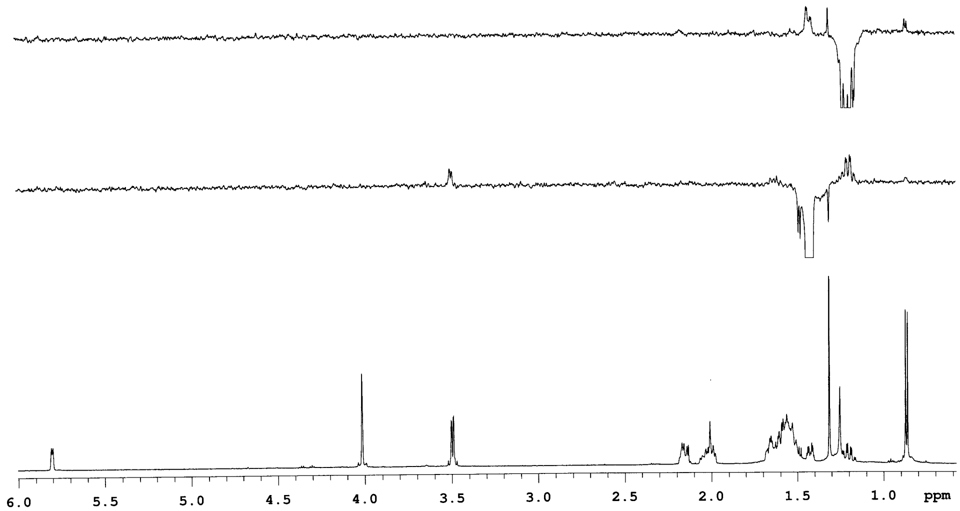

Figure S56. HRESIMS spectrum of compound **6**

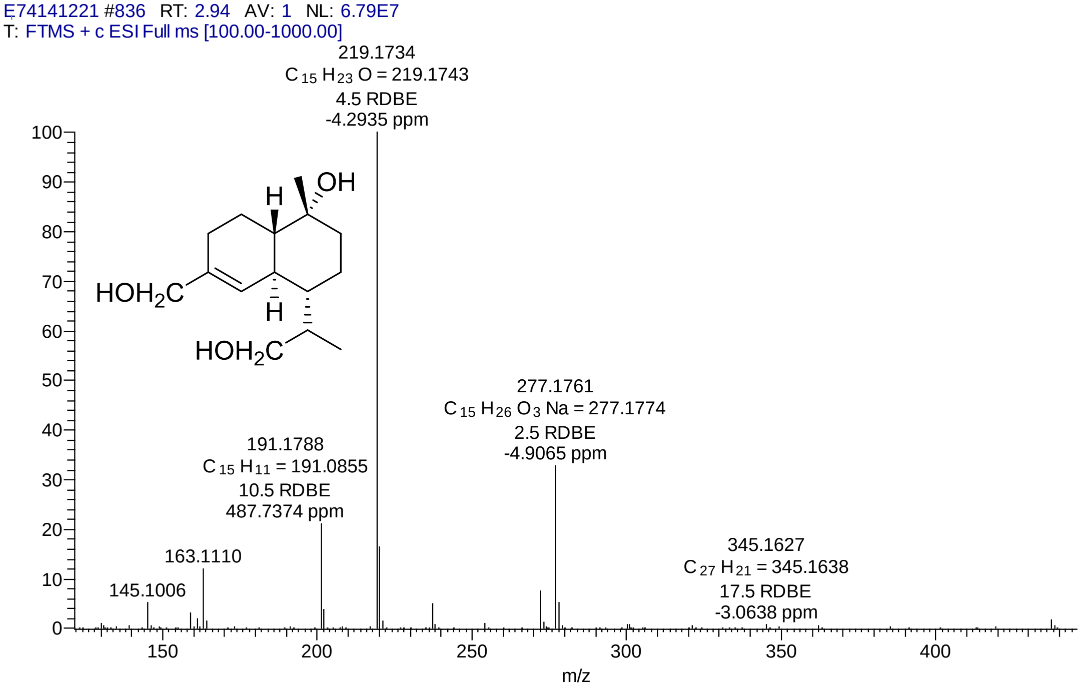

Figure S57. IR spectrum of compound **6**

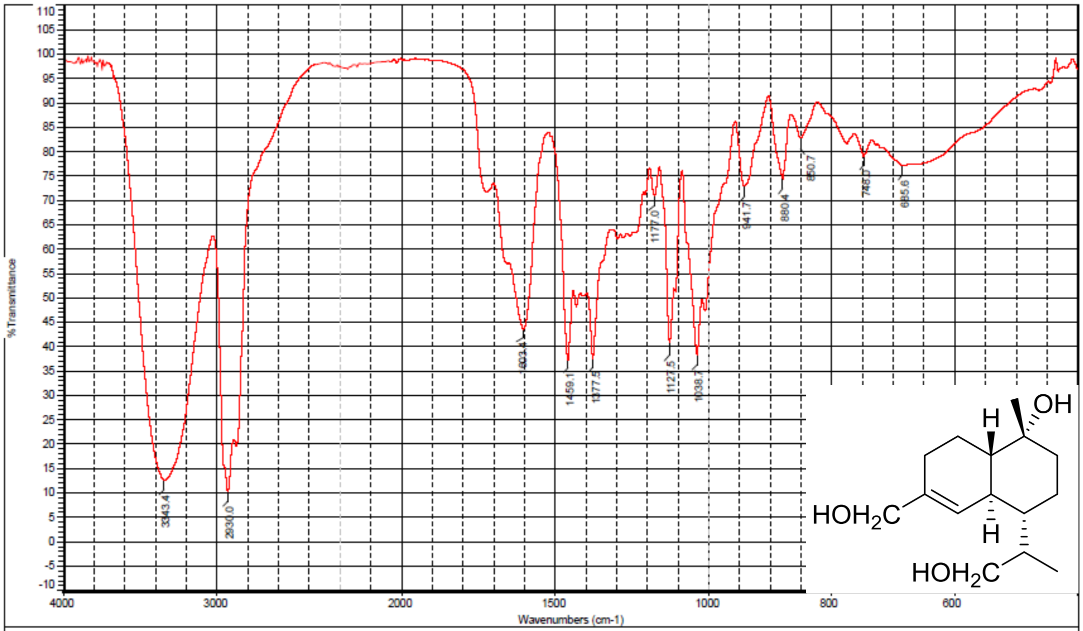

日期: 星期五 8月 26 10:40:14 2016 (GMT+08:00) Sample Name : e 74141221 (显微镜透射法 FT- IR Microscope Transmission)

扫描次数: 100 傅里叶变换显微镜红外(FT-IR Microscope): Centaurμs

分辨率: 8.000 美国热电公司(Thermo)傅里叶变换红外光谱仪:Nicolet 5700

Figure S58. ^{1}H NMR spectrum of compound **7** in $CDCl_3$

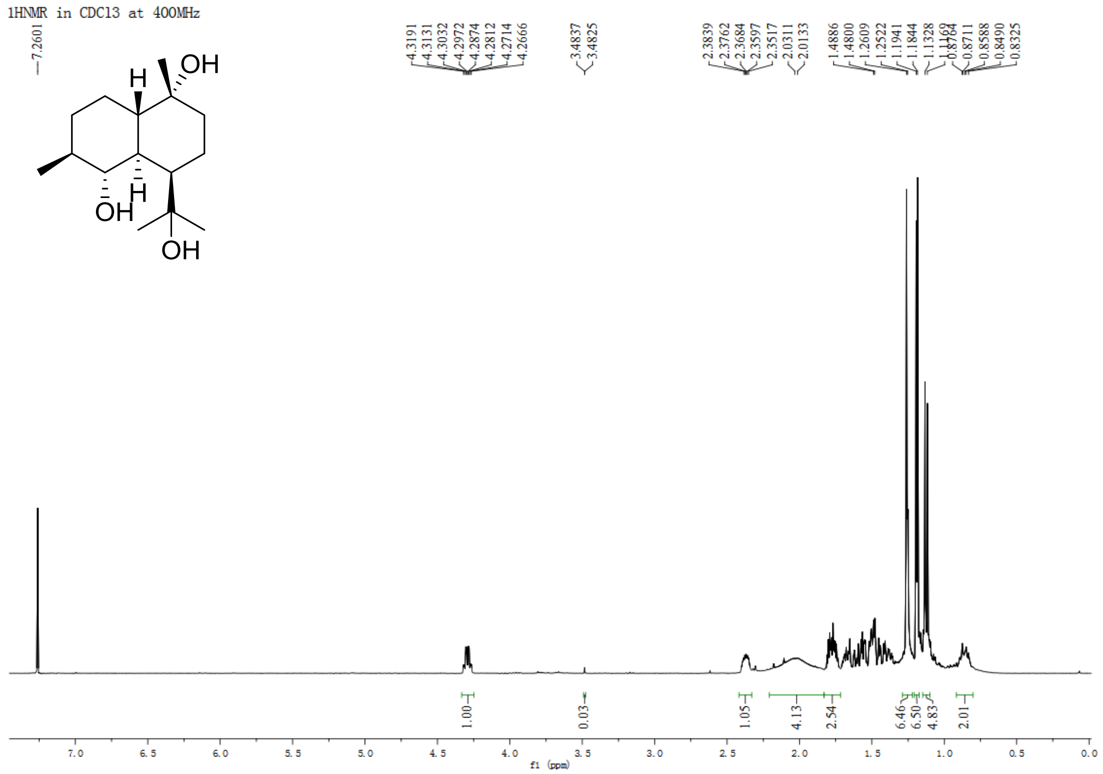

Figure S59. ^{13}C NMR spectrum of compound **7** in $CDCl_3$

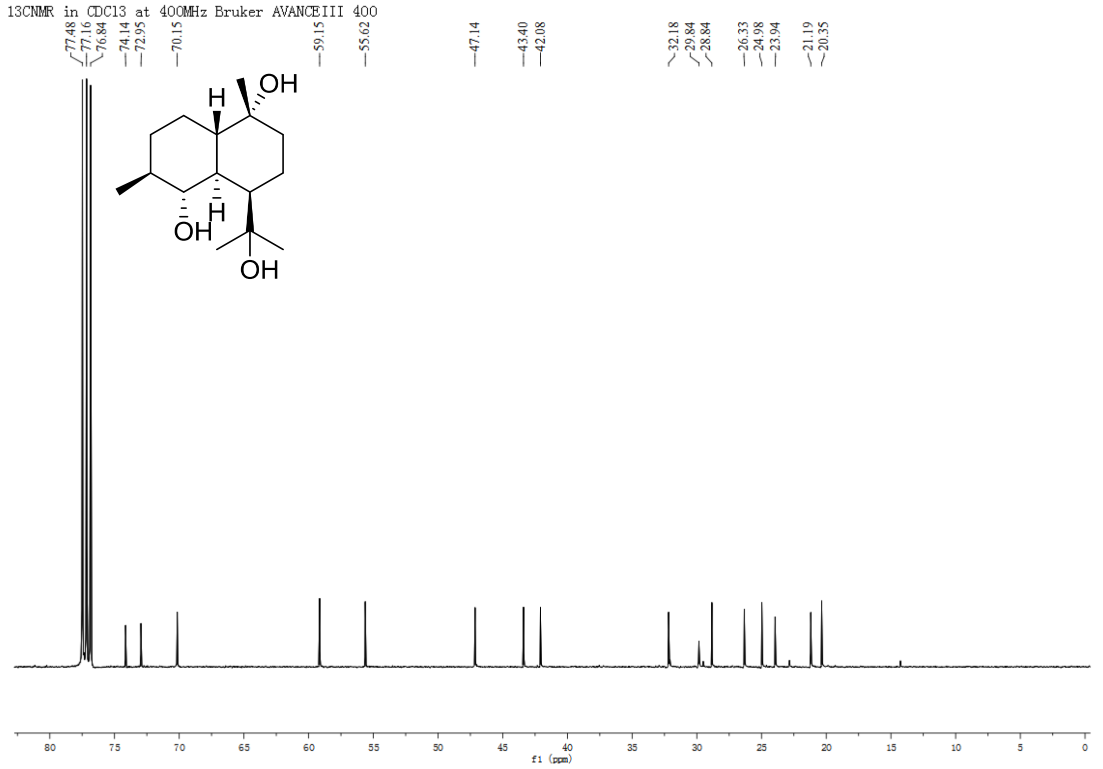

Figure S60. DEPT spectrum of compound **7** in $CDCl_3$

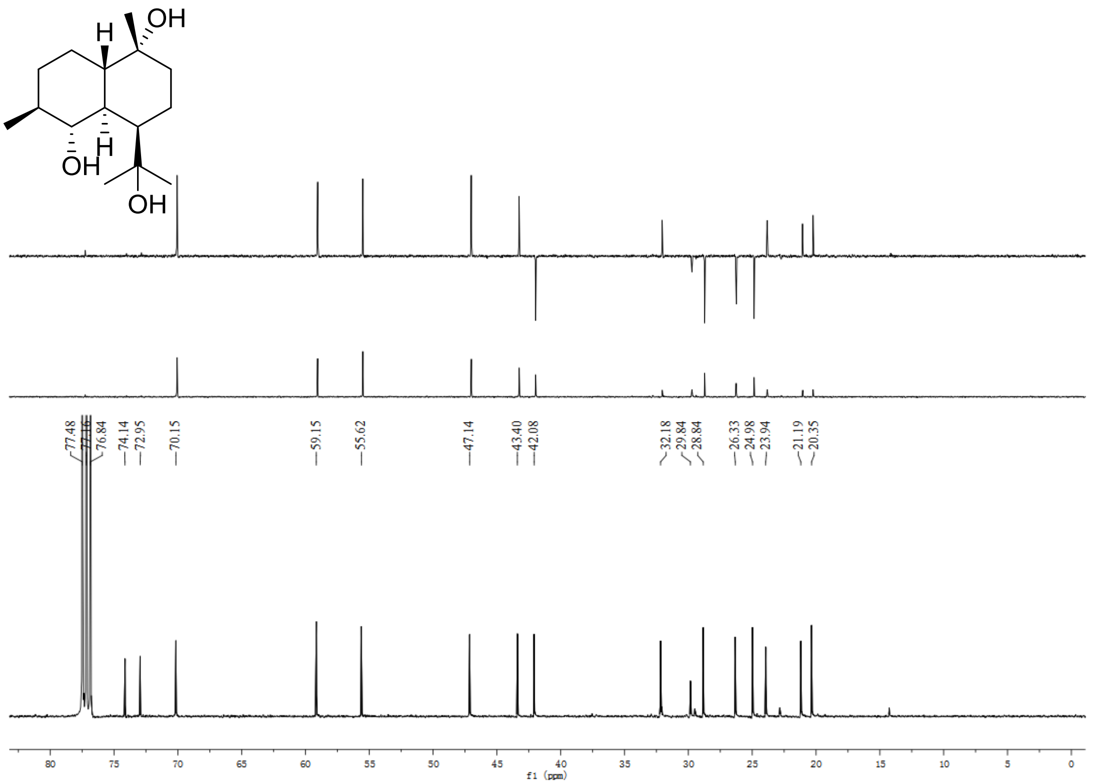

Figure S61. HSQC spectrum of compound **7** in $CDCl_3$

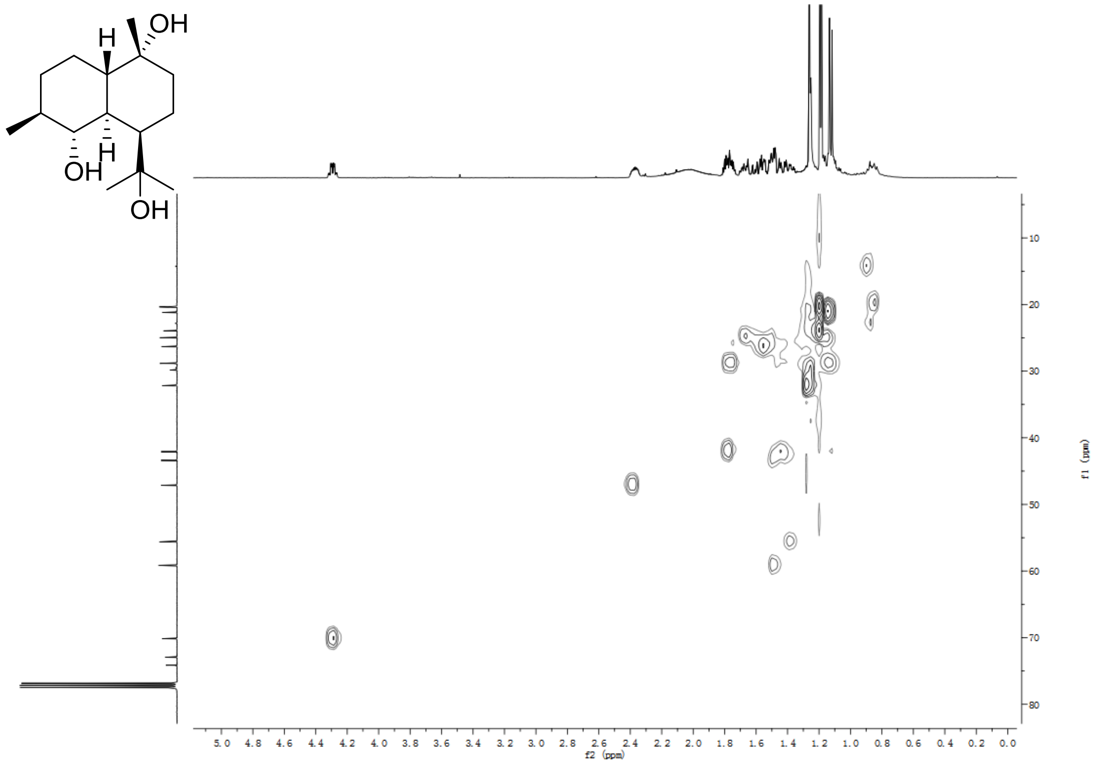

Figure S62. ^{1}H-^{1}H COSY spectrum of compound **7** in $CDCl_3$

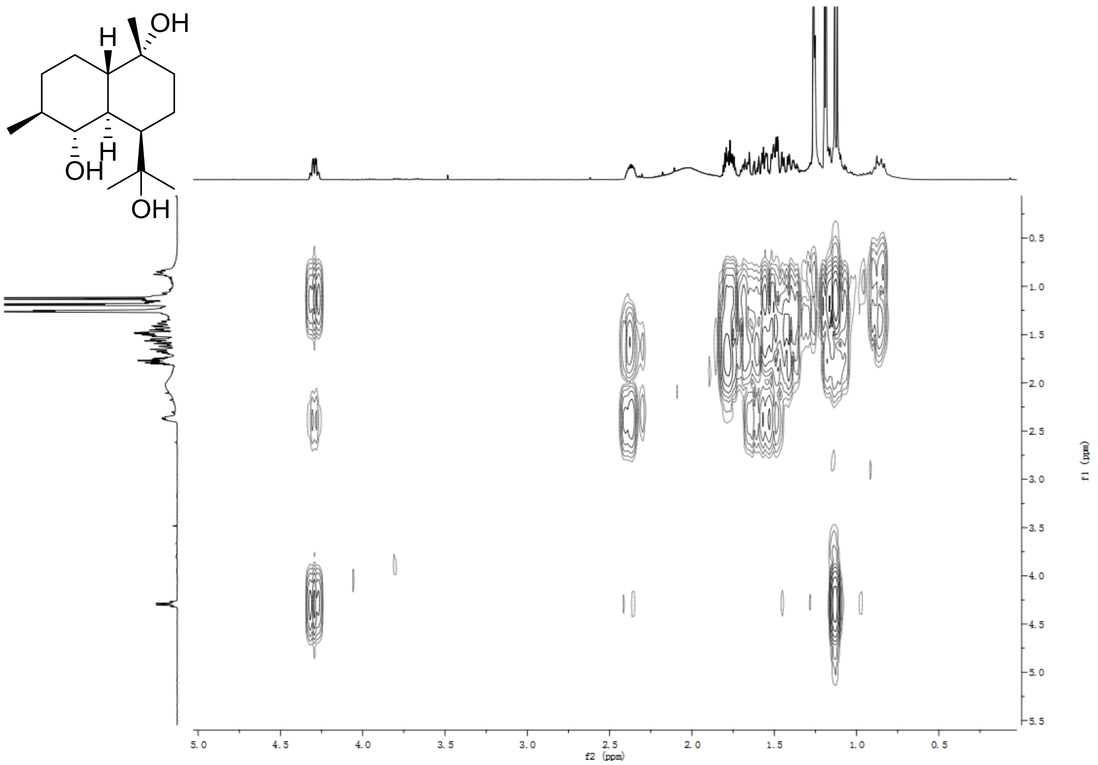

Figure S63. HMBC spectrum of compound **7** in $CDCl_3$

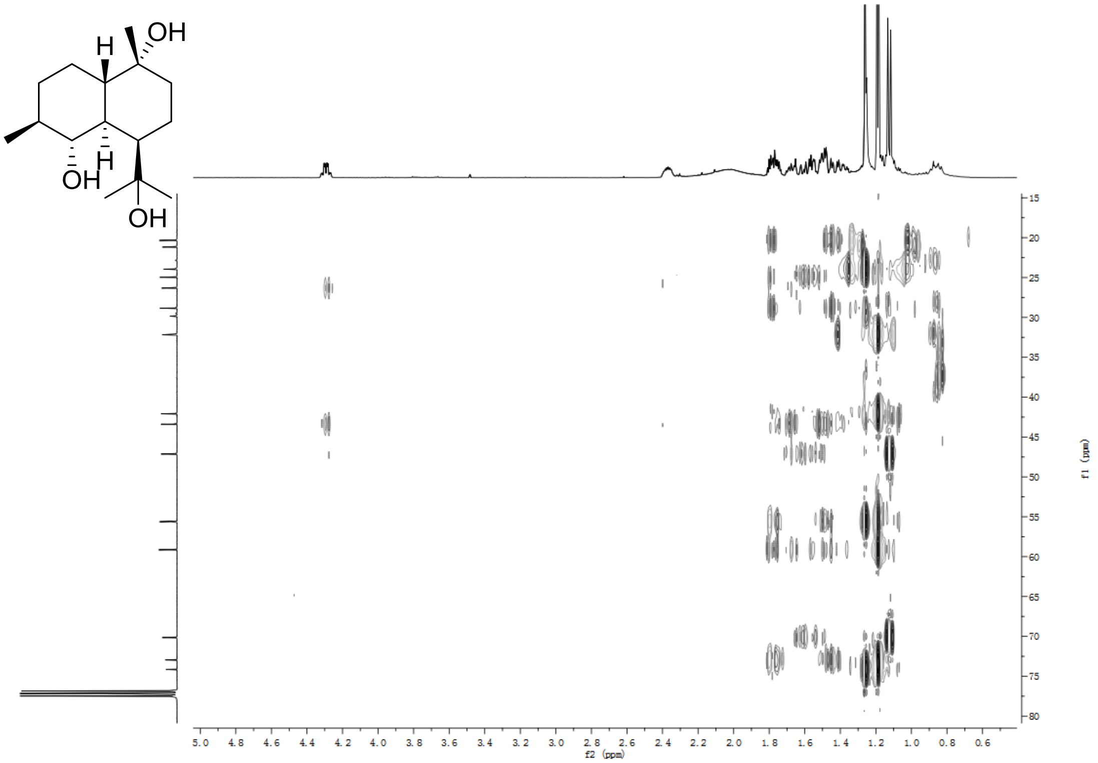

Figure S64. NOESY spectrum of compound **7** in $CDCl_3$

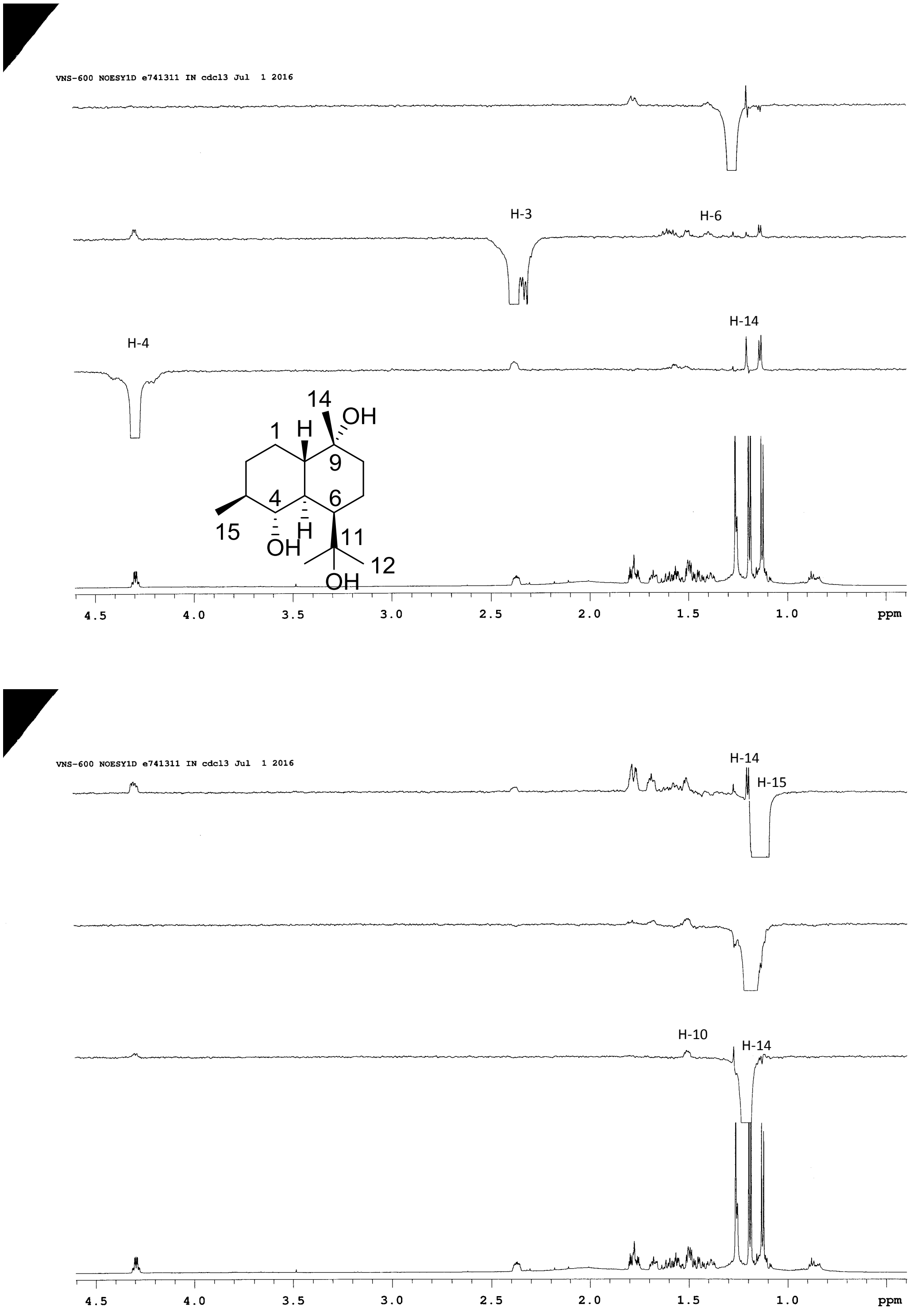

Figure S65. HRESIMS spectrum of compound **7**

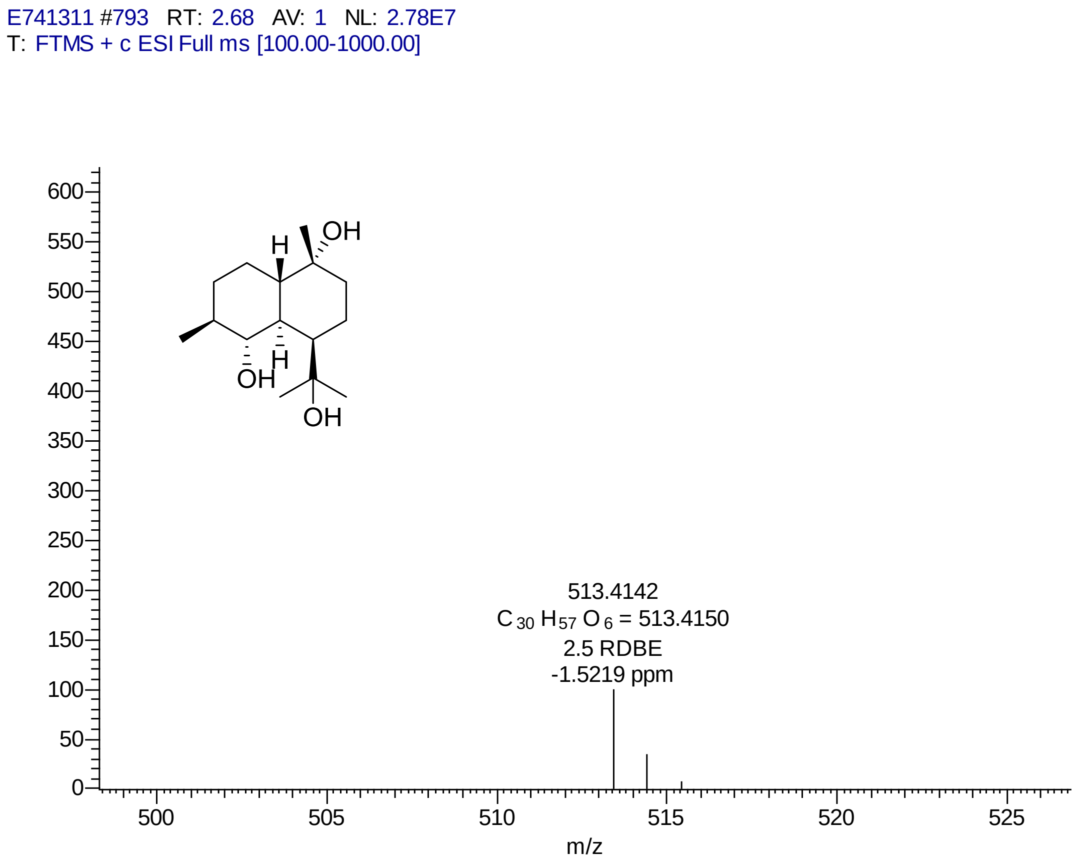

Figure S66. IR spectrum of compound **7**

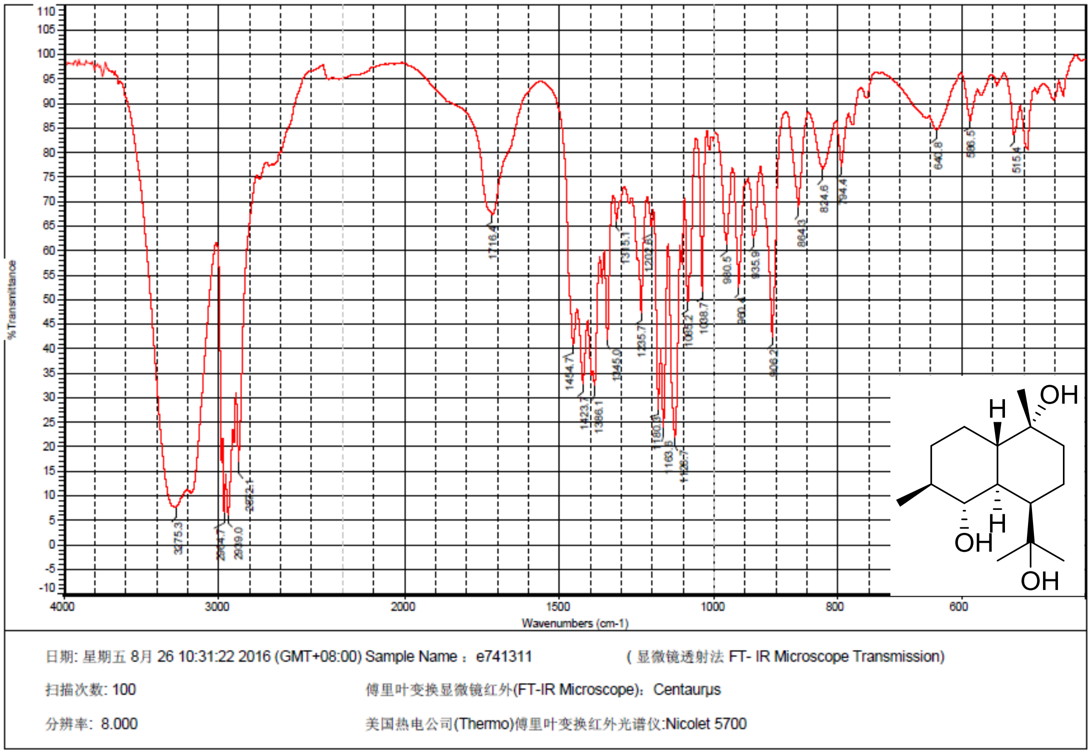

Figure S67. ^{1}H NMR spectrum of compound 4-Benzoyl-ganodermanol G in $CDCl_3$

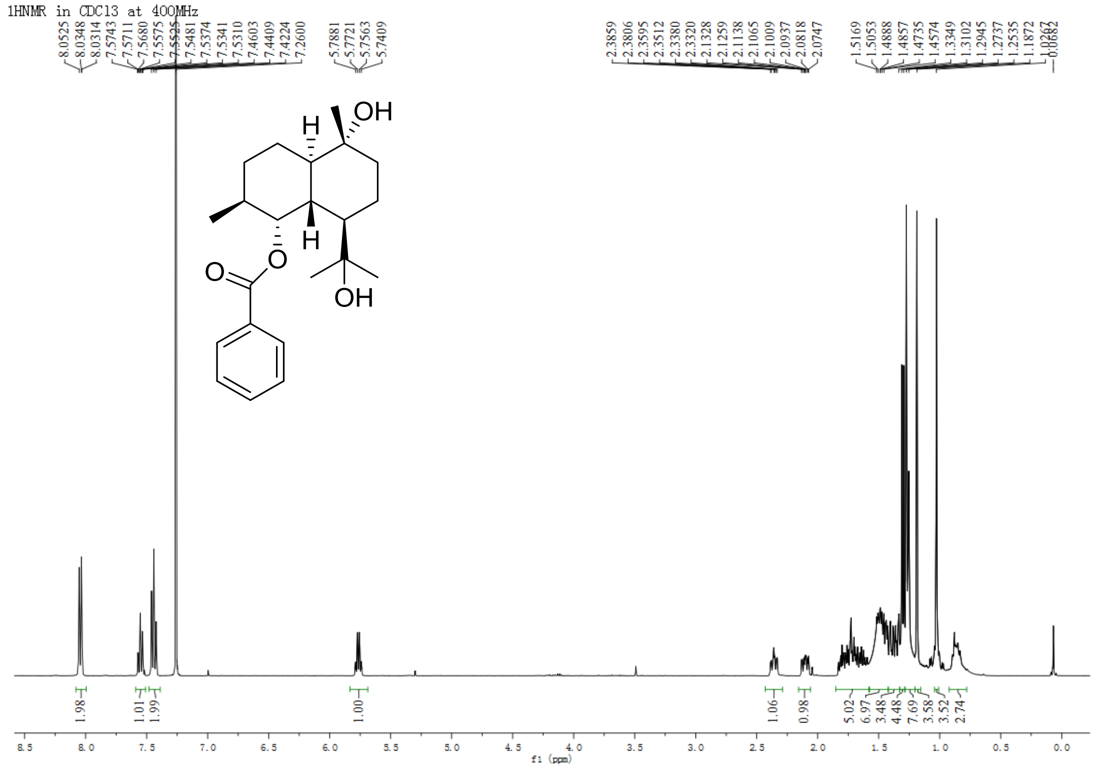

Figure S68. ^{13}C NMR spectrum of compound 4-Benzoyl-ganodermanol H in $CDCl_3$

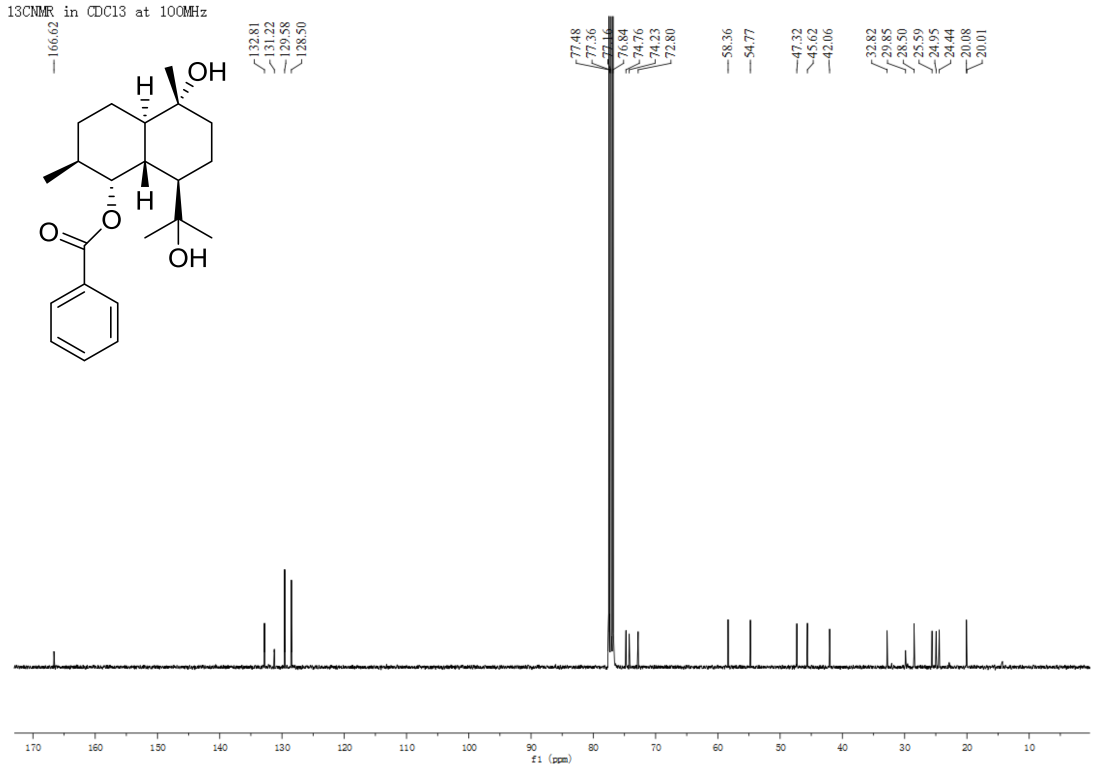

Figure S69. HMBC spectrum of compound 4-Benzoyl-ganodermanol G in $CDCl_3$

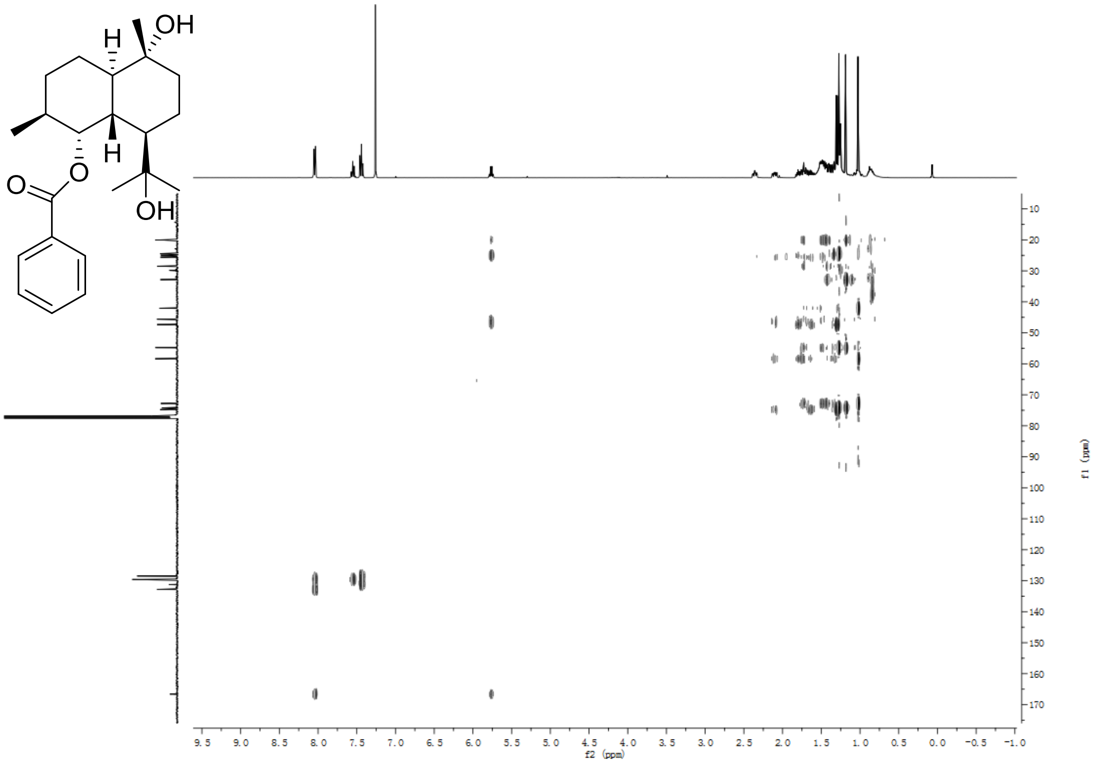

Figure S70. ^{1}H NMR spectrum of compound **8** in $CDCl_3$

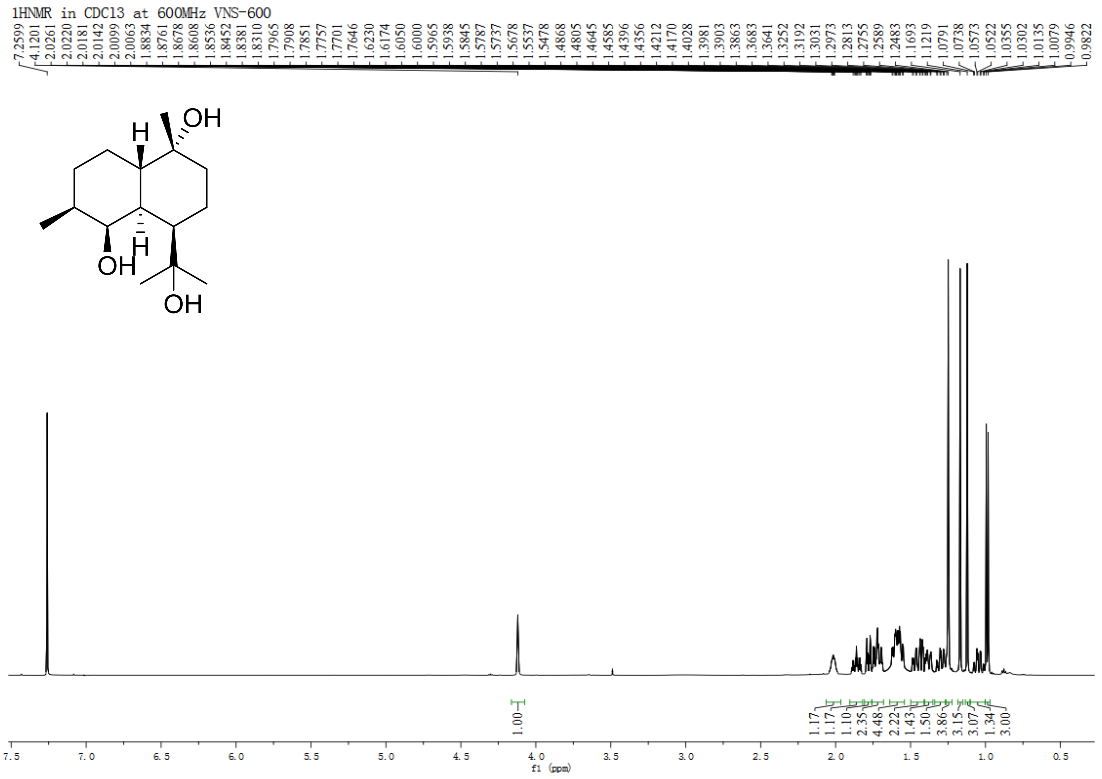

Figure S71. ^{13}C NMR spectrum of compound **8** in $CDCl_3$

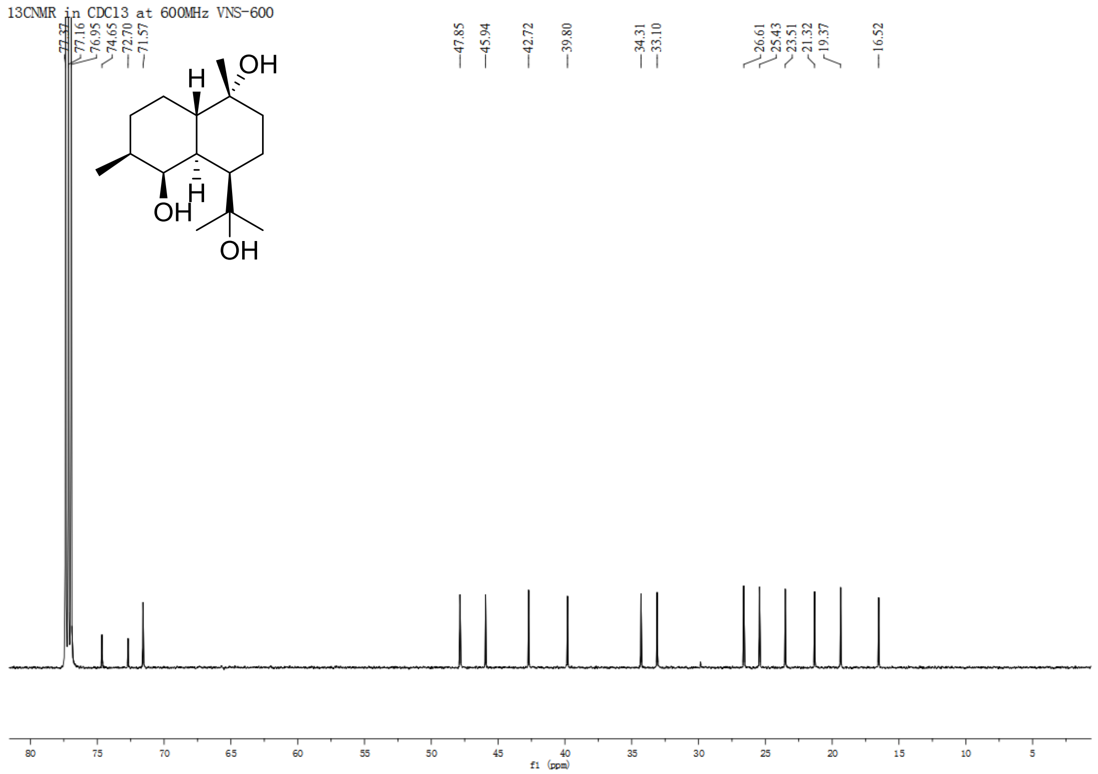

Figure S72. DEPT spectrum of compound **8** in $CDCl_3$

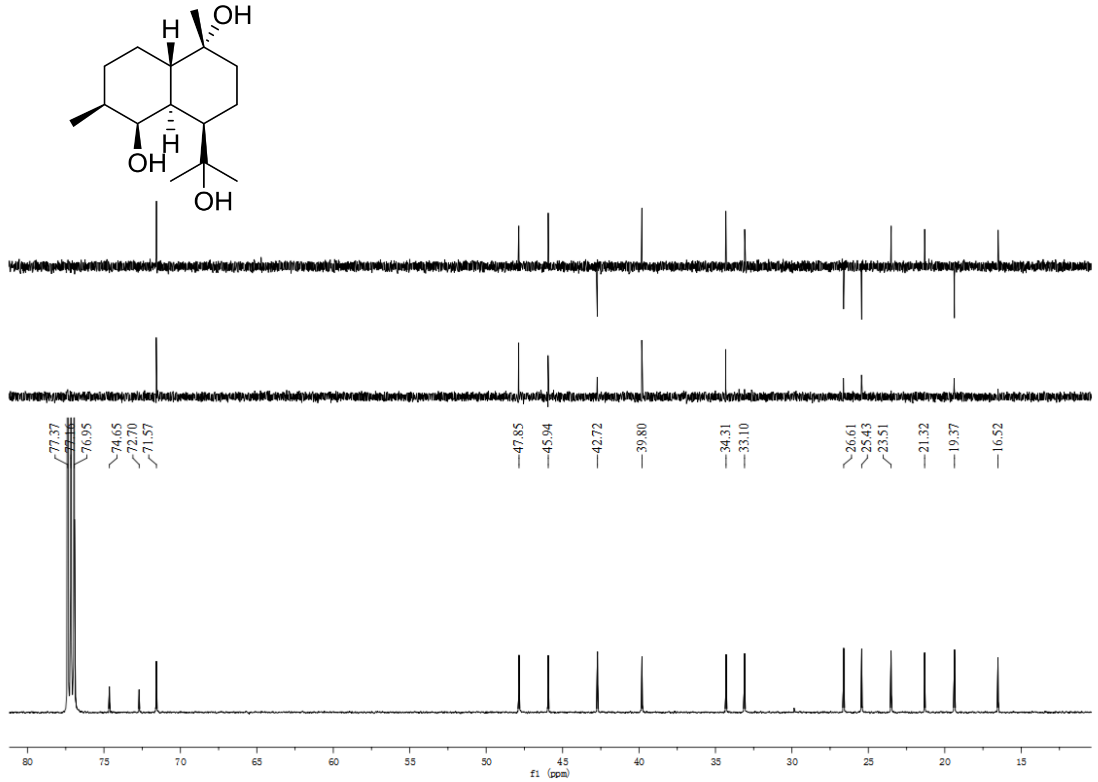

Figure S73. HSQC spectrum of compound **8** in $CDCl_3$

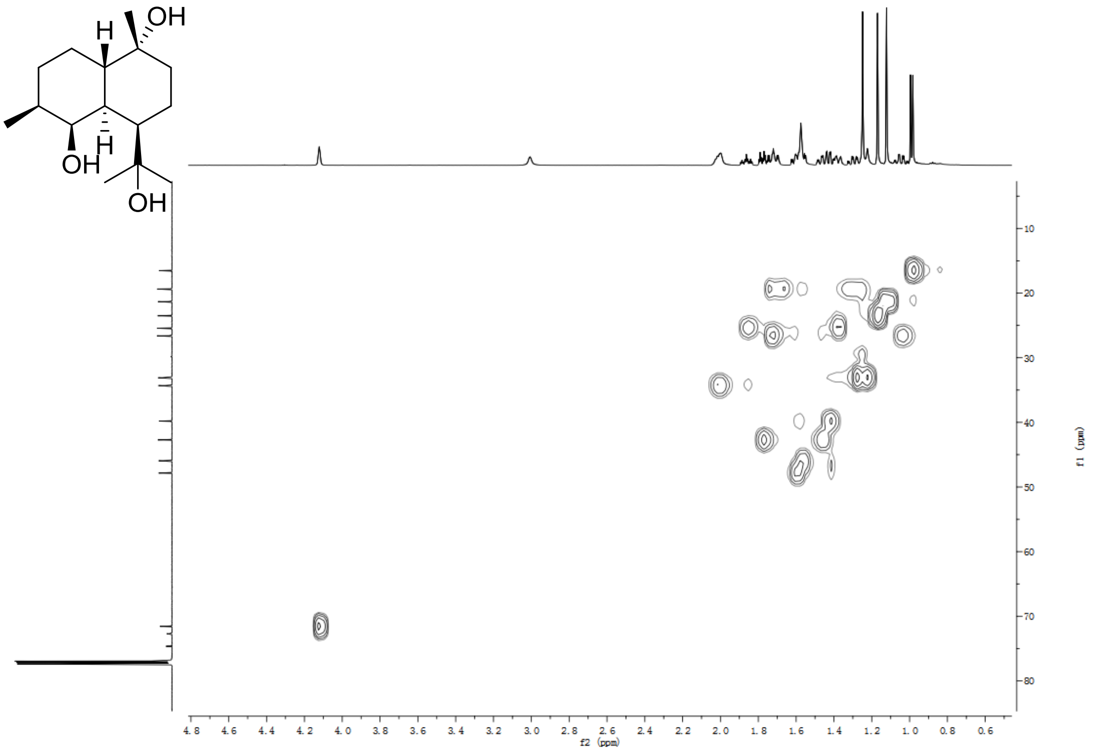

Figure S74. ^1H-^1H COSY spectrum of compound **8** in $CDCl_3$

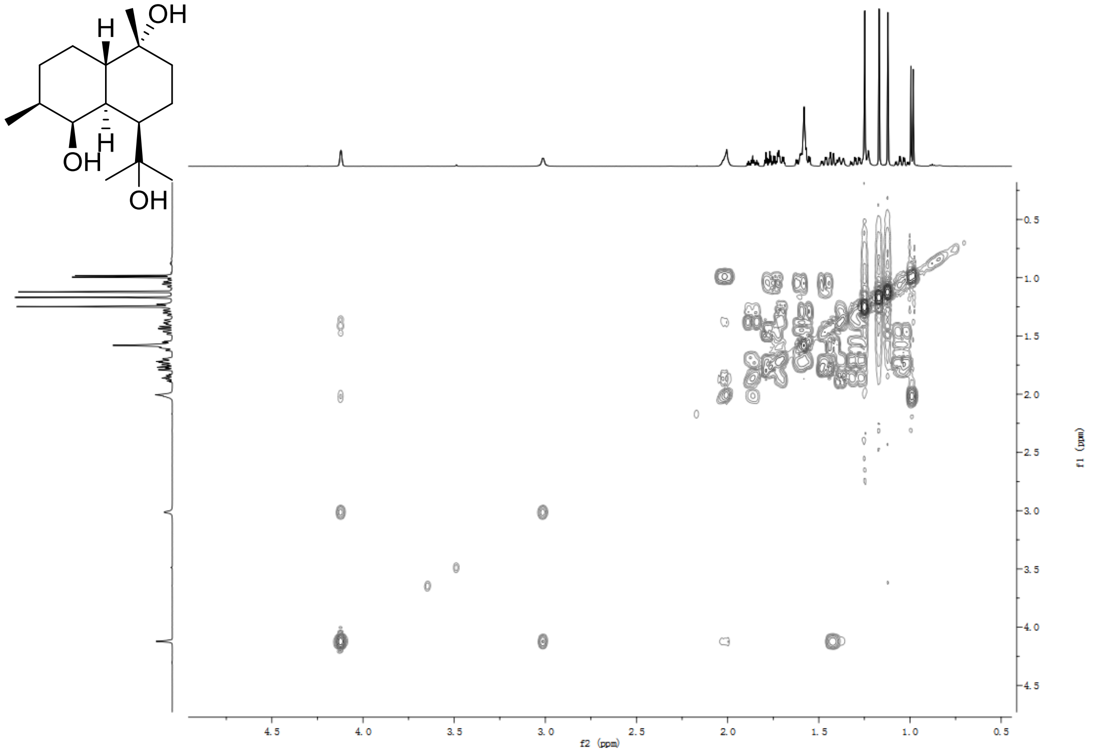

Figure S75. HMBC spectrum of compound **8** in $CDCl_3$

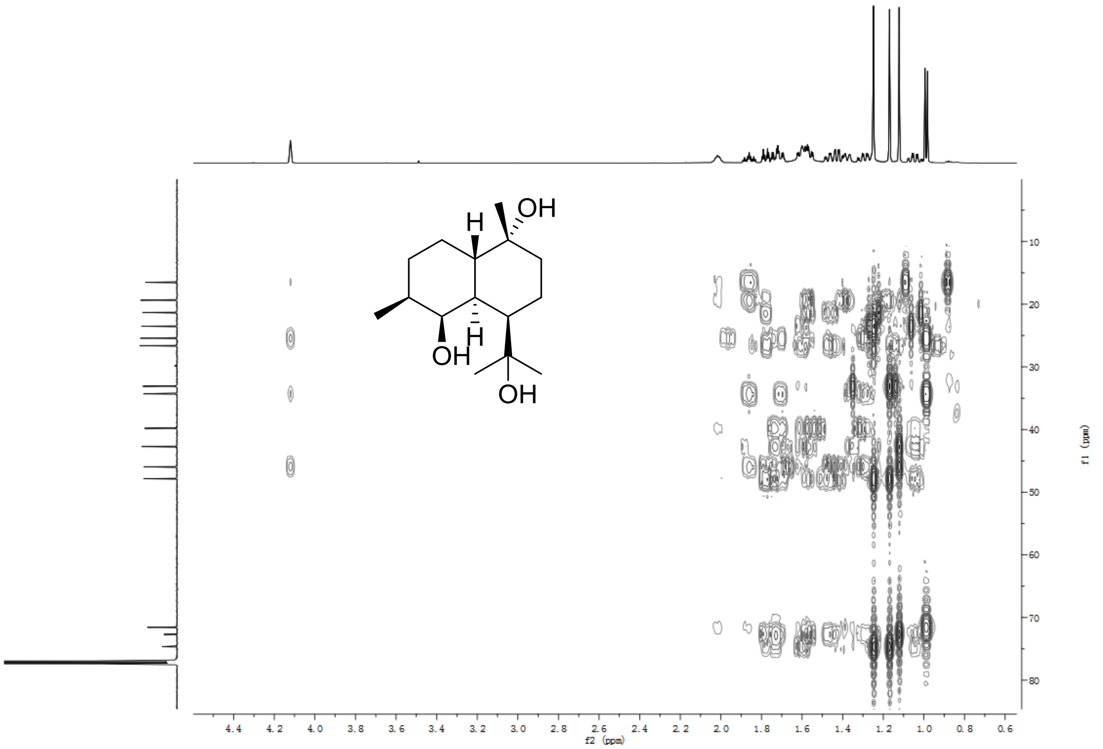

Figure S76. NOESY spectrum of compound **8** in $CDCl_3$

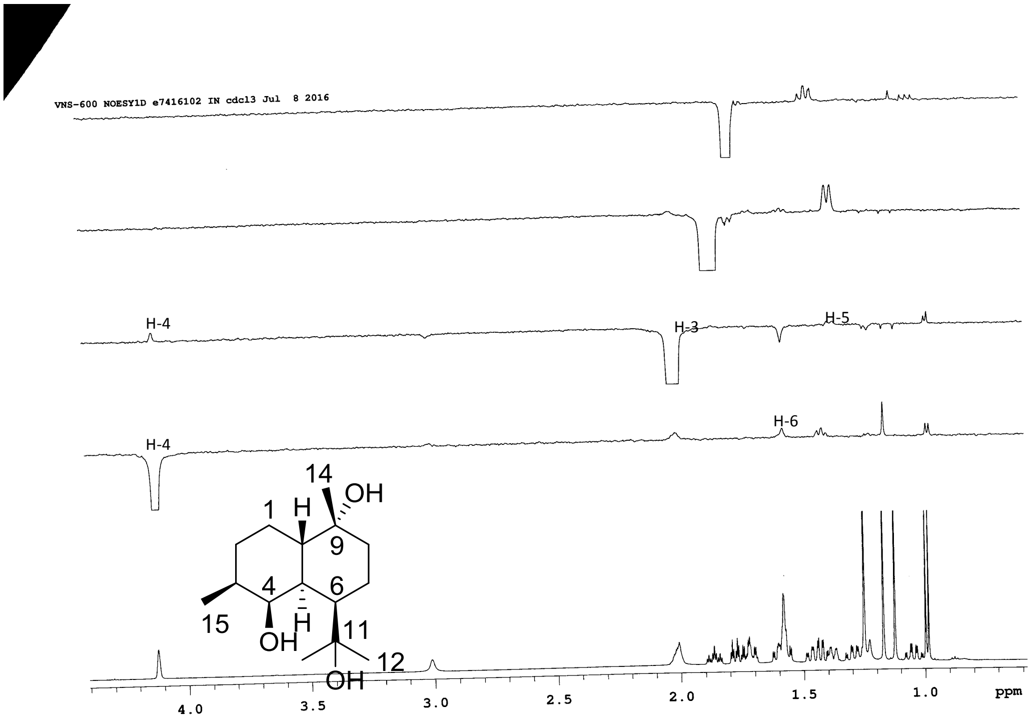

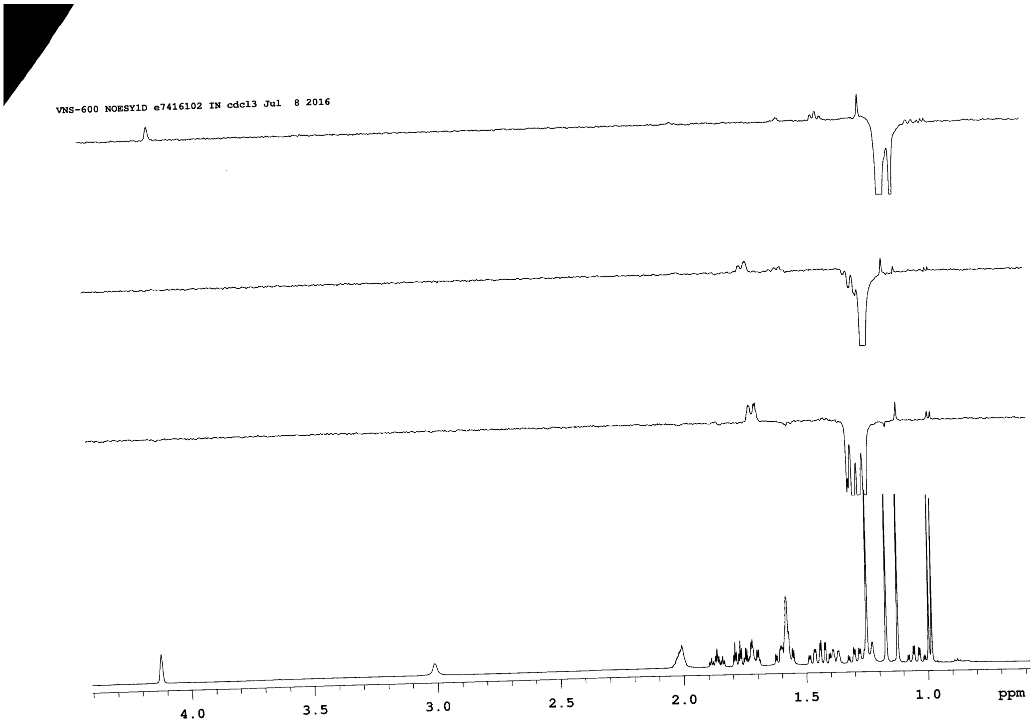
VNS-600 NOESY1D e7416102 IN cdcl3 Jul 8 2016
4.0
3.5
3.0
2.5
2.0
1.5
1.0
ppm

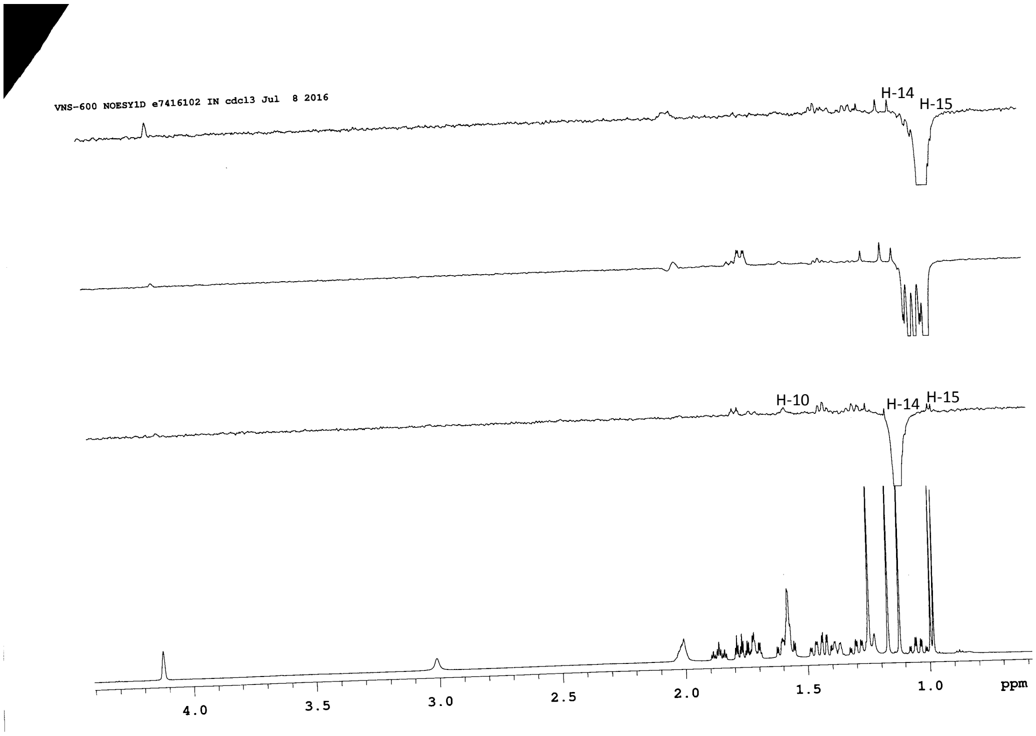
VNS-600 NOESY1D e7416102 IN cdcl3 Jul 8 2016
H-14
H-15
H-10
H-14
H-15
4.0
3.5
3.0
2.5
2.0
1.5
1.0
ppm

Figure S77. HRESIMS spectrum of compound **8**

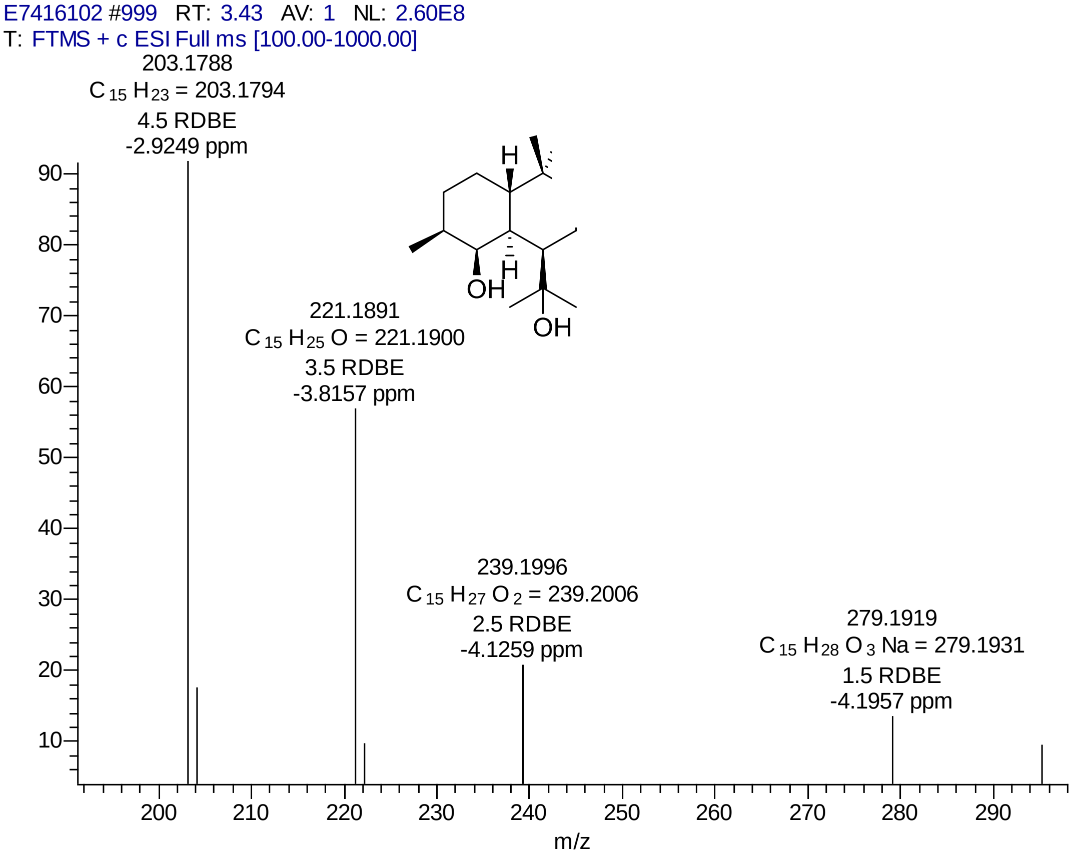

Figure S78. IR spectrum of compound **8**

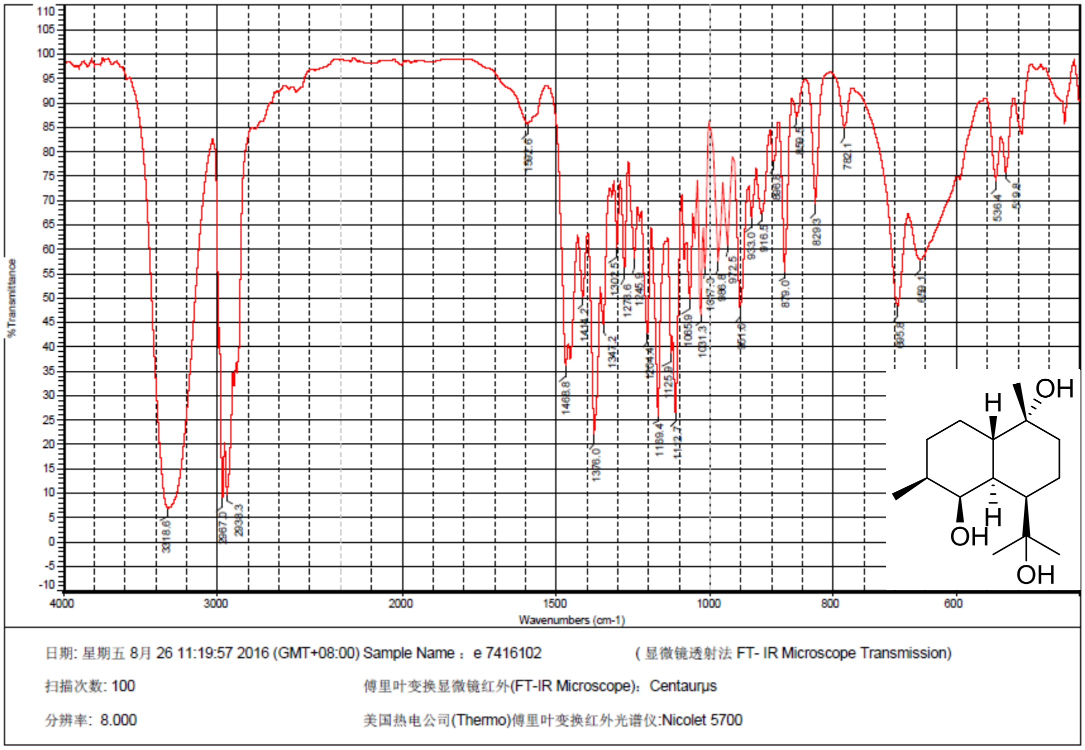

Figure S79. ^{1}H NMR spectrum of compound **9** in $CDCl_3$

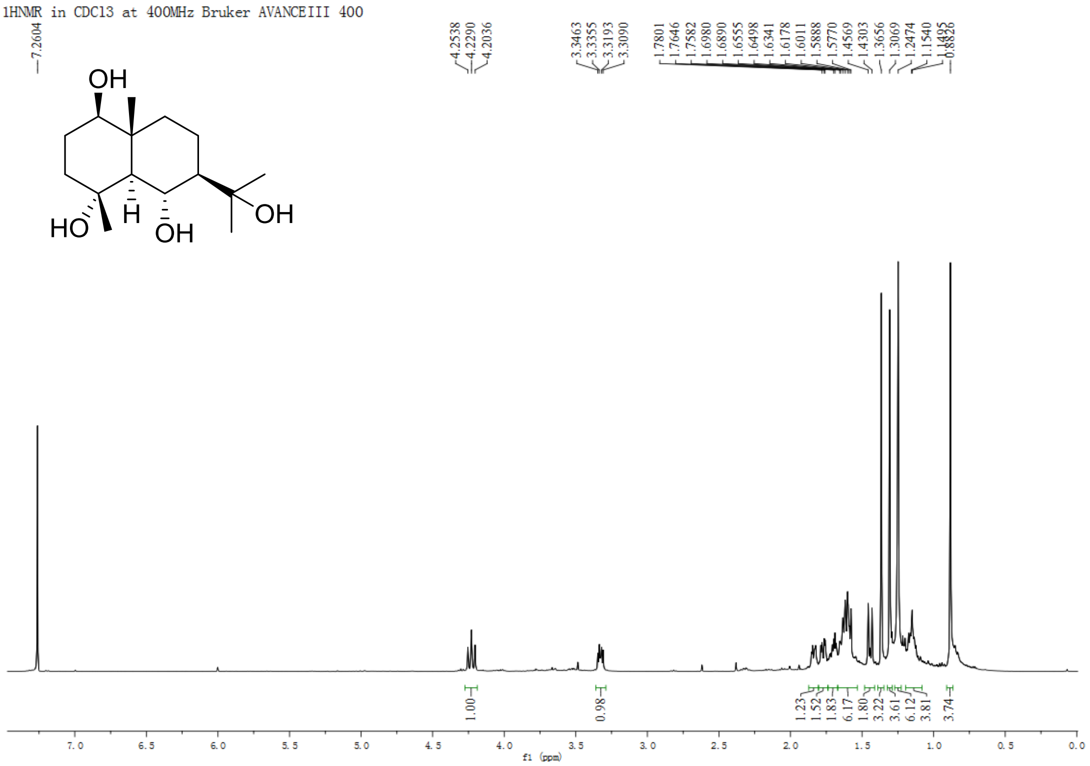

Figure S80. ^{13}C NMR spectrum of compound **9** in $CDCl_3$

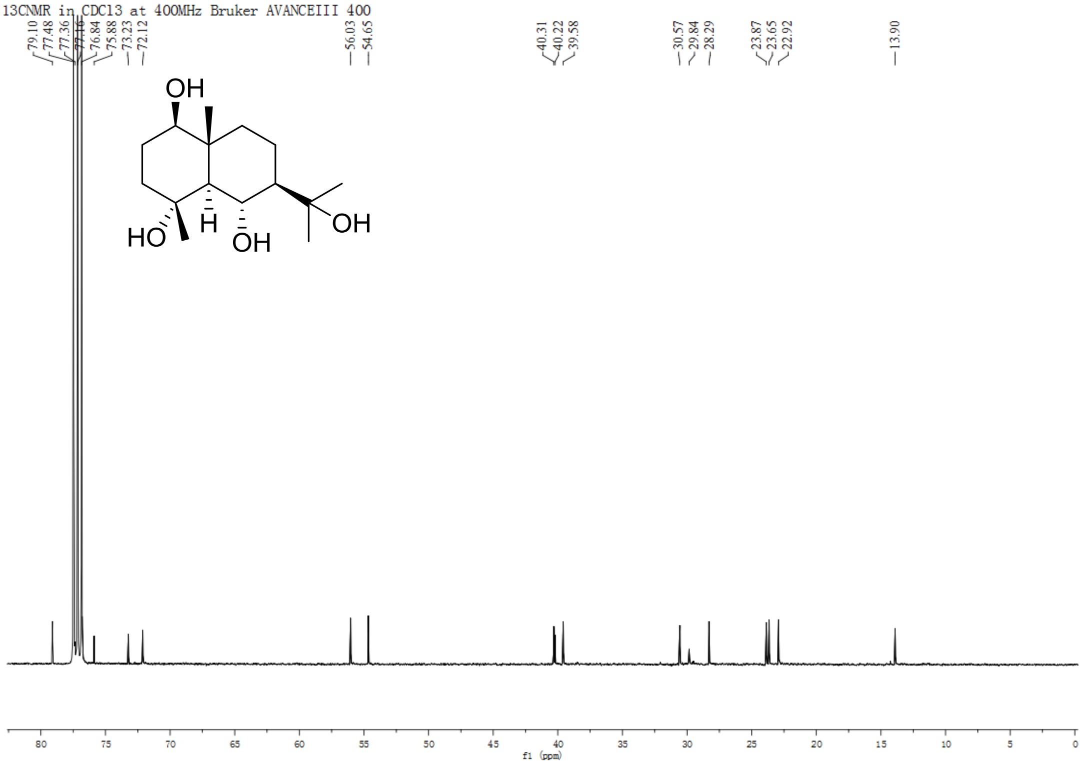

Figure S81. DEPT spectrum of compound **9** in $CDCl_3$

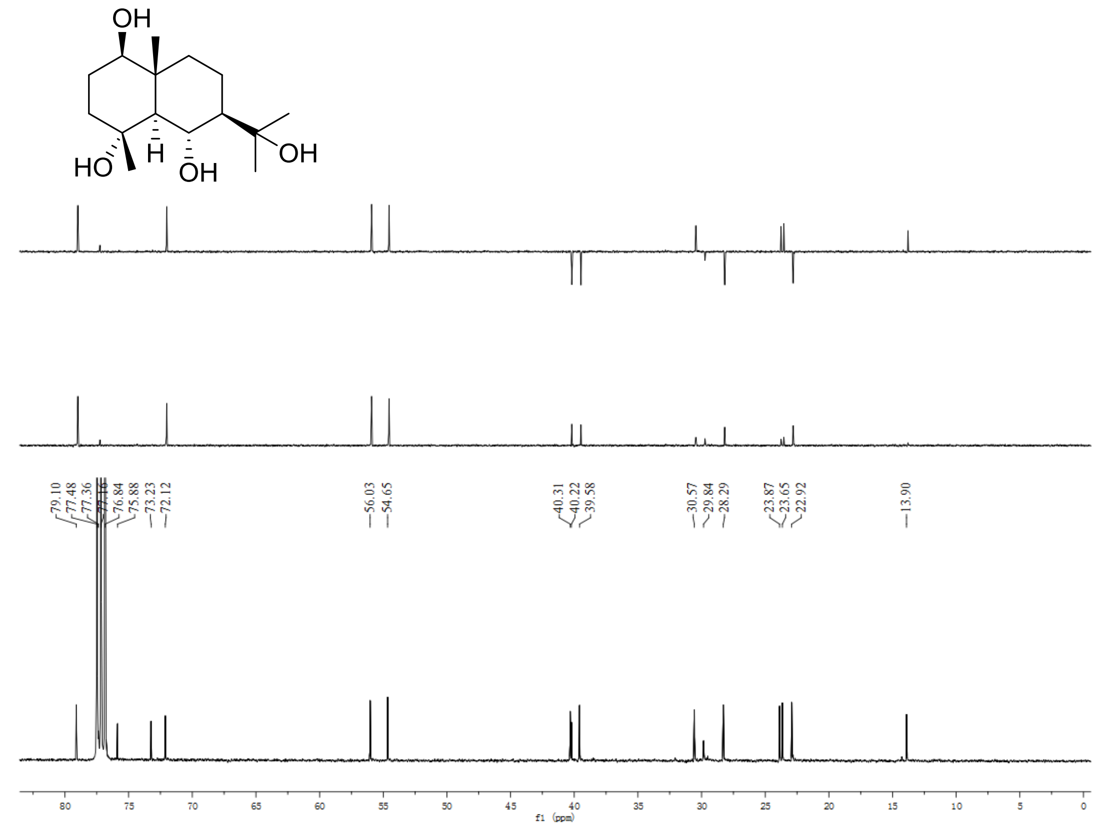

Figure S82. HSQC spectrum of compound **9** in $CDCl_3$

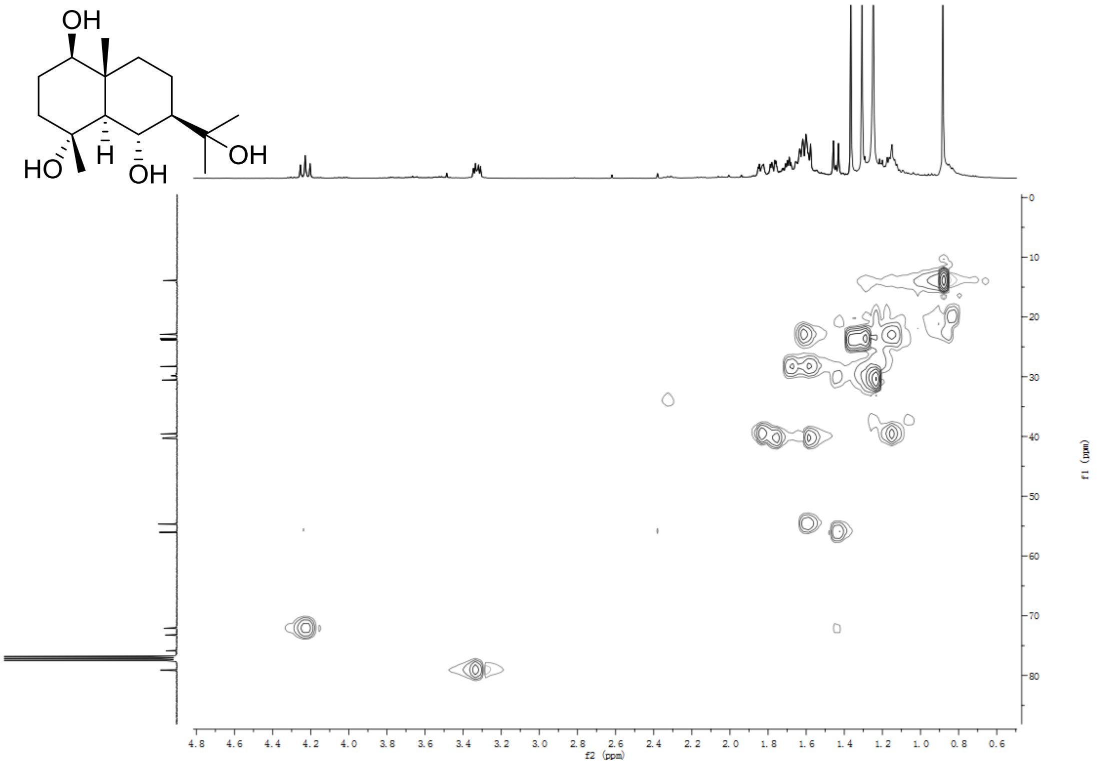

Figure S83. ^{1}H-^{1}H COSY spectrum of compound **9** in $CDCl_3$

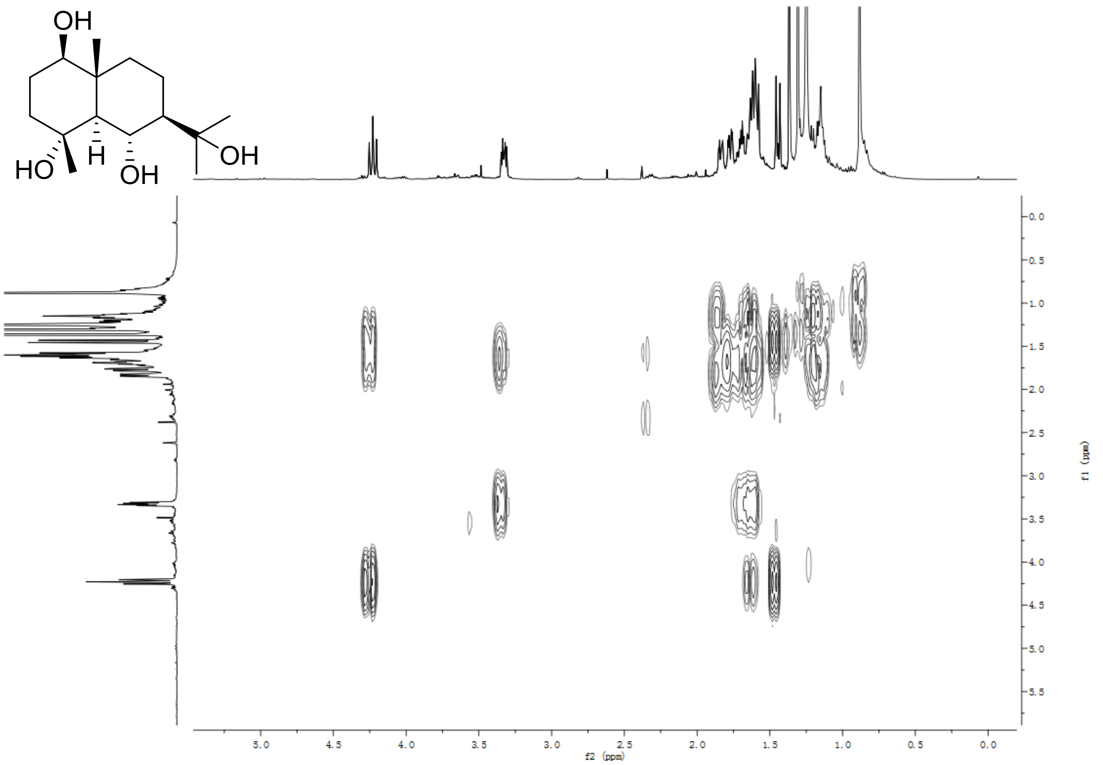

Figure S84. HMBC spectrum of compound **9** in $CDCl_3$

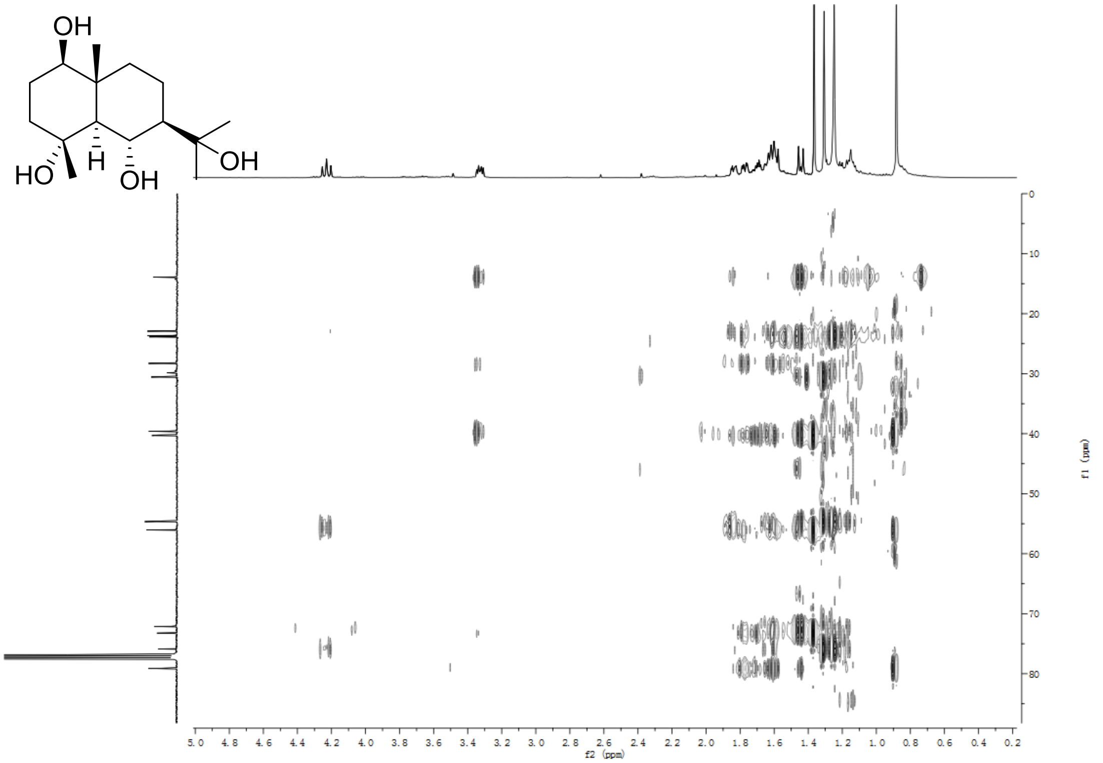

Figure S85. NOESY spectrum of compound **9** in $CDCl_3$

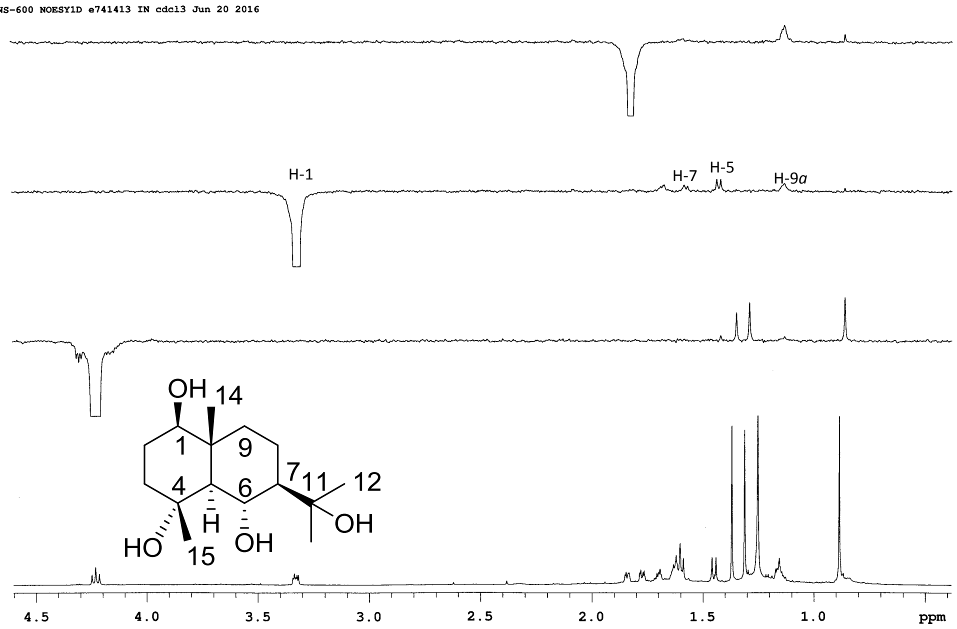

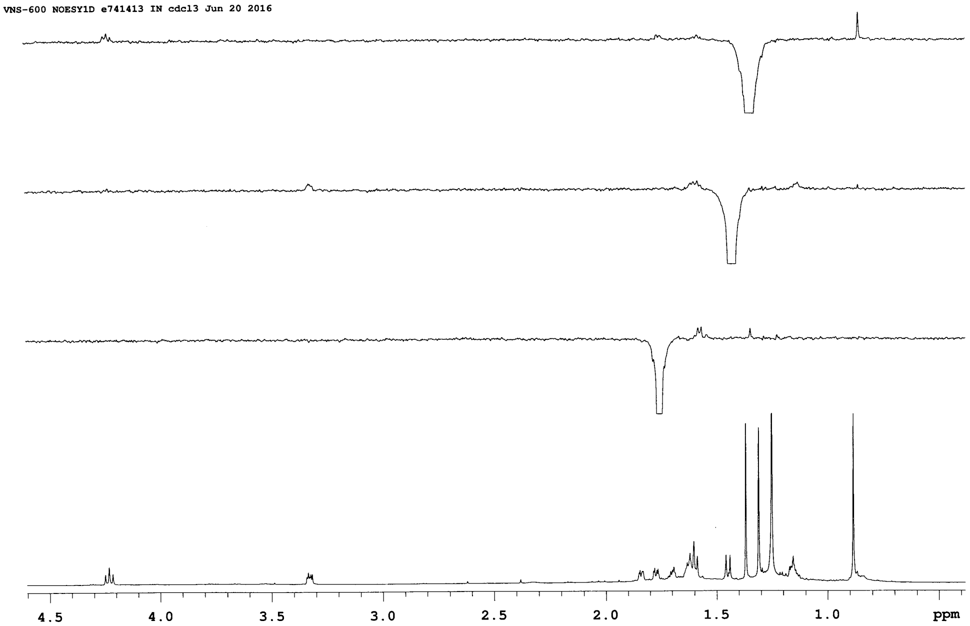

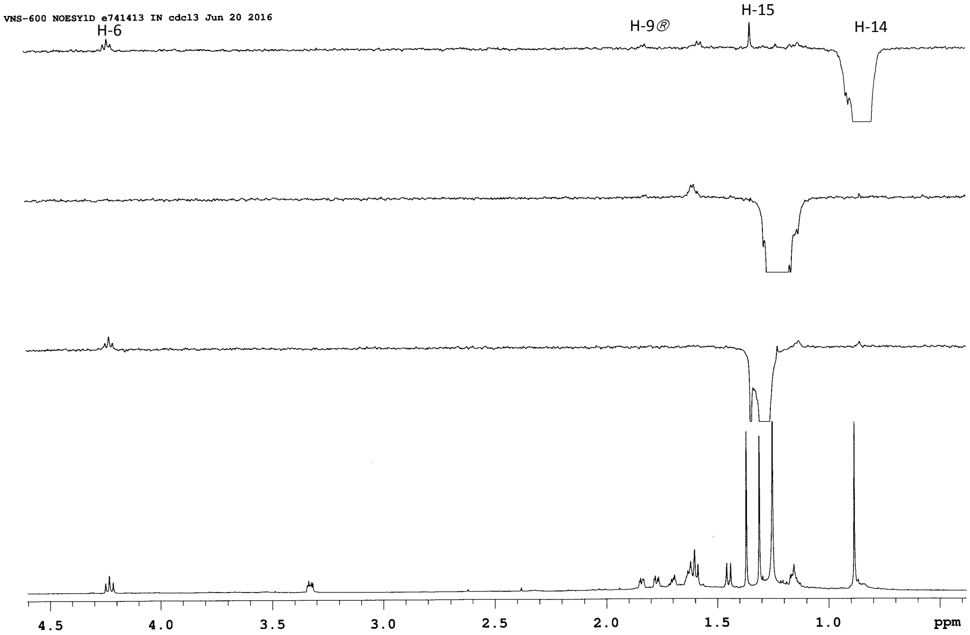

Figure S86. HRESIMS spectrum of compound **9**

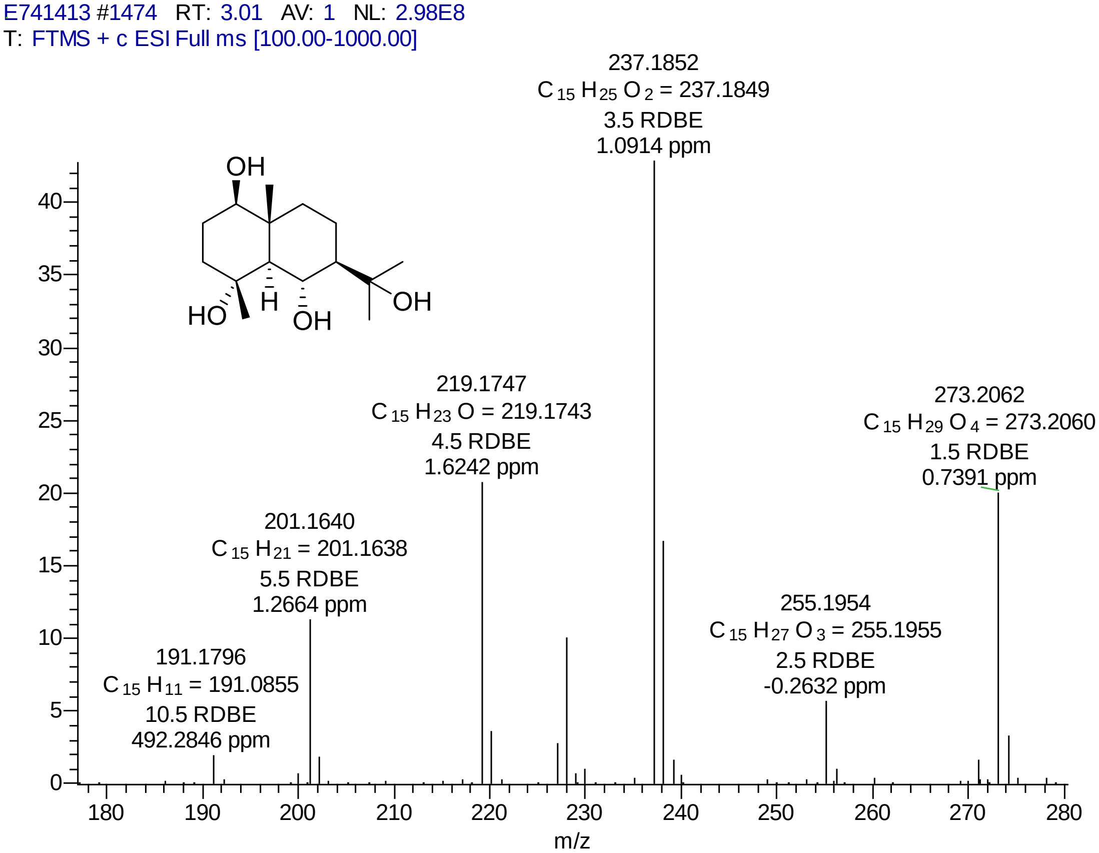

Figure S87. IR spectrum of compound **9**

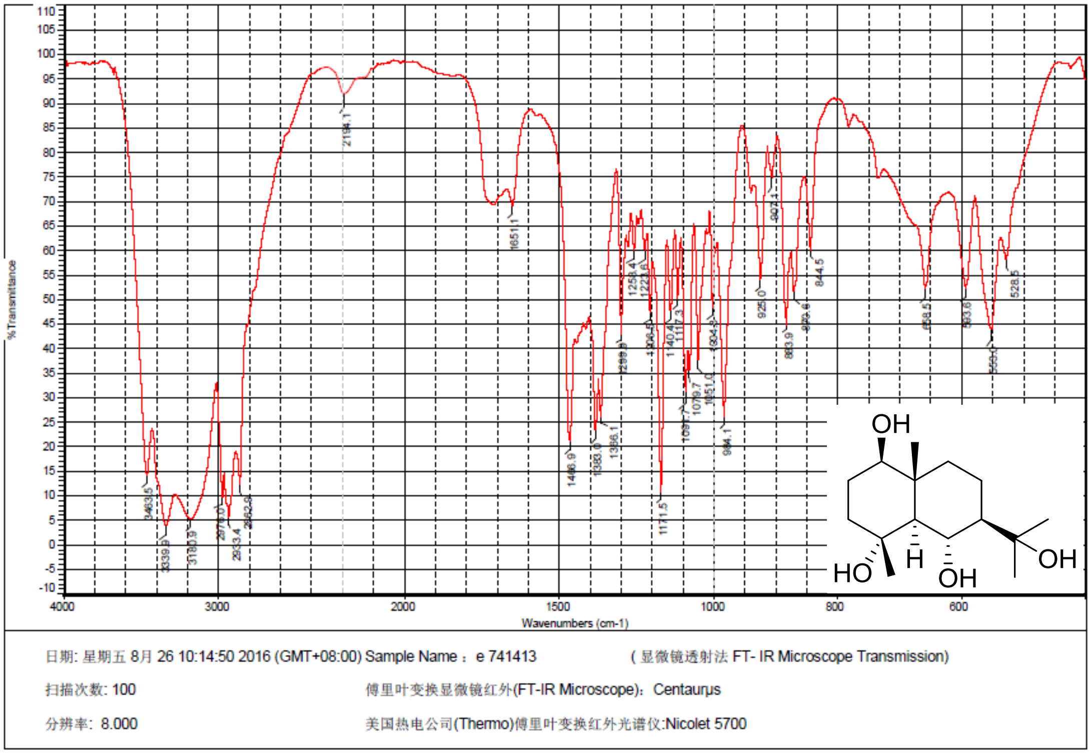

Figure S88. ^{1}H NMR spectrum of compound **10** in $CDCl_3$

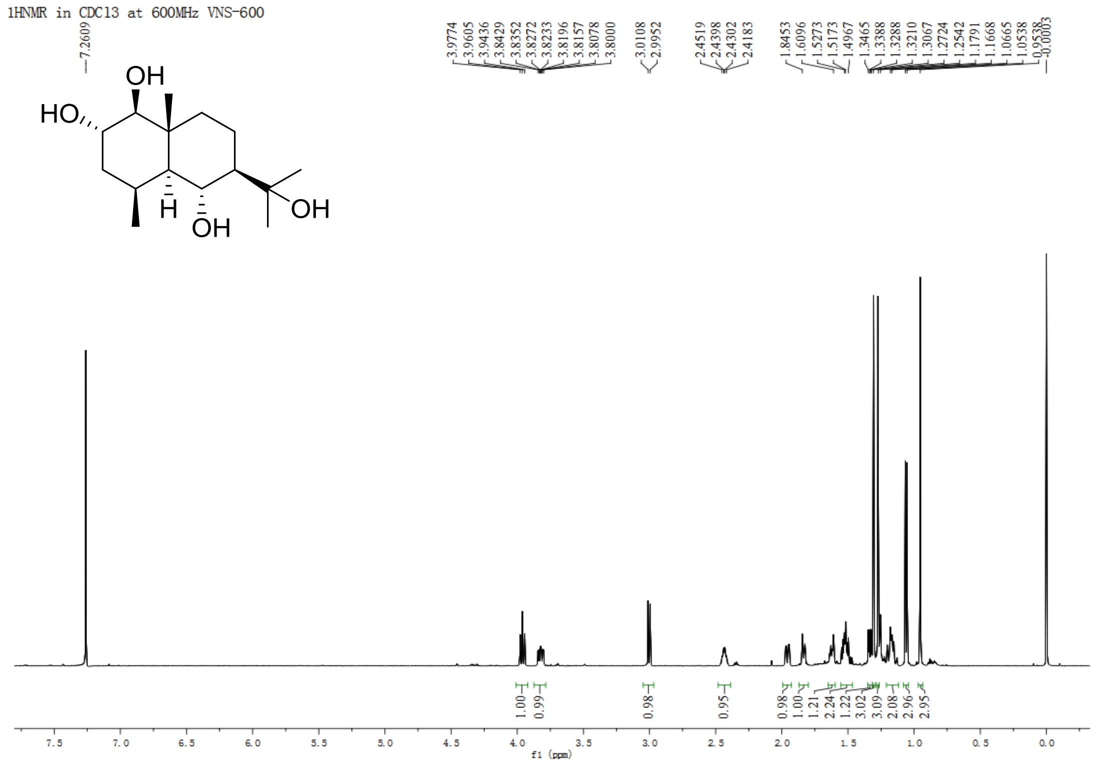

Figure S89. ^{13}C NMR spectrum of compound **10** in $CDCl_3$

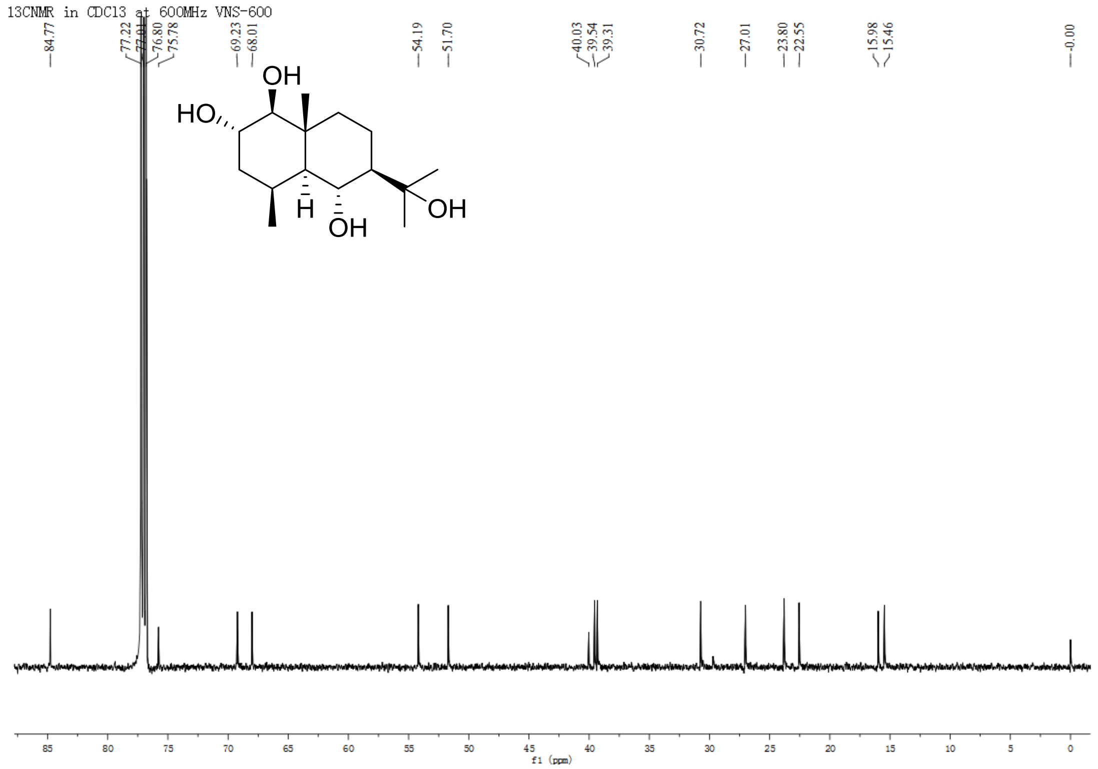

Figure S90. DEPT spectrum of compound **10** in $CDCl_3$

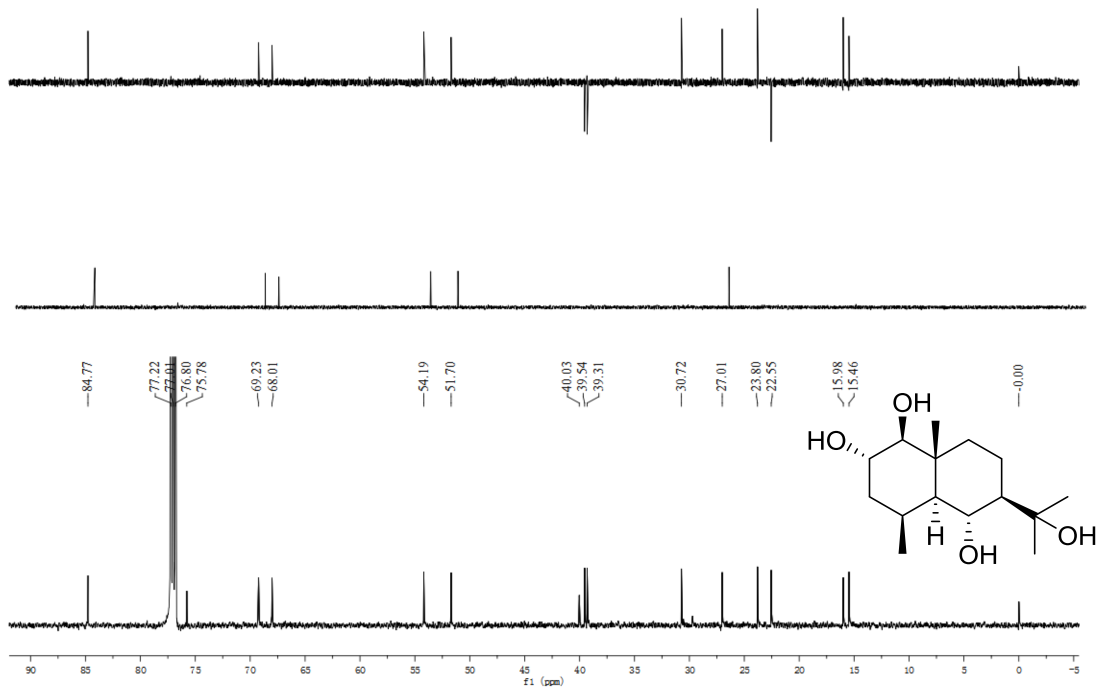

Figure S91. HSQC spectrum of compound **10** in $CDCl_3$

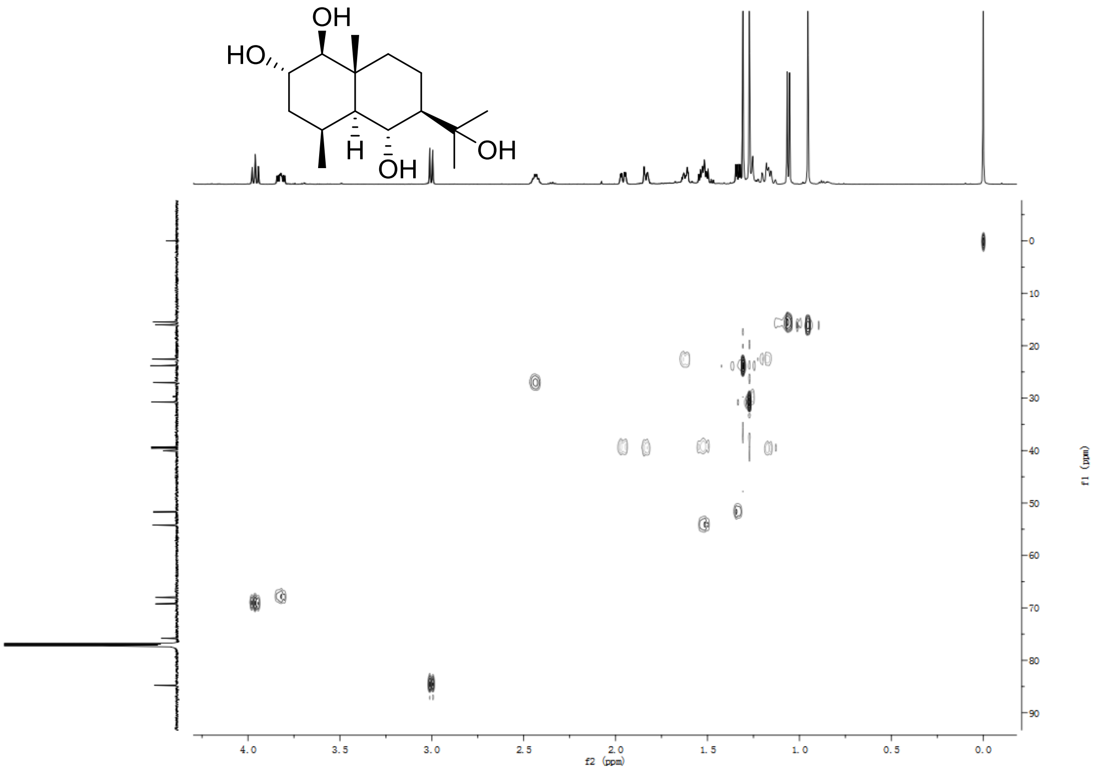

Figure S92. ^1H-^1H COSY spectrum of compound **10** in $CDCl_3$

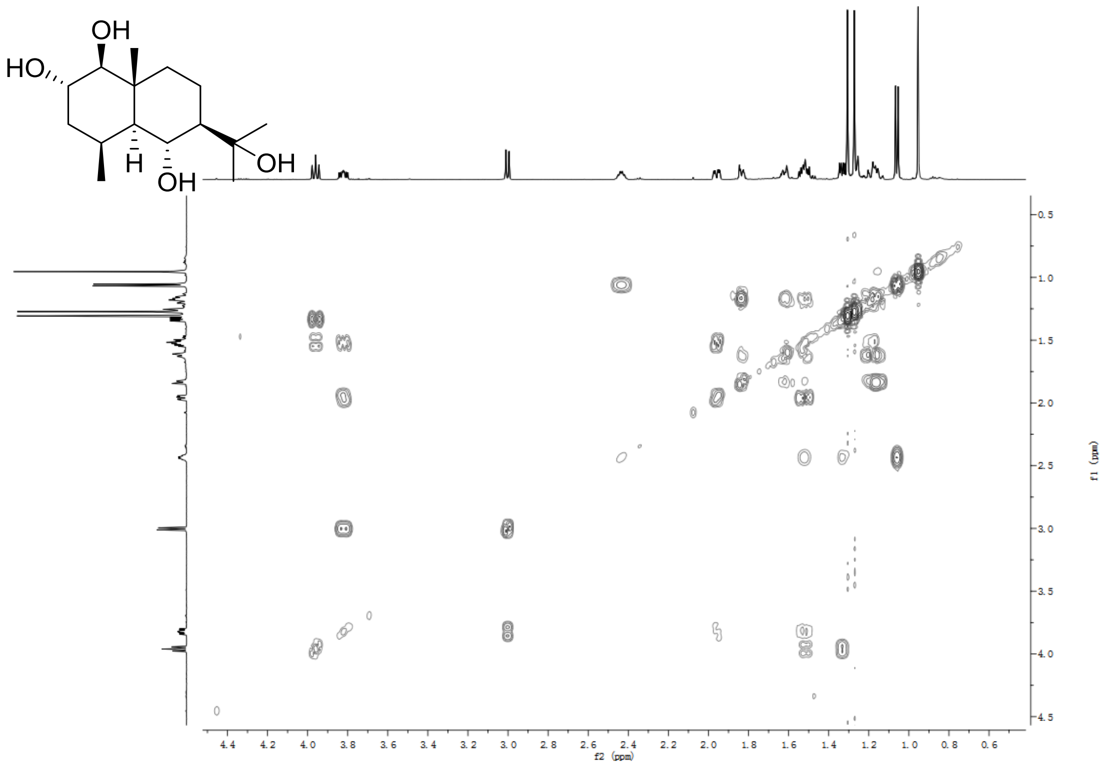

Figure S93. HMBC spectrum of compound **10** in $CDCl_3$

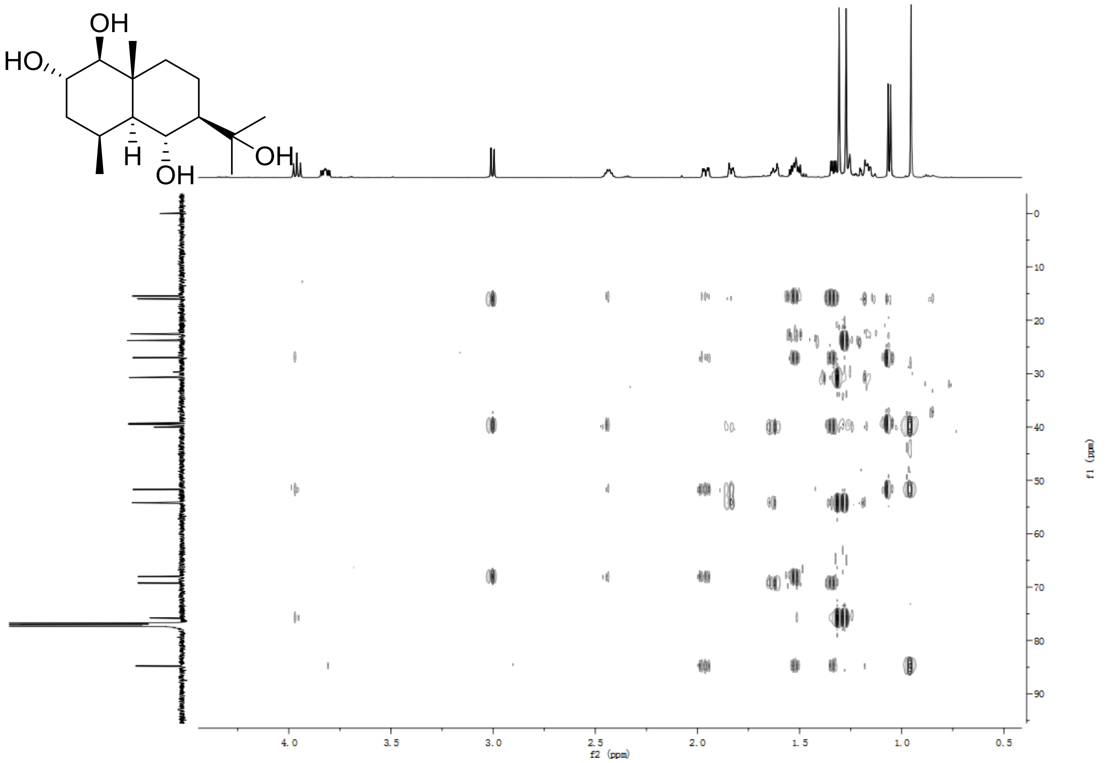

Figure S94. NOESY spectrum of compound **10** in $CDCl_3$

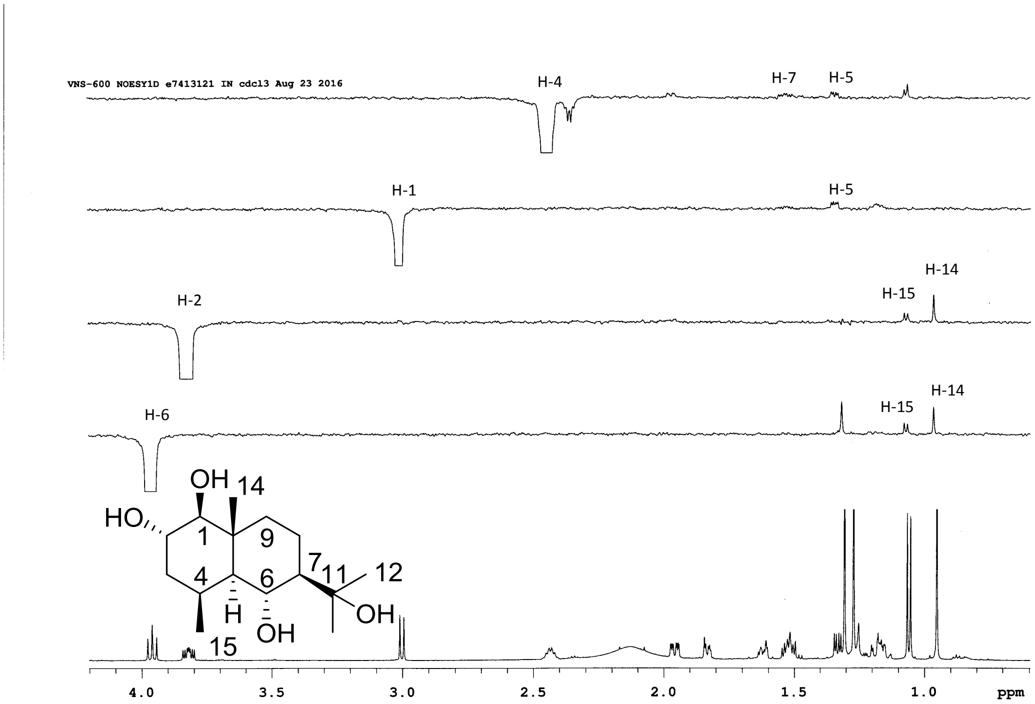

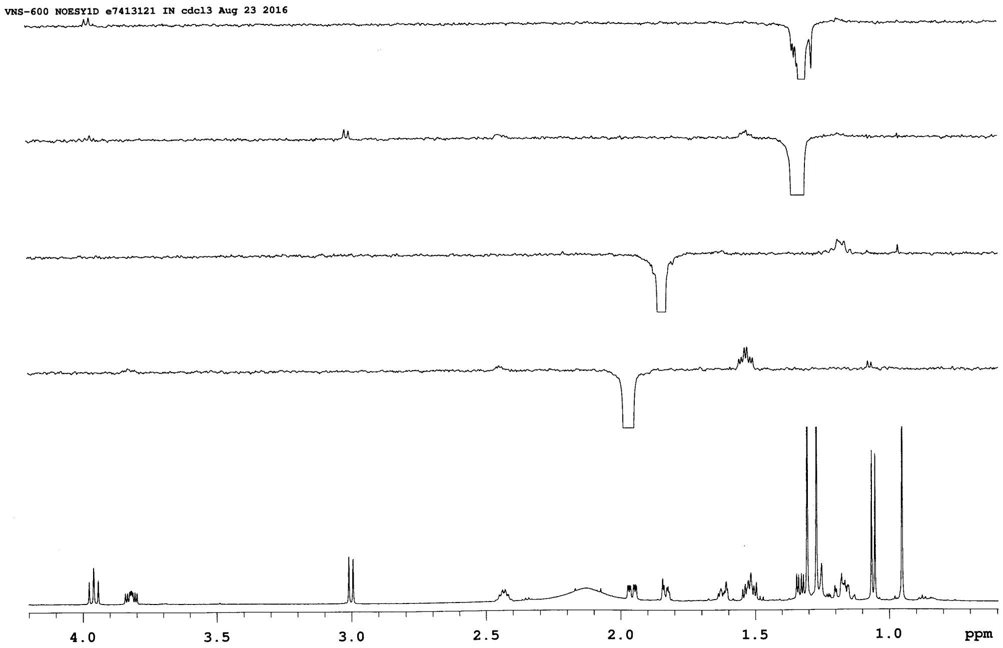
VNS-600 NOESY1D e7413121 IN cdcl3 Aug 23 2016
4.0
3.5
3.0
2.5
2.0
1.5
1.0
ppm

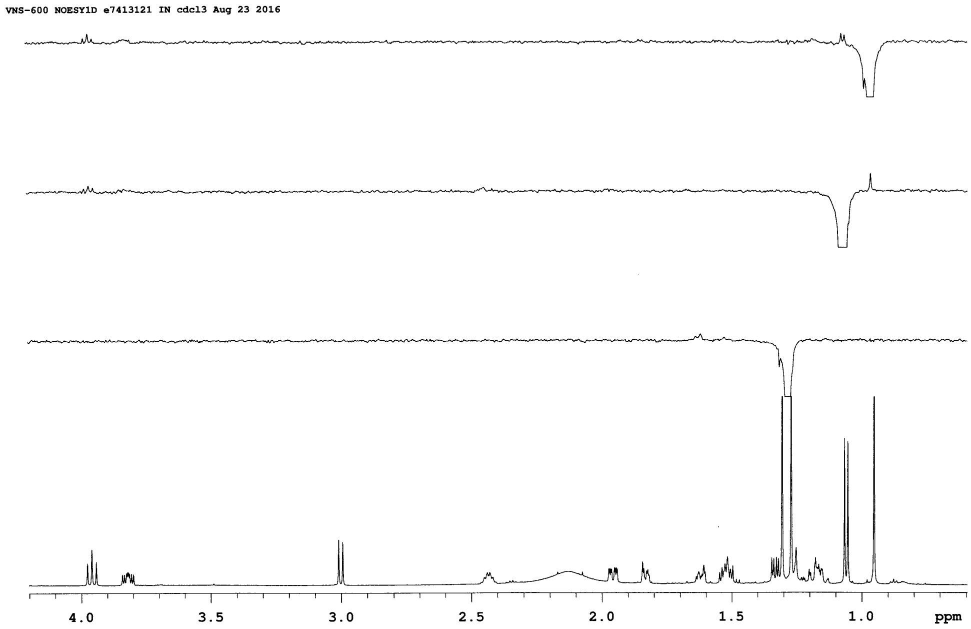
VNS-600 NOESY1D e7413121 IN cdcl3 Aug 23 2016
4.0
3.5
3.0
2.5
2.0
1.5
1.0
ppm

Figure S95. HRESIMS spectrum of compound **10**

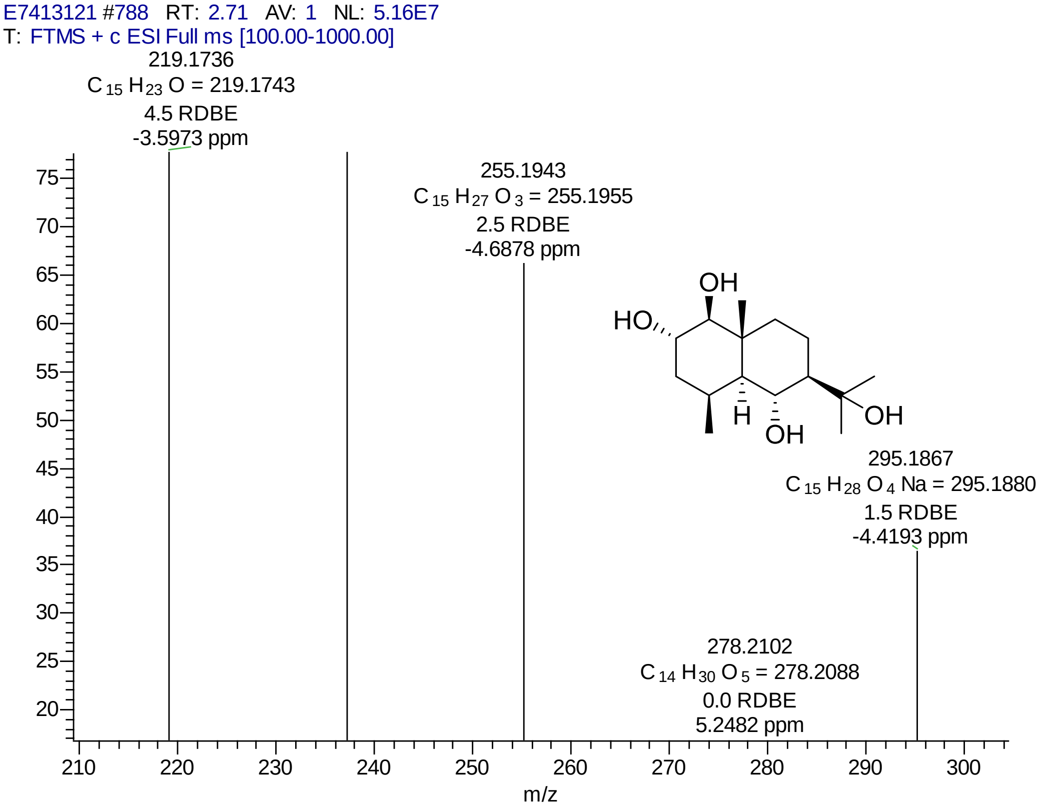

Figure S96. IR spectrum of compound **10**

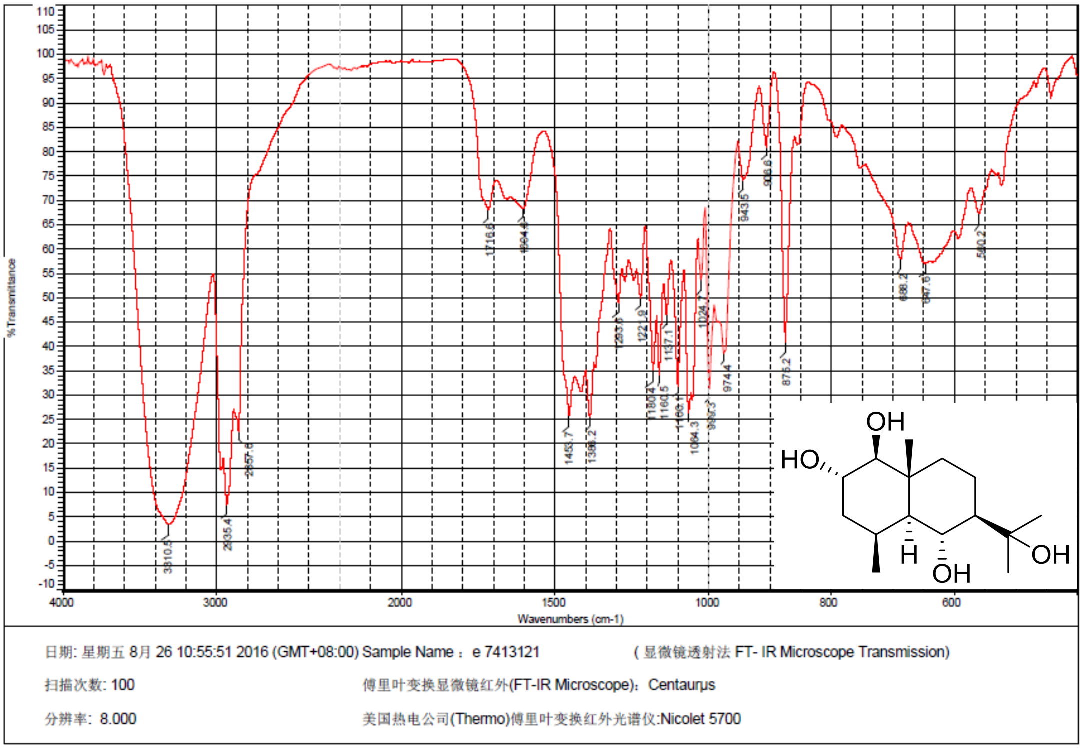

Figure S97. ^{1}H NMR spectrum of compound **11** in $CDCl_3$

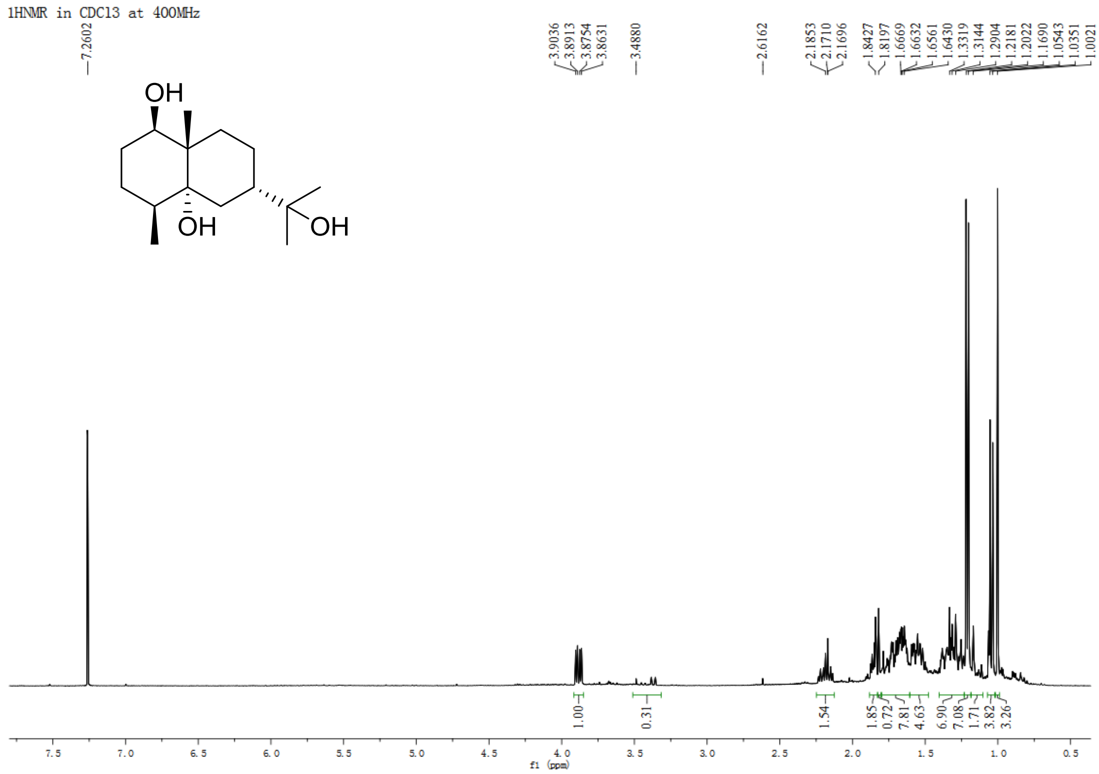

Figure S98. ^{13}C NMR spectrum of compound **11** in $CDCl_3$

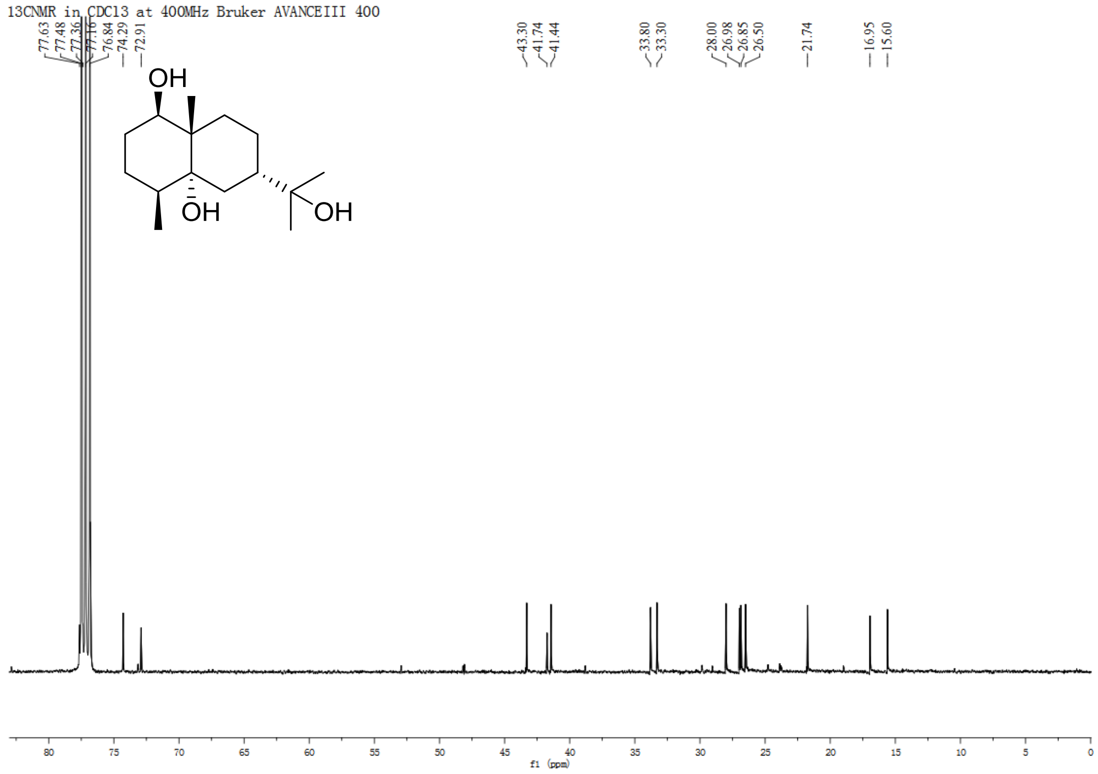

Figure S98-2. Enlarge ^{13}C NMR spectrum of compound **11** in $CDCl_3$

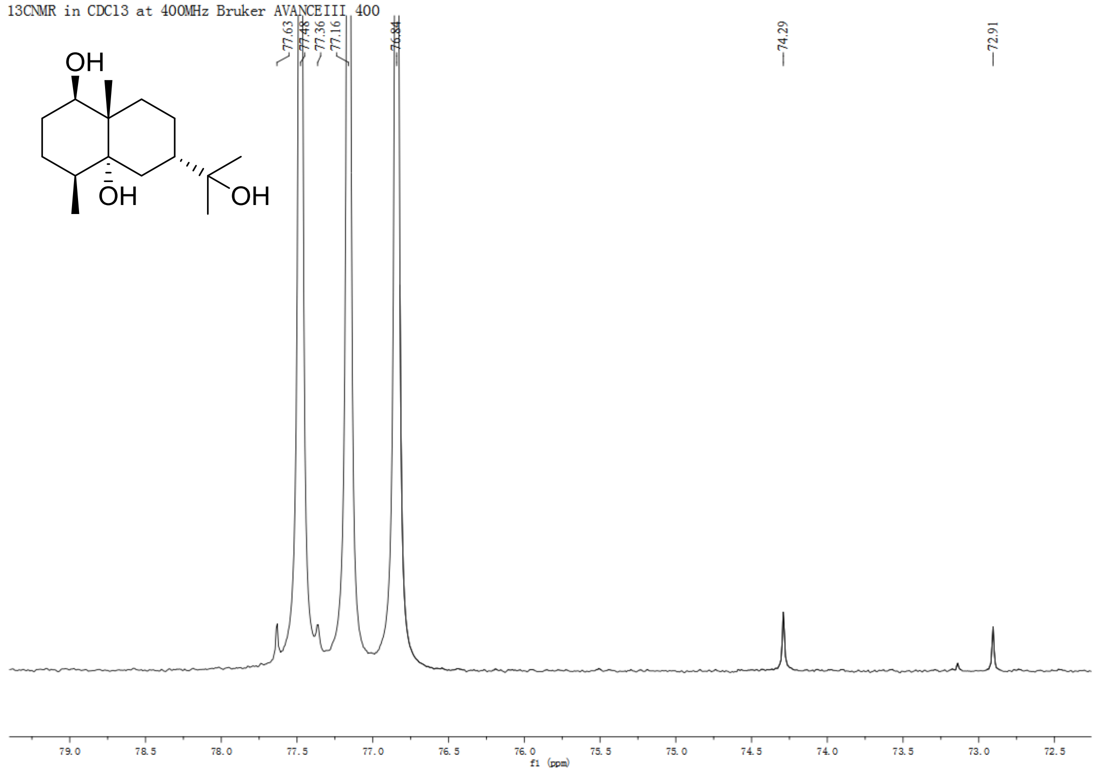

Figure S99. DEPT spectrum of compound **11** in $CDCl_3$

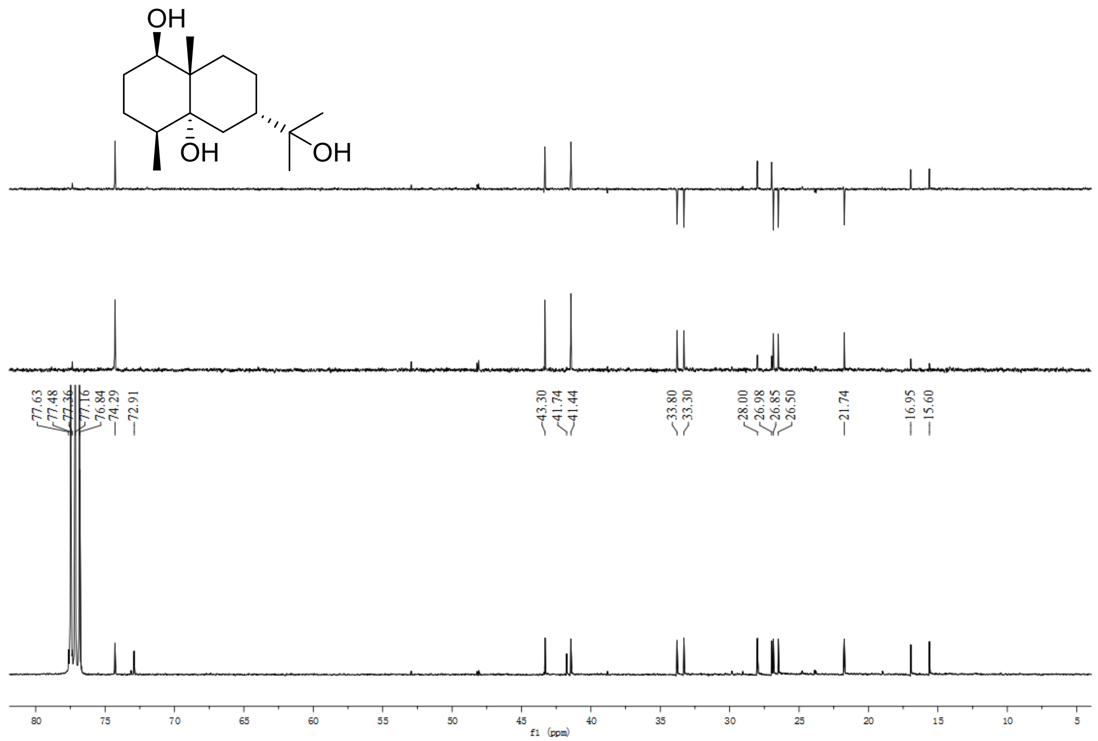

Figure S100. HSQC spectrum of compound **11** in $CDCl_3$

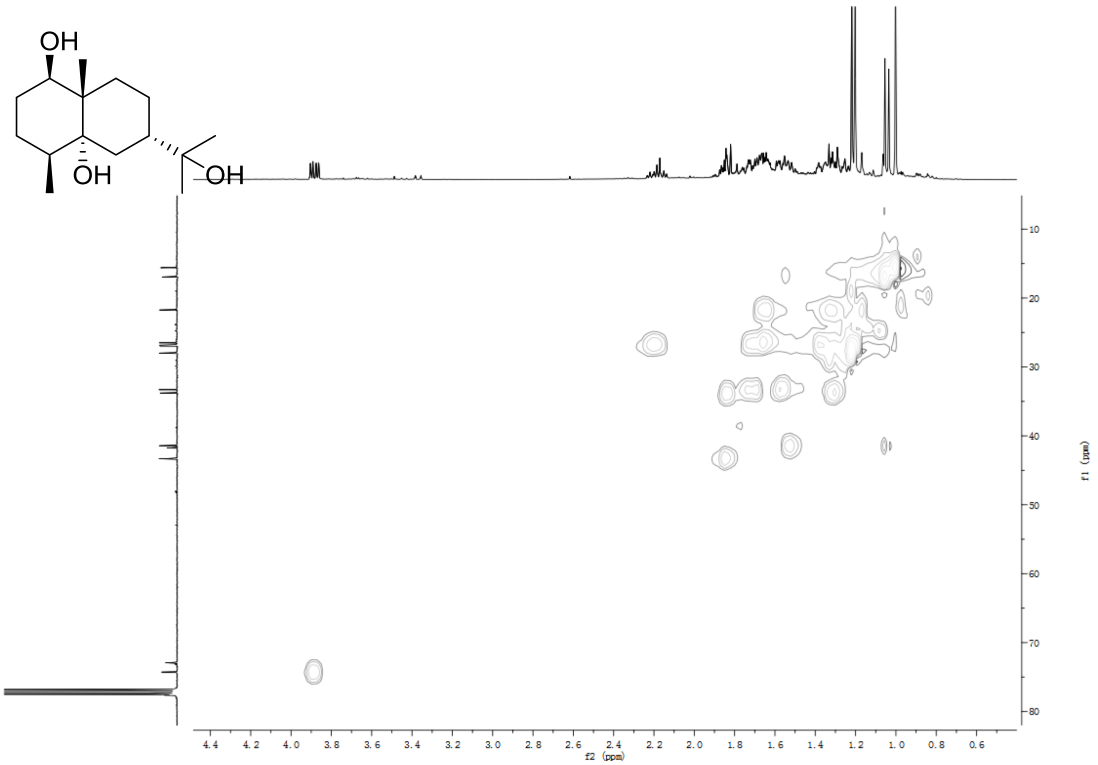

Figure S101. ^1H-^1H COSY spectrum of compound **11** in $CDCl_3$

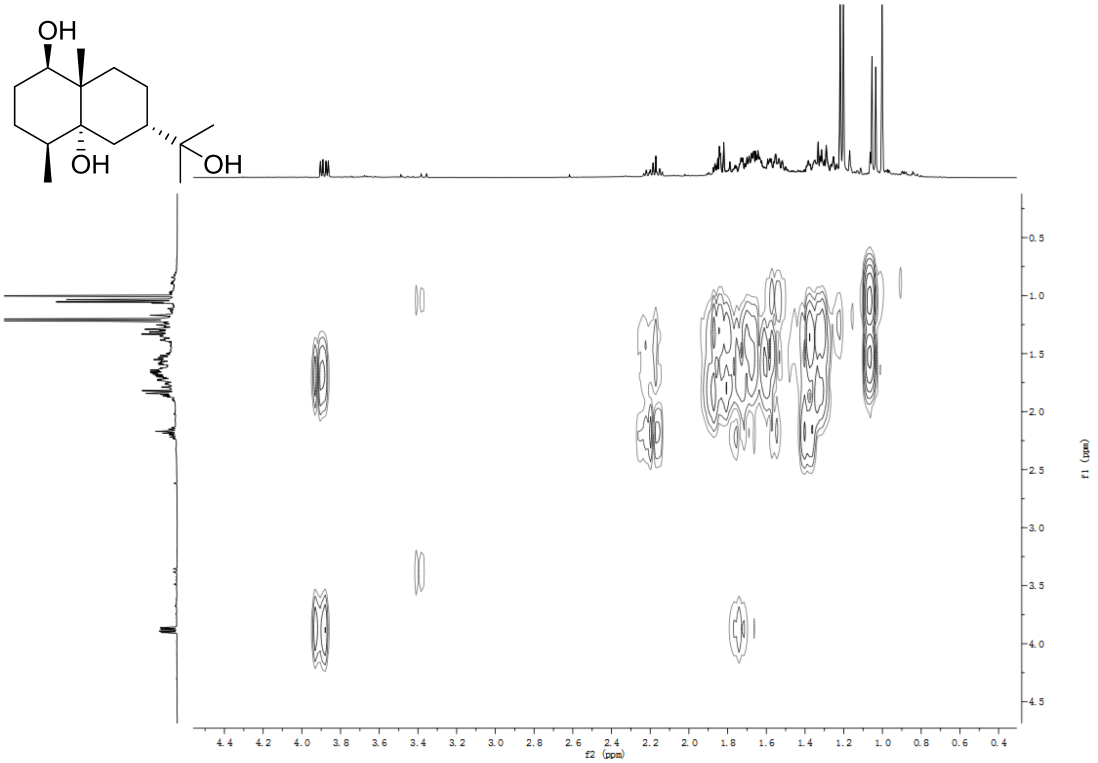

Figure S102. HMBC spectrum of compound **11** in $CDCl_3$

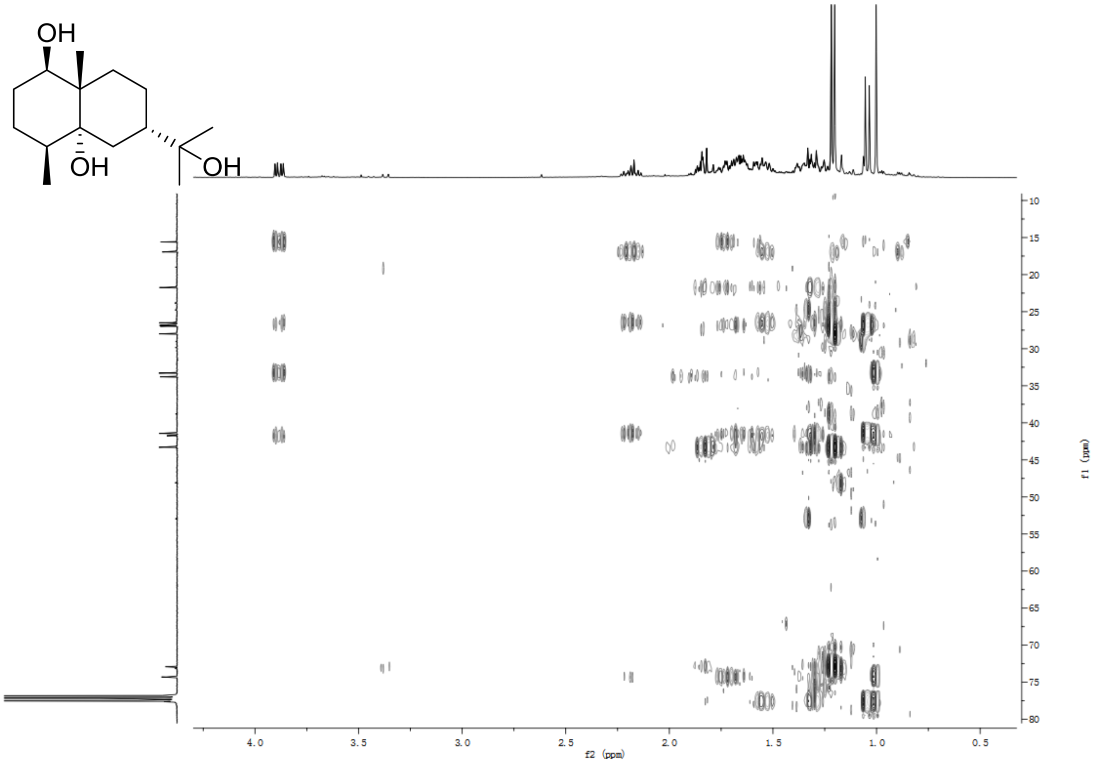

Figure S103. NOESY spectrum of compound **11** in $CDCl_3$

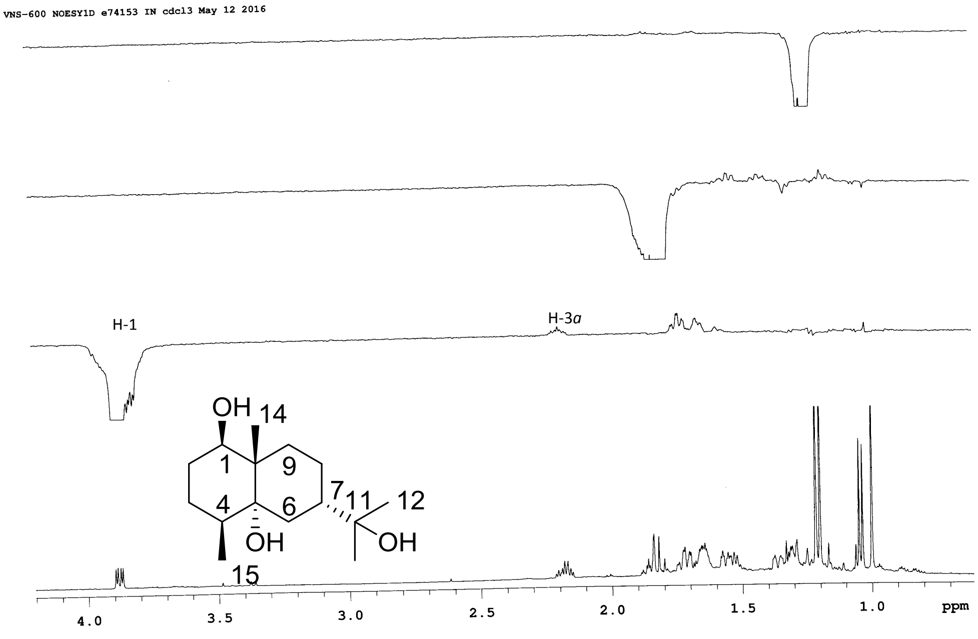

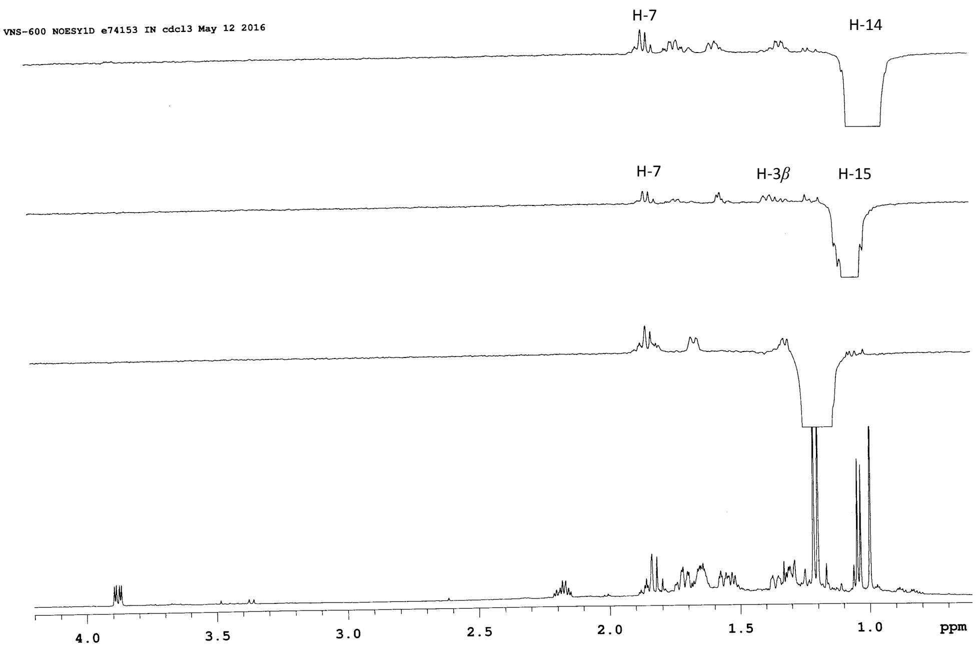
VNS-600 NOESY1D e74153 IN cdcl3 May 12 2016
H-7
H-14
H-7
H-3β
H-15
4.0
3.5
3.0
2.5
2.0
1.5
1.0
ppm

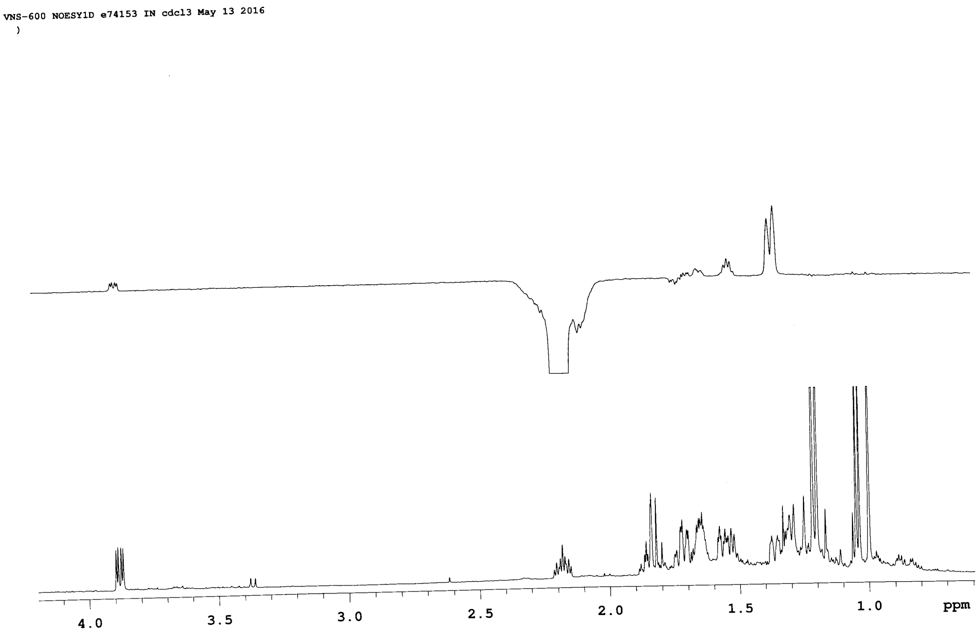
VNS-600 NOESY1D e74153 IN cdcl3 May 13 2016
4.0
3.5
3.0
2.5
2.0
1.5
1.0
ppm

Figure S104. HRESIMS spectrum of compound **11**

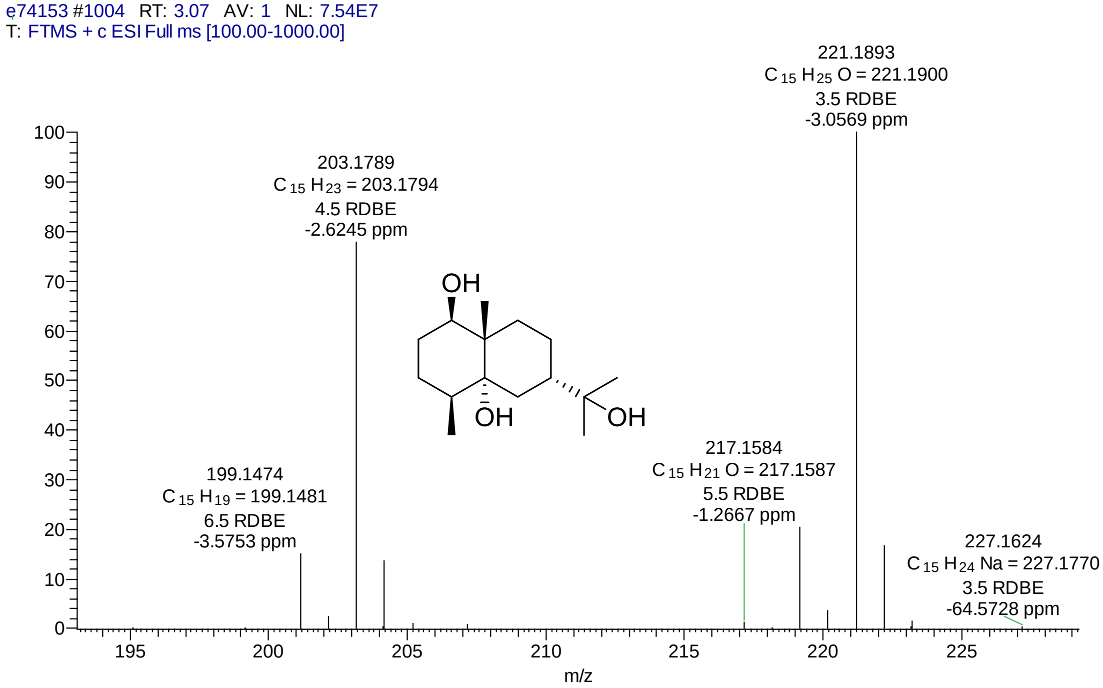

Figure S105. IR spectrum of compound **11**

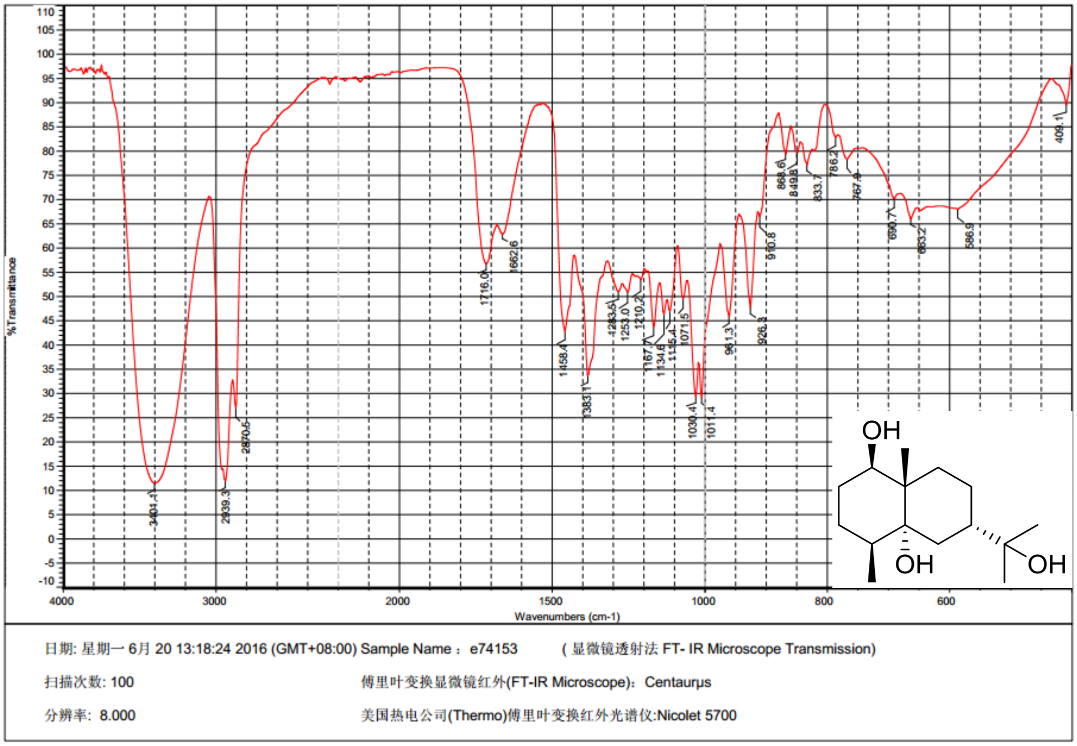

Figure S106. ^{1}H NMR spectrum of compound **11** in DMSO-d_6

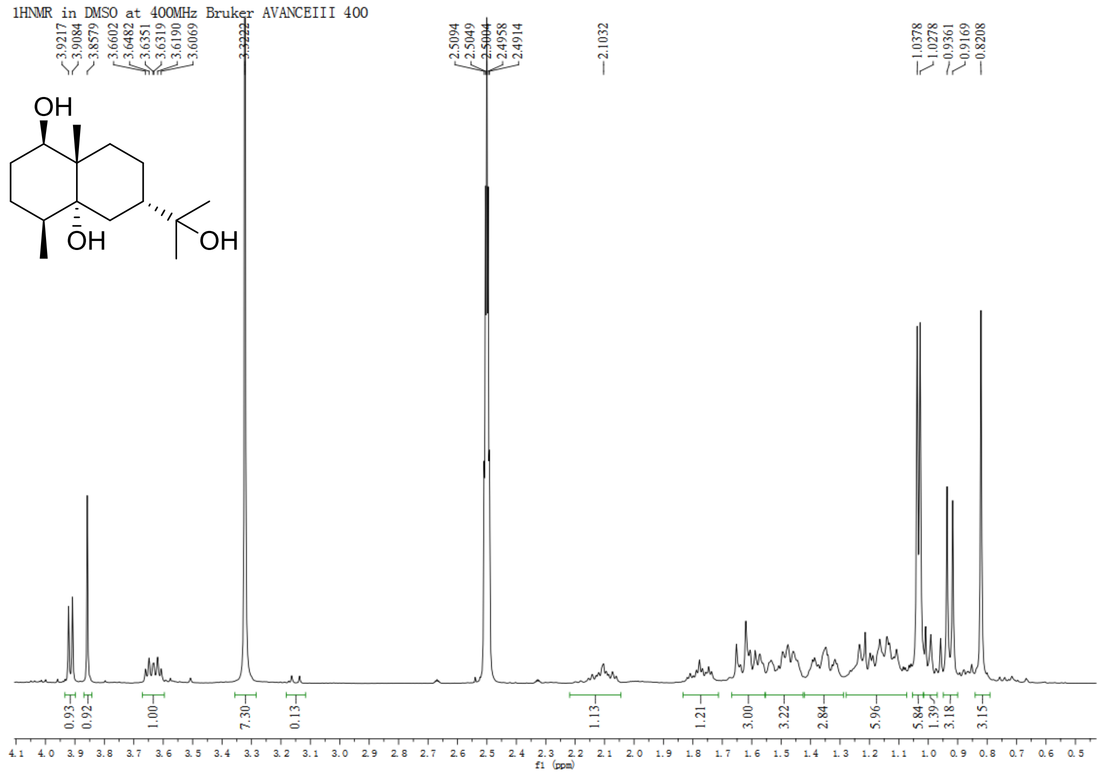

Figure S107. ^{13}C NMR spectrum of compound **11** in DMSO-d_6

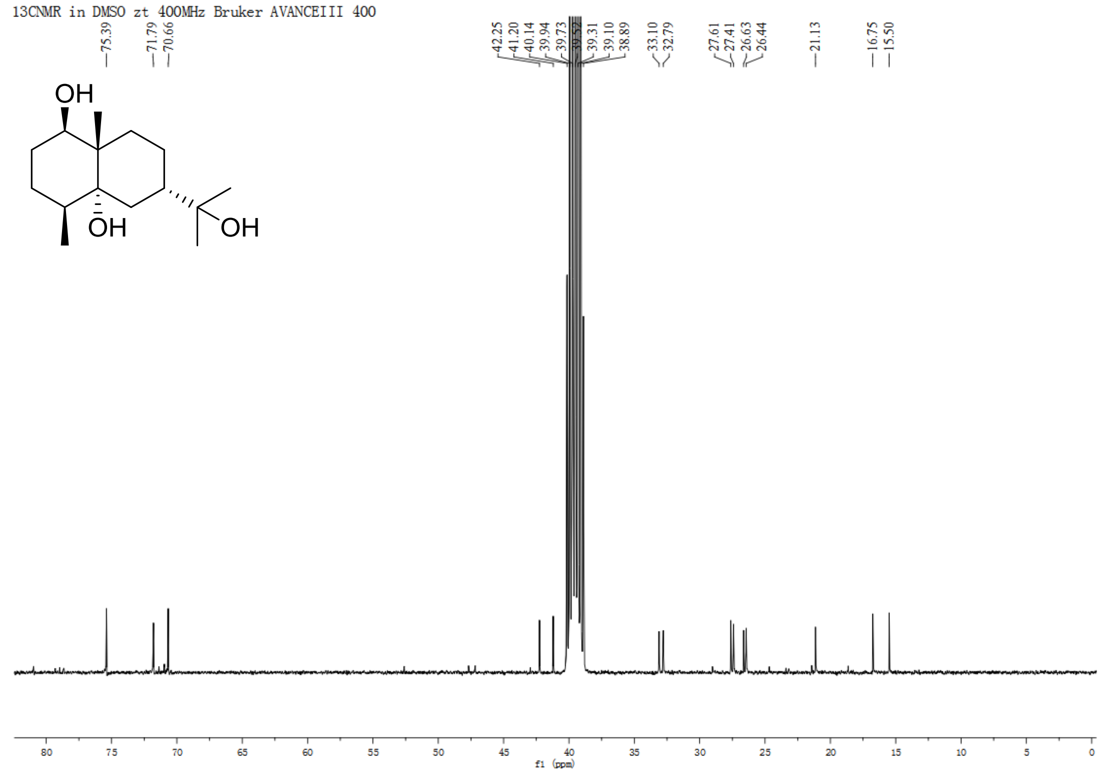

Figure S108. HMBC spectrum of compound **11** in DMSO-d_6

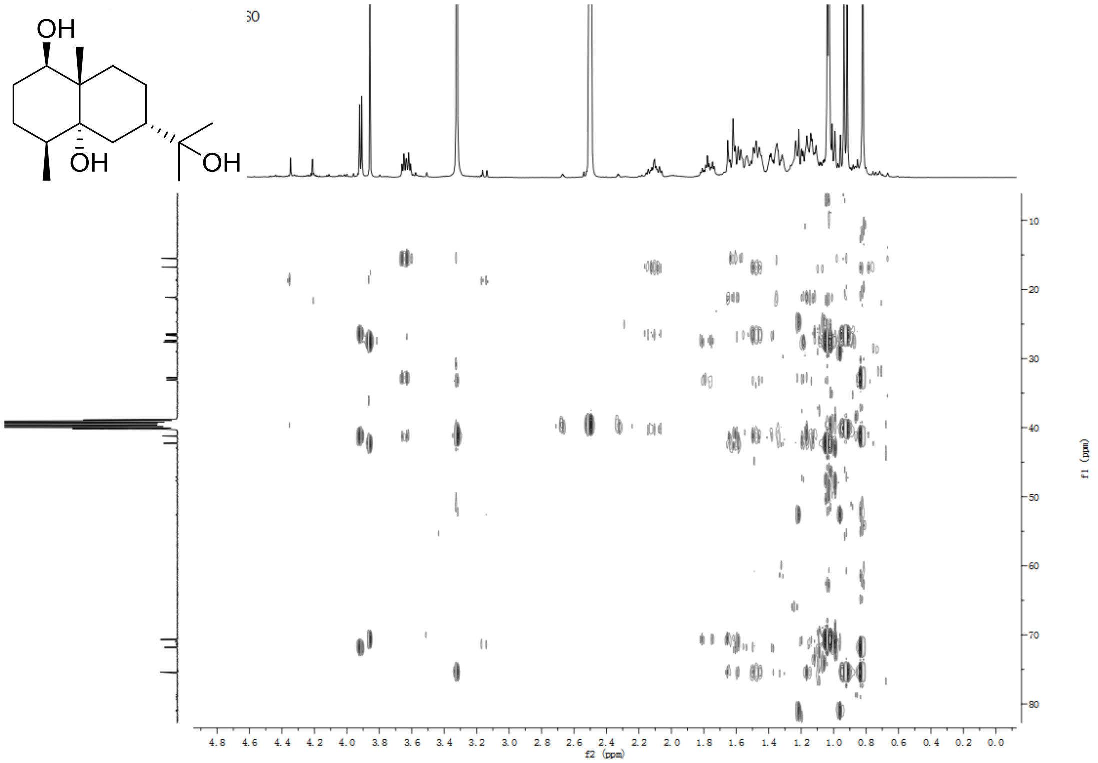

^{1}H NMR and ^{13}C NMR data of Compounds **11**

	**11**[a]		**11**[b]	
position	δ_C type	δ_H (*J* in Hz)	δ_C type	δ_H (*J* in Hz)
1	74.3, CH	3.88, dd (11.3, 4.9)	71.8, CH	3.63, dt (11.3, 4.8)
2	26.5, CH_2	1.66, m, Hα	26.4, CH_2	1.57, m, Hα [c]
		1.71, m, Hβ		1.59, m, Hβ [c]
3	26.9, CH_2	2.20, m, Hα	26.6, CH_2	2.10, m, Hα
		1.37, m, Hβ		1.07, m, Hβ [c]
4	41.4, CH	1.53, m	40.2, CH	1.47, m
5	77.6, C		75.4, C	
6	33.8, CH_2	1.84, m, Hα	33.1, CH_2	1.74, m, Hα [c]
		1.30, m, Hβ		1.20, m, Hβ [c]
7	43.3, CH	1.85, m	42.3, CH	1.77, m
8	21.7, CH_2	1.65, m, Hα	21.1, CH_2	1.55, m, Hα [c]
		1.32, m, Hβ		1.22, m, Hβ [c]
9	33.3, CH_2	1.72, m, Hα	32.8, CH_2	1.78, m, Hα
		1.57, m, Hβ		1.49, m, Hβ [c]
10	42.7, C		41.2, C	
11	72.9, C		70.7, C	
12	28.0, CH_3	1.22, s	27.6, CH_3	1.04, s
13	27.0, CH_3	1.20, s	27.4, CH_3	1.02, s
14	15.6, CH_3	1.00, s	15.5, CH_3	0.82, s
15	16.9, CH_3	1.04, d (7.7)	16.8, CH_3	0.93, d (7.7)
1-OH				3.92, d (5.3)
5-OH				3.32, s
11-OH				3.86, s

[a]Data were recorded at 400 MHz for proton and at 100 MHz for carbon in $CDCl_3$.
[b]Data were recorded at 400 MHz for proton and at 100 MHz for carbon in DMSO-d_6.
[c]Overlapped with other signals.

Figure S109. NOESY spectrum of compound **11** in DMSO-d_6

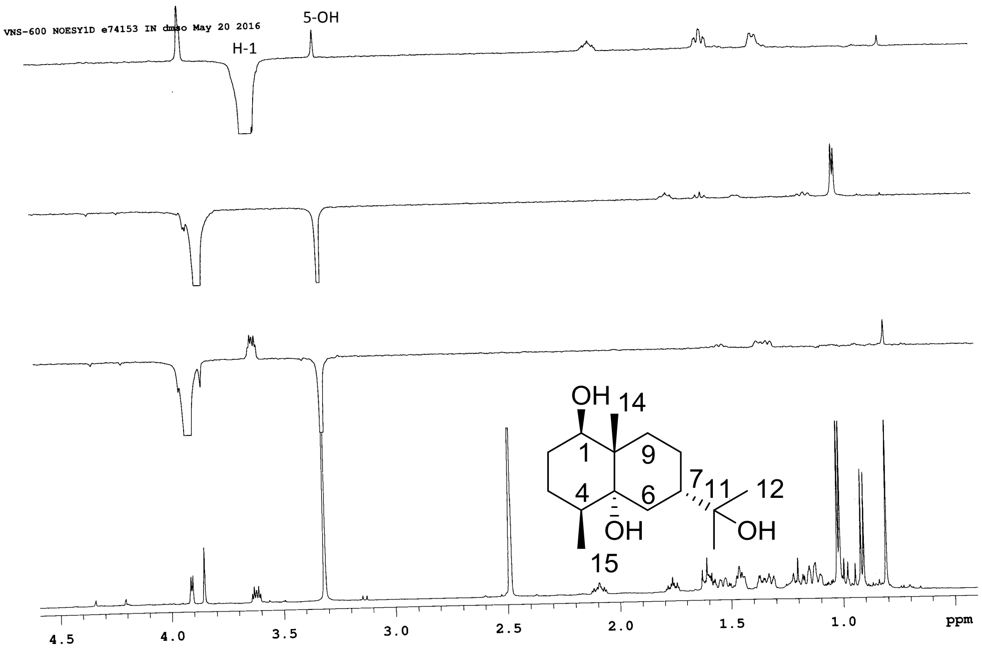

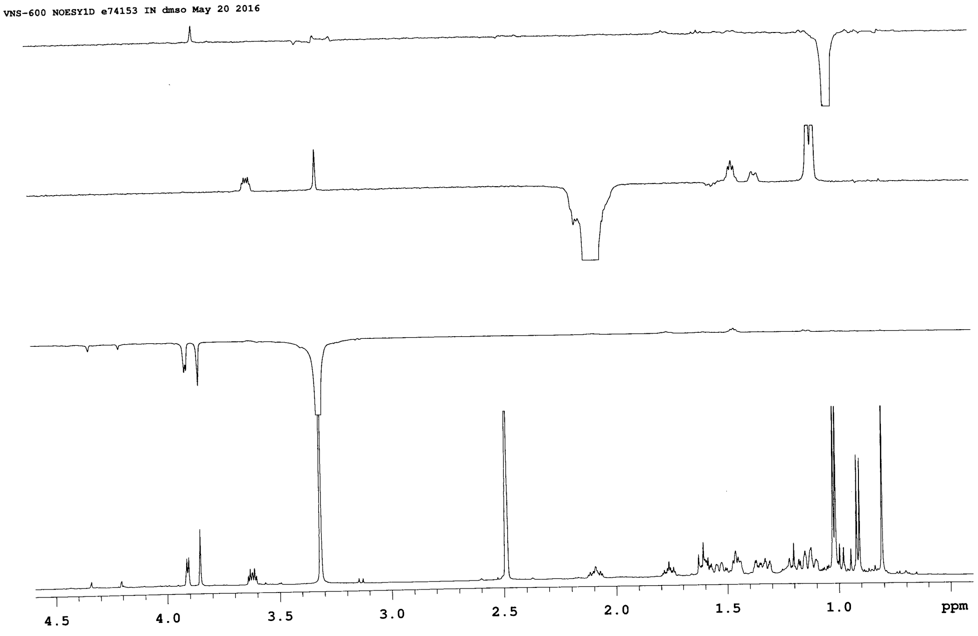

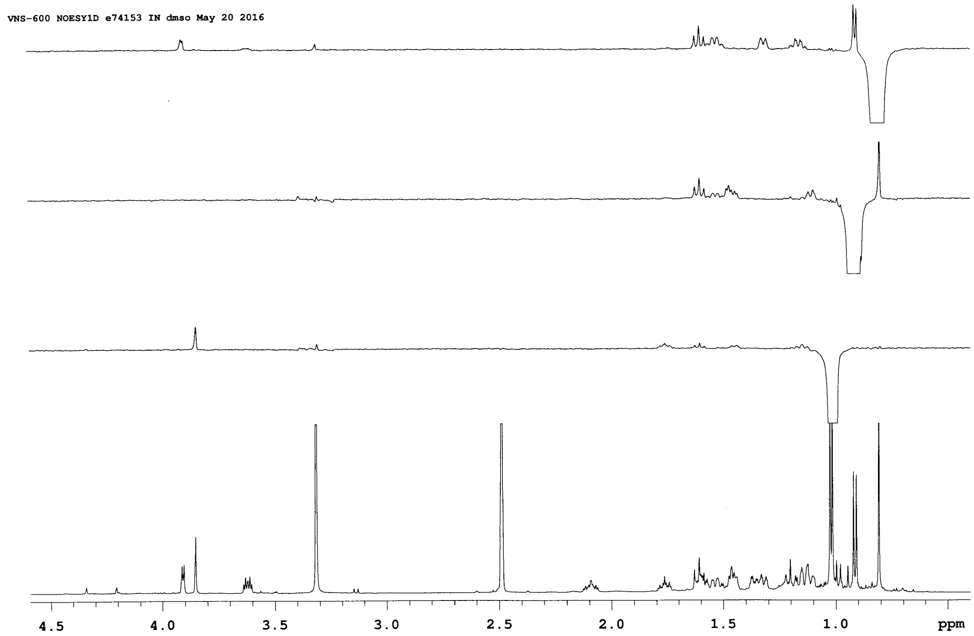

Table S1

Cytotoxicities of isolated compounds against five human cancer cell lines (IC_{50}, μM).

Compounds	IC_{50}				
	HCT116	NCI-H1650	BGC823	Daoy	HepG2
**1**	>50.0	>50.0	>50.0	>50.0	>50.0
**2**	>50.0	>50.0	>50.0	>50.0	>50.0
**3**	24.5	>50.0	>50.0	>50.0	>50.0
**4**	16.6	>50.0	49.9	31.1	27.9
**5**	>50.0	>50.0	>50.0	>50.0	>50.0
**6**	>50.0	28.9	>50.0	35.5	>50.0
**7**	>50.0	>50.0	>50.0	>50.0	>50.0
**8**	>50.0	>50.0	>50.0	>50.0	>50.0
**9**	12.2	>50.0	>50.0	>50.0	>50.0
**10**	>50.0	>50.0	>50.0	>50.0	>50.0
**11**	>50.0	>50.0	>50.0	>50.0	>50.0
**12**	>50.0	>50.0	>50.0	>50.0	>50.0
**13**	>50.0	>50.0	>50.0	>50.0	>50.0
**14**	>50.0	>50.0	>50.0	>50.0	>50.0
**Paclitaxel**	0.000809	0.945	0.00177	0.00933	0.0249

Table S2

Anti-HIV-1 activity of isolated compounds (IC_{50}, μM).

Compounds	HIV-1
**1**	NA
**2**	NA
**3**	NA
**4**	37.9
**5**	NA
**6**	NA
**7**	NA
**8**	NA
**9**	NA
**10**	NA
**11**	NA
**12**	NA
**13**	NA
**14**	NA
**Efavirenz**	0.002

NA=No activity
